# 

**Published:** 1889-01

**Authors:** 


### TOPICAL INDEX.

### 1889.

### A.

A comparison of standards (editorial), 305.
A new method of excision of the inferior dental nerve, with cases, Thomas Fillebrown, 215.
discussion, 218.
A new use for the phonograph (editorial), 754.
A professional holiday, George Cunningham, notice of, 117.
A step in advance (editorial), 560.
A study of arsenious acid, L. Ashley Faught, 293.
discussion, 298.
A study of electricity with the view of comprehending its application in dentistry, Howard E. Roberts, 703.
discussion of, 734.
A suggestion, Theodore F. Chupein, 582.
Abbott, Fr., on caries, Geo. S. Allan, 133, 136.
Abbott's microscopic specimens, 134.
protoplasmic theory, 130.
Abnormal condition of trophic centres, E. C. Kirk, 96.
incisors, L. C. Taylor, 647.
occlusion, S. H. Guilford, 153.
overbite, 152.
secretions, effects of, on the teeth, W. X. Sudduth, 354.
systemic conditions, C. N. Peirce, 333.
underbite, W. G. A. Bonwill, 14.
About face, E. B. Davis, 275.
Abutments in proximate surfaces, S. G. Perry, 148.
Abscess, W. H. Atkinson, 229.
after root-filling, W. G. A. Bonwill, 287.
Joseph Head, 278.
of the pulp, James Truman, 207.
Abscessed secretions of gingivæ in pyorrhœa alveolaris, Geo. S. Allan, 720.
socket, implantation in, L. C. Bryan, 567.
Absorption and reproduction in regulating, S. H. Guilford, 399.
in implanted tooth, E. C. Kirk, 95.
prevention of, G. L. Curtis, 88.
in the lower jaw, B. A. R. Ottolengui, 347.
of alveolar process, S. H. Guilford, 395.
of lactic acid by lime salts, Geo. S. Allan, 131.
under plates, T. S. Waters, 198.
work of bacteria in, G. L. Curtis, 87.
Abstract of Minnesota State Dental Law, 572.
Abuse of tooth-brush, case of, W. G. A. Bonwill, 364.
Accessories of decay, W. X. Sudduth, 354.
Accidental irritants in pyorrhœa alveolaris, Geo. S. Allan, 720.
Jas. Truman, 741.
Accommodation, power of, in the eye, and dental caries, A. P. Brubaker, 641.
Accretions of calculus as cause of interdental spaces, S. H. Guilford, 155.
Accumulations upon the teeth, causes of, C. N. Peirce, 334.
Acid abnormity of buccal fluids, C. N. Peirce, 333.
W. X. Sudduth, 354.
an, the initiative step in decay, Geo. S. Allan, 130.
foods, influence of, W. D. Miller, 311.
manufactory, the mouth an, Geo. S. Allan, 131.
medicines, influence of, W. D. Miller, 311.
reaction of saliva, W. D. Miller, 310.
saliva and caries, C. N. Peirce, 333.
and decay, W. X. Sudduth, 353.
secretions of mucous glands and caries, W. D. Miller, 311.
Acids, action of, upon the teeth, H. C. Register, 532.
in dental caries, Abbott on, Geo. S. Allan, 135.
in oral cavity, whence? Geo. S. Allan, 130.
in tooth destruction, Geo. S. Allan, 130.
Acidulating organisms, H. C. Register, 94.
Achievements, the, of our specialty, C. E. Francis, 59.
Aching pulps, G. W. Klump, 671.
Action of salts of lime in albumen, R. R. Andrews, 193.
of vaso-motor nerves, Jas. Truman, 2.
Active agents in the causation of decay, W. X. Sudduth, 354.
Acute otalgia of dental origin, E. C. Kirk, 663.
pulpitis, Jas. Truman, 208, 214.
A dark period in professional life, S. G. Perry, 68.
Address of Dr. H. C. Register, president Pennsylvania State Dental Society, 528.
of welcome, Odontological Society of Pennsylvania, C. J. Essig, 164.
Adhesiveness of gold, W. H. Dwinelle, 176.

A diluted muddle, A. G. Bennett, 592.
Adjustable jaws for clamps, W. H. Rollins, 186.
Advancement in dentistry, S. B. Palmer, 386.
Advances, scientific, C. C. Barker, 257.
Advantages of cotton in root-filling, Jos. Head, 277.
of matrices, S. H. Guilford, 194.
of wider knowledge of anatomy, A. P. Brubaker, 640.
L. A. Faught, 662.
W. E. Magill, 667.
Æsthetic prosthesis, J. M. Edmunds, 656.
Affinities, S. B. Palmer, 384.
Agents that destroy teeth, Geo. S. Allan, 130.
Aggressive journalism (editorial), 565.
Aid of mouth-mirrors in provisional cavities, S. G. Perry, 139.
Aide-mémoire du Chirurgien Dentiste, par M. Paul Du Bois, notice of, 691.
Air, compressed heated, Jas. Truman, 213.
Allan, Geo. S., on Abbott's theory of caries, 133.
on acids in caries, 130.
on artificial caries, 132.
on amœloblasts, 166.
on photo-micrographs, 132.
on lantern exhibit, 166.
on pyorrhœa alveolaris, 712.
on the etiology of caries, 129.
on the germ theory, 130.
on the work of Dr. Miller, 131.
Albumen, action of salts of lime in, R. R. Andrews, 193.
Alchemy, H. C. Register, 529.
Alloy and copper amalgam, joining, J. W. Russell, 654.
Alum carmine, staining with, R. R. Andrews, 195.
Alumni Association, Boston Dental College, resolutions on the death of Dr. Rideout, 765.
Alveolar abscess, R. B. Adair, 701.
Jos. Head, 278, 292.
B. A. R. Ottolengui, 340.
Wm. H. Trueman, 455.
Jas. Truman, 210.
after root-canal filling, Jos. Head, 292.
discharging through antrum and nose, case of, E. C. Kirk, 422.
from cotton root-fillings, Jos. Head, 277.
from lateral incisors, B. A. R. Ottolengui, 340.
from leaking root-fillings, Joseph Head, 369.
independent of tooth, Wm. H. Trueman, 455.
years after devitalization, Wm. H. Trueman, 455.
necrosis, splint for loose teeth, E. H. Angle, 379.
process, the, S. H. Guilford, 395.
Alveolar septum, thickening of, S. H. Guilford, 152.
wall, reproduction of, L. C. Bryan, 568.
Alveoli of deciduous teeth, S. H. Guilford, 395.
Alveolus, absorption and reproduction of, in regulating, S. H. Guilford, 399.
fracturing the, Jos. Head, 280.
Alvord, Mr., experience with, G. L. Curtis, 482.
Geo. A. Maxfield, 483.
A.M. degree in dental schools, E. H. Smith, 322.
Amalgam, W. C. Barrett, 242.
F. S. Bassett, 603.
C. S. Beck, 607.
Alonzo Boice, 607.
Chas. S. Butler, 416.
W. S. Elliott, 730.
L. A. Faught, 602.
Jas. S. King, 605.
S. B. Palmer, 390.
S. G. Perry, 141.
G. L. Robb, 604.
G. F. Root, 587.
antiseptic properties of, S. B. Palmer, 390.
artistic capabilities of, F. S. Bassett, 602.
as root-canal filling, G. F. Root, 587.
as a tooth-saver, Chas. S. Butler, 416.
bevelling cavity borders for, F. S. Bassett, 601.
care in working, A. Boice, 607.
crystallization of, W. H. Atkinson, 748.
W. S. Elliott, 730.
displacing in removal of clamp, Jas. S. King, 605.
of matrix, F. S. Bassett, 603.
L. A. Faught, 602.
G. L. Root, 604.
of rubber dam, J. R. C. Ward, 605.
excess of, at cervical borders, F. S. Bassett, 600.
in proximate cavities, S. G. Perry, 141.
washing, C. S. Beck, 607.
J. S. King, 607.
American and European, J. W. Russell, 652.
A matrix crank, W. B. Miller, 603.
Amœloblasts, the, Geo. S. Allan, 136.
function of, C. N. Peirce, 330.
American Academy of Dental Science, annual meeting of, 58.
exhibit of, 158.
report of proceedings of, 215.
amalgams, J. W. Russell, 652.
and Italian students, W. X. Sudduth, 110.
College of Dental Surgery, commencement of, 300.
Dental Association (editorial), 626.
memorial meeting (editorial), 765.
John S. Marshall, 695.
popularity of, W. E. Magill, 636.
Dental Trade Association (editorial), 429.

American Dental Trade Association, an explanation, T. R. Morrison, 441.
Dental Society of Europe, meeting of, 1891, 694.
officers elected, 694.
dentist, the, in England, Geo. Cunningham, 507.
dentistry, recent advancement, E. H. Smith, 320.
the leaders in, E. B. Davis, 271.
high-pressure system, W. X. Sudduth, 110.
Medical Association, 255.
dental section of, Harlan on, John S. Marshall, 696.
school system and the teeth, R. C. Newton, 581.
system of dentistry, W. Storer How, 468.
teeth, Jas. Truman, 612.
Tooth Crown Company in England, 507.
Ammonia and artificial dentures, W. H. Morgan, 125.
Amœba, development of, W. X. Sudduth, 545.
the human, Wm. H. Atkinson, 49.
W. X. Sudduth, 542.
Amount of power to be applied in regulating, S. H. Guilford, 400.
Ampère, definition of, H. E. Roberts, 704.
Amputation of affected molar root in pyorrhœa alveolaris, Geo. S. Allan, 722.
Amylaceous matters in the mouth, Geo. S. Allan, 130.
An interesting case (editorial), 183.
Analytic work of Dr. Miller, Geo. S. Allan, 131.
Anatomical articulator, W. G. A. Bonwill, 11, 491.
Anatomy, desirability of wider knowledge of, A. P. Brubaker, 646.
L. A. Faught, 662.
of the maxillæ, B. A. R. Ottolengui, 336.
study of, by the dental student, A. P. Brubaker, 640.
Anchorage in implantation, G. L. Curtis, 87.
in regulating, E. H. Angle, 324.
Ancient bridge-work, T. S. Waters, 197.
Anchylosis in implantation, L. C. Bryan, 568.
B. H. Catching, 191.
W. X. Sudduth, 97.
Anderson, John, and plate-work, J. Hayhurst, 102.
Andrews, R. R., on action of lime salts in albumen, 193.
on arrested decay, 169.
on artificial caries, 169.
on calco-globulin, 193, 517.
on calco-spherites, 169, 194.
on changes in dentine structure, 169.
on circulation in tooth substance, 169.
on cracks in approximal surfaces, 518.
on lantern exhibit, 166.
Andrews, R. R., on oxychloride of zinc fillings and tooth-hardening, 169.
on pits and fissures of the enamel, 511.
on the border-land of calcification, 193.
Anæsthetics, W. B. Macleod, 758.
L. A. Faught, 34.
Angle, Ed. H., on expanding the arch, 326.
occipital bandage, 324.
on regulating appliances, 323.
on splint for implanted or loose teeth, 379.
Animals, lower, teeth of, —— Bailey, 41.
J. T. Codman, 29.
W. X. Sudduth, 42.
S. C. G. Watkins, 42.
Annual of the Universal Medical Sciences, edited by Chas. E. Sajous, notice of, 689.
Anterior buccal canals of molars, filling, Jos. Head, 278.
palatal canal, location of, B. A. R. Ottolengui, 339.
teeth, protrusion of, E. H. Angle, 323.
S. H. Guilford, 151.
Anthrax bacillus, W. X. Sudduth, 545, 556.
Carl Heitzmann, 553.
Anticipating decay, S. G. Perry, 73.
malformations, C. N. Peirce, 335.
Antidotal treatment, L. A. Faught, 293.
Antidotes to decay, C. N. Peirce, 335.
Antipyrin, abuse of (editorial), 500.
Antisepsis, G. V. Black, 315.
W. X. Sudduth, 94.
Jas. Truman, 93.
in air-passages, Jas. Truman, 93.
in implantation, G. L. Curtis, 87.
W. X. Sudduth, 97.
in pulp canals, S. G. Perry, 222.
in root-canal treatment, C. E. Francis, 228.
in sick rooms, Jas. Truman, 93.
of the mouth, Jas. Truman, 93.
with atomizer, H. C. Register, 93.
Antiseptic agents, Jas. Truman, 210.
copper amalgam as an, E. Parmly Brown, 674.
sulphate of quinia, Jas. Truman, 742.
dressing incorporated in copper amalgam, H. C. Register, 291.
mouth-washes, C. N. Peirce, 91.
spray, Jas. Truman, 93.
Carl Seiler's, 192.
tobacco as an, E. C. Kirk, 93.
treatment in implantation, E. C. Kirk, 367.
in pulp removal, James Truman, 207.
of canals, Jas. Truman, 208.
Antiseptics, absorption of, by tooth substance, L. C. Bryan, 569.
indebtedness to, C. N. Peirce, 91.
in dental practice, T. H. Parramore, 393.
in intentional pulp devitalization, Wm. H. Trueman, 456.
in replantation, Weld, 337.
in root-canal treatment, H. C. Register, 291.

Antiseptics in root-canal treatment, G. F. Root, 586.
Jas. Truman, 208.
Listerine, 94.
Antral abscess, S. H. Guilford, 425.
cases of, L. A. Faught, 424.
catarrh, Harrison Allen, 422.
E. C. Kirk, 422, 427.
disease, Ambler Tees, 425.
cure of, without extraction, J. N Farrar, 457.
use of aspirator, J. N. Farrar, 457.
Antrum, diseases of, spray-syringe for, J. N. Farrar, 457.
locating, the, in implantation, B. A. R. Ottolengui, 341.
penetration of, in implantation, E. C. Kirk, 421.
puncturing the, B. A. R. Ottolengui, 368.
Jas. Truman, 369.
relationships of, L. A. Faught, 424.
Apex, movement of, in regulating, S. H. Guilford, 396.
Apical end fillings, Jos. Head, 287.
Appeal, the Johnstown calamity, 440.
Appearance of mucous membrane from arsenious acid, L. A. Faught, 297.
of rodent ulcers, L. A. Faught, 297.
Apple, the primeval, and caries, Geo. W. McElhaney, 637.
Appliance for holding implanted teeth, E. H. Angle, 379.
for treatment of prominent superior oral teeth, E. H. Angle, 324.
Appointment book, R. I. Pearson's (editorial), 116.
Appropriators, C. J. Essig, 165.
Approximal cavities, Kingsbury, 173.
amalgam for, S. G. Perry, 141.
Arc light, the, in dental offices, H. E. Roberts, 709.
Arch, expanding the, E. H. Angle, 326.
the great, of bicuspids, S. G. Perry, 139.
Architectural models, S. G. Perry, 71.
Aromatic sulphuric acid, Wm. H. Atkinson, 177.
Arsenic, action of, Jas. Truman, 208.
and immediate root-filling, Jas. Truman, 207.
for sensitive dentine, Wm. H. Trueman, 454.
in pulp devitalization, G. F. Root, 585.
Wm. H. Trueman, 454.
in pulp treatment, Jas. Truman, 204.
in rubber dam, L. A. Faught, 296.
reapplication of, G. F. Root, 585.
Arsenical ulcers, location of, L. A. Faught, 297.
Arsenical ulceration, L. A. Faught, 295.
after removal of old plugs, L. A. Faught, 296.
duration of, L. A. Faught, 298.
from perforated tooth, L. A. Faught, 296.
from rubber dam, L. A. Faught, 295.
Arsenical Ulceration, results of, L. A. Faught, 298.
treatment of, C. M. Dixon, 299.
L. A. Faught, 297.
Arsenious acid, a study of, L. A. Faught, 293.
access to periosteum of, L. A. Faught, 297.
and cosmoline, A. B. Harrower, 298.
and lanoline, formula, A. B. Harrower, 298.
appearance of mucous membrane from, L. A. Faught, 297.
checking absorption of, L. A. Faught, 298.
destruction of tissues by, L. A. Faught, 297.
formula, L. A. Faught, 294.
liability of accident, L. A. Faught, 295.
local effect of, L. A. Faught, 297.
on cotton and creosote, C. M. Dixon, 299.
pathological results of, L. A. Faught, 294.
precautions in use of, L. A. Faught, 295.
qualities of, Flagg, 293.
quantity used, Alonzo Boice, 298.
L. A. Faught, 294.
F. J. S. Gorgas, 294.
Arsenite of calcium in rubber dam, L. A. Faught, 296.
Arterial connection of enamel, C. N. Peirce, 331.
Arteries supplying the teeth, B. A. R. Ottolengui, 347.
Arteriole, rupture of an, B. A. R. Ottolengui, 343.
Arthur method, the, J. N. Crouse, 175.
E. J. Dunning, 74.
Kingsbury, 173.
Benj. Lord, 74.
S. G. Perry, 68.
separations in proximal cavities, S. G. Perry, 147.
Articulation of crowns, L. C. Bryan, 61.
restoration of, H. H. Edwards, 693.
Artificial caries, Geo. S. Allan, 138.
R. R. Andrews, 169.
W. D. Miller, 310.
H. C. Register, 532.
of W. D. Miller, Geo. S. Allan, 132.
crowns in implantation, G. L. Curtis, 86.
dentures, hygienic relations of, E. C. Kirk, 199.
to clean, W. H. Morgan, 125.
teeth, J. Hayhurst, 102.
carved blocks, J. Hayhurst, 103.
composition of, Dr. Fowler, 105.
materials, W. H. Dwinelle, 107.
J. Hayhurst, 102.
Artistic adaptation in prosthetic dentistry, Comstock, 317.
capabilities of amalgam, F. S. Bassett, 602.

Ash & Son, exhibit, 236.
Ash's long-pin teeth, L. C. Taylor, 648.
Asphyxia, from nitrous oxide, W. B. Macleod, 758.
Aspirator, use of, in antral disease, J. N. Farrar, 457.
Associated effort, S. B. Palmer, 391.
Astrology, H. C. Register, 529.
A three years' graded course (editorial), 563.
Athletic contests, training for, and the teeth, D. W. Parker, 580.
Ataxia, hereditary, Hutchinson's teeth in, R. C. Newton, 576.
Atkinson, Wm. H. and contour fillings, S. G. Perry, 72.
complimentary dinner to, 248.
first teeth made by, 105.
on Dr. Codman's paper on shock, 37.
on Dr. Elliott's paper on light to the gentiles, 747.
on Dr. Newton's paper on the teeth as a factor, etc., 617.
on Dr. Sudduth's paper on the individuality of the cell, 553.
on nutrition, 37.
on thoroughness, 460.
Atmospheric influences on dead pulps, H. C. Register, 290.
Atomizer for antiseptics, H. C. Register, 93.
in antisepsis for children, H. C. Register, 94.
in antral treatment, H. C. Register, 427.
Atomization of the mouth and teeth, H. C. Register, 533.
Atrophied ridge in implantation, G. L. Curtis, 86.
Attachment of implanted teeth, L. C. Bryan, 567.
Attention to trifles, B. A. R. Ottolengui, 338.
Augerstien's home gymnastics, notice of, 433.
Aural diseases from irritation of the dental nerves, A. P. Brubaker, 641.
Australia, condition of dentistry in, Alfred Burns, 629.
Automatic gas furnace, C. C. Carroll, 237.
mallets, E. C. Kirk, 520.
power mallet, E. C. Kirk, 519.

B.

Babcock, Wm. D., on necrosis of the palate and superior maxillary bones, the result of alveolar abscess and a rubber plate, 464.
Bacilli, C. C. Barker, 258.
Backward movement of teeth in regulating, S. H. Guilford, 399.
Bacteria, Geo. S. Allan, 132.
C. C. Barker, 257.
E. A. Bogue, 312.
E. M. Hunter, 262.
C. N. Peirce, 168.
Bacteria, H. C. Register, 529.
and caries, Geo. S. Allan, 132.
and disease, C. C. Barker, 258.
E. M. Hunter, 262.
Semmola, 266.
as factors in pulp irritation, Jas. Truman, 208.
as scavengers, E. A. Bogue, 313.
classification of, C. C. Barker, 258.
culture in oral cavity, H. C. Register, 94, 532.
food supply of, Geo. S. Allan, 131.
in absorption of root, G. L. Curtis, 87.
in pyorrhœa alveolaris, Geo. S. Allan, 720.
Jas. Truman, 741.
W. X. Sudduth, 744.
rapid multiplication of, C. C. Barker, 257.
*rôle* of, E. M. Hunter, 262.
value of, C. C. Barker, 258.
waste products of, Geo. S. Allan, 130.
Bacterial origin of caries, Geo. S. Allan, 132.
Bacterian pathology, A. L. Loomis, 261.
world, the, C. C. Barker, 257.
Bacteriology, C. C. Barker, 259.
Bacteriopathy, H. C. Register, 529.
Bad breath in dogs, Watkins, 42.
teeth, bad form, R. C. Newton, 582.
roots, to extract, Preston, 162.
Baltimore College of Dental Surgery, forty-ninth annual commencement of, 300.
Band-and-spur retaining appliance, S. H. Guilford, 413.
Banding teeth in regulating, E. H. Angle, 324.
Bank-account of the dental profession, J. N. Crouse, 480.
Barbs, to cut, on very fine broach, W. G. A. Bonwill, 495.
Barker, C. C., on the bacterian world, 257.
on scientific advances, 257.
Base plate gutta-percha, Joseph Head, 279.
as root-canal filling, J. D. Patterson, 286.
Basis for comparison of standards (editorial), 305.
of practice, the, E. H. Smith, 319.
substance, the, in calcified products (editorial), 239.
Bassett, F. S., on arsenious acid, 299.
on the use of the matrix with proximal amalgam fillings, 600.
Battery, cell-power of, H. E. Roberts, 706.
Battle-fields, teeth from, L. C. Bryan, 246.
Beaks, forms of, in new forceps, V. C. Pond, 159.
Beginning of life, the, W. X. Sudduth, 51.
Bell-shaped pluggers, S. G. Perry, 140.
Beneficial effects of lime-water, cases of, Ambler Tees, 364.
Benefits of exhibits, Geo. W. Lovejoy, 405.
of implantation, G. L. Curtis, 88.
Benign organisms, C. N. Peirce, 168.
Bennett, A. G., on allaying fear in young patients, 36.

Bennett, A. G., on dental literature, 588.
on root-canal filling, 288.
F. J., on certain points connected with the structure of dentine, 195.
experiments with dentine, R. R. Andrews on, 196.
Benzoic sulphide of sodium, E. Heckel, 126.
Bevelled joints for forceps, Ham, 161.
Bevelling cavity borders for amalgam fillings, F. S. Bassett, 601.
Bibliography, 117, 432, 689.
of dental literature, W. Storer How, 469.
Bichloride of mercury, C. S. Beck, 672.
E. C. Kirk, 367.
G. F. Root, 587.
W. X. Sudduth, 94.
A. H. Thompson, 315.
Geo. H. Winkler, 702.
in implantation, E. C. Kirk, 367.
in root-canal treatment, G. F. Root, 587.
W. X. Sudduth, 94.
Bicuspid and molar root-canals, number of, Jos. Head, 277.
root-canals, treatment of, Jos. Head, 277.
Bicuspids, approximal fillings in, S. G. Perry, 139.
lower, implantation of, B. A. R. Ottolengui, 346.
superior, precautions in implantation, B. A. R. Ottolengui, 341.
Bing's bridge teeth, A. G. Bennett, 233.
J. M. Edmunds, 656.
method, Beer's improvement on, J. M. Edmunds, 656.
Webb's improvement on, J. M. Edmunds, 656.
Black, G. V., on pyorrhœa alveolaris, Geo. S. Allan, 712, 745.
Jas. Truman, 739.
on pits in the enamel, R. R. Andrews, 516.
Blackened teeth from bichloride of mercury, A. H. Thompson, 315.
Blackmail, S. B. Palmer, 386.
Black wax, L. C. Bryan, 62.
Blair, H. C., Son's exhibit, 236.
Bleeding gums in pyorrhœa alveolaris, Jas. Truman, 741.
Blindness from wedging the teeth, W. H. Fundenberg, 667.
Blood, microscopic appearance of the, W. X. Sudduth, 542.
serum, action of, upon dentine, W. D. Miller, 311.
Board of Censors, New York State Dental Society, 255.
Bodecker's discoveries, Fr. Abbott, 133.
Bogue, E. A., description of process for stamping plates by hydraulic press, 391.
Boice, Alonzo, on amalgam, 607.
on arsenious acid, 298.
on root-canal filling, 287.
Bone cells, proliferation of, in implantation, G. L. Curtis, 87.
Bone teeth and shells, development of, Rainie, 193.
Bonwill, W. G. A., on regulators and methods of correcting irregularities, 10, 76.
on orthodontia, 10.
on rapid breathing as a pain obtunder, 492.
on root-canal filling, 287.
electro-magnetic mallet, E. C. Kirk, 520.
engine mechanical mallet, E. C. Kirk, 520.
mechanical mallet, E. C. Kirk, 538.
S. G. Perry, 147.
method of packing gold foil, E. C. Kirk, 519, 699.
discussion, 536.
preparation of foils for, E. C. Kirk, 520.
patents, 486.
discussion, 495.
Bonwill-Kirk points, E. C. Kirk, 539.
Bonwill's anatomical articulator, 491.
free gifts to the profession, 492.
nerve-broach reducer, 487.
new matrix, 490.
oval-faced pluggers, 492.
right-angle plugger, 490.
spiral spring, 491.
Border-land of calcification, R. R. Andrews, 193.
Bony anchylosis in implantation, L. C. Bryan, 668.
B. H. Catching, 191.
W. X. Sudduth, 97.
tumors, Martindale, 316.
Box-cap and split post in bridge-work, T. S. Waters, 201, 237.
Brace for support of loose teeth, Geo. S. Allan, 722.
Brain, effect of, on circulation, Marshall Hall, 3.
Brasseur, Dr. E., E. A. Bogue on, 182.
letter from, 377.
Brass wire ligatures, E. C. Kirk, 368.
Brazil, letter from, C. R. Fletcher, 124.
Breakage of pulpless teeth, J. Morgan Howe, 697.
Breaking a root, S. B. Palmer, 385.
Breathing, philosophy of, Wm. H. Atkinson, 48.
Bridge and crown patents, W. X. Sudduth, 496.
intimidation, W. X. Sudduth, 254.
Bridge-work, Wm. H. Atkinson, 235.
A. G. Bennett, 232.
E. P. Brown, 678.
C. C. Carroll, 685.
J. Y. Crawford, 127.
W. H. Dwinelle, 232.
J. M. Edmunds, 655.
Truax, 681.
T. S. Waters, 197, 237.
S. G. C. Watkins, 678.
R. B. Winder, 238.
air-tight caps, Pinney, 683.
J. M. Edmunds, 683.

Bridge-work, aluminum, C. C. Carroll, 685.
box-cap and split-post in, T. S. Waters, 201, 237.
gutta-percha anchorage, A. G. Bennett, 234.
improvements in methods, T. S. Waters, 197.
invention of, W. H. Dwinelle, 232.
on loose teeth, S. G. C. Watkins, 678.
E. Parmly Brown, 678.
on rubber and celluloid, E. Parmly Brown, 677.
Parr's system, W. H. Dwinelle, 232.
permanent, A. G. Bennett, 232.
W. H. Dwinelle, 232.
T. S. Waters, 197.
hygienic aspect of, A. G. Bennett, 233.
T. S. Waters, 197.
preparation of roots and crowns, T. S. Waters, 203.
removable, A. G. Bennett, 233.
W. H. Dwinelle, 232.
J. M. Edmunds, 655.
T. S. Waters, 197, 237.
R. B. Winder, 238.
the work of the future, T. S. Waters, 203.
repairing, W. H. Dwinelle, 232.
spring catch in gold crown, T. S. Waters, 199.
spring system, A. G. Bennett, 234.
telescoping, C. C. Carroll, 685.
W. H. Dwinelle, 232.
J. M. Edmunds, 685.
theoretically and practically, C. C. Carroll, 685.
Waters's system, W. H. Dwinelle, 232.
Brine for preservation of extracted teeth, Theo. F. Chupein, 583.
Bright's disease and pyorrhœa alveolaris, M. S. Rhein, 744.
sensitive teeth in, R. C. Newton, 621.
the teeth in, Jas. Truman, 612.
R. C. Newton, 577.
British Dental Association, W. G. A. Bonwill, 628.
Broach, the, in pulp extirpation, G. F. Root, 584.
reducer, W. G. A. Bonwill, 487.
Broken down blood corpuscles, W. H. Dwinelle, 170.
incisors, shell-cap for, restoration of, L. C. Taylor, 648.
pipe-stems in dentine, R. R. Andrews, 518.
Brooklyn Dental Society and the Dental Protective Association, 766.
Brophy's matrix, S. G. Perry, 146.
Brown, E. Parmly, on bridge-work, 678.
on Edmund's paper on removable bridge-work, 677.
Brown-Séquard elixir (editorial), 622.
Brubaker, Albert P., on reflex effects of dental irritation, 639.
Bryan, L. C., letters from, 61, 189, 245, 567, 694, 760.
Bryan, L. C., on articulating wax, 61.
on binding and filing dental journals, 760.
on dental patents, 246.
on implantation, 245, 567.
on topical index, 189, 246.
Buccal roots of molars, filling, Jas. Truman, 285.
secretions, varying quality of, Jas. Truman, 285.
surfaces, treatment of, S. G. Perry, 66.
Bugs and worms and caries, W. C. Browne, 637.
Bunsen battery for dental mallet, C. S. Beck, 735.
Burrowing pus from lateral incisor abscess, B. A. R. Ottolengui, 340.
Burs, flat narrow shanks, S. G. Perry, 140.
for proximate surfaces, S. G. Perry, 140.
Business courtesies, Welch Dental Company (editorial), 116.
By-paths (editorial), W. C. B., 241.

C.

Calciferous elements, insufficient supply of, in the blood, C. N. Peirce, 334.
Jas. Truman, 355.
supply of, in the blood, C. N. Peirce, 334.
Calcific deposits, S. B. Palmer, 388.
Calcification, border-land of, R. R. Andrews, 193.
Calcified tubuli, R. R. Andrews, 169.
Calco-globulin, R. R. Andrews, 193, 517.
G. V. Black, 317.
appearance of, R. R. Andrews, 166, 195.
(editorial), 239,
Calco-spherites, R. R. Andrews, 169, 194.
(editorial), 240.
Calculi, formation of (editorial), 239.
Calculus, accretions of, a cause of interdental spaces, S. H. Guilford, 155.
as a cause of voluntary tooth-movement, S. H. Guilford, 156.
on roots, S. H. Guilford, 156.
Calvert's teeth, Jas. Truman, 104.
Campho-phenique, W. H. Atkinson, 748.
W. S. Elliott, 727.
Capillarity, W. H. Atkinson, 177.
Capillary attraction in approximal fillings, S. G. Perry, 139.
Capping pulps, J. N. Crouse, 226.
W. H. Dwinelle, 229.
C. E. Francis, 227.
Jas. Truman, 231.
sterilized sponge for, W. S. Elliott, 475.
S. H. Guilford, 474.
J. Bond Littig, 476.
T. H. Parramore, 393, 474.
I. J. Weatherby, 476.
Carbolic acid, J. N. Crouse, 226.
W. X. Sudduth, 94.
Jas. Truman, 213.

Carbolic acid, action of, after arsenic, Jas. Truman, 208.
in pulp exposure, J. N. Crouse, 226.
Carbolized cotton, Jos. Head, 281, 608.
J. D. Patterson, 286.
cosmoline and cotton as root-canal filling, Jos. Head, 608.
E. C. Kirk, 292.
W. B. Miller, 610.
Carbonate of copper, protective power of, Geo. F. Waters, 314.
Carcinoma, Wm. H. Atkinson, 50.
Care of proximate surfaces, S. G. Perry, 66.
of the teeth in sickness, Ed. C. Briggs, 633.
Caries a chemical process, Fr. Abbott, 136.
an inflammatory process (Abbott), Geo. S. Allan, 134.
arresting the progress of, R. R. Andrews, 169.
C. N. Peirce, 329.
artificial, of Dr. Miller, Geo. S. Allan, 132.
chemical theory of, G. W. McElhaney, 637.
definition of, C. N. Peirce, 329.
etiology of, Geo. S. Allan, 129.
germ theory of, Geo. S. Allan, 130.
W. C. Browne, 637.
W. C. Wardlaw, 637.
in cementum, W. X. Sudduth, 353.
in dead teeth, Fr. Abbott, 138.
in dog's teeth, W. D. Miller, 244.
in horse's teeth, W. D. Miller, 244.
inflammatory theory of, Geo. S. Allan, 134.
of dead dentine, W. D. Miller, 310.
of dead teeth, Geo. S. Allan, 138.
predisposing causes of, Geo. S. Allan, 129.
W. C. Wardlaw, 701.
protoplasmic theory, Geo. S. Allan, 130.
*vs.* decay, E. C. Kirk, 358.
C. N. Peirce, 360.
W. X. Sudduth, 352, 359.
where it commences, Geo. S. Allan, 130.
Carious dentine, micro-organisms in, Geo. S. Allan, 131.
process, the, Geo. S. Allan, 138.
teeth and reflex disorders, A. P. Brubaker, 644.
Carroll, C. C., on bridge-work, 685.
Cartilage, microscopic appearance of, W. X. Sudduth, 543.
Case of deformed central incisors, L. C. Taylor, 647.
of implantation, B. A. R. Ottolengui, 342.
of serious hemorrhage in implantation, B. A. R. Ottolengui, 343.
Catarrh and rubber plates, W. D. Babcock, 465.
Catarrhal condition in pyorrhœa alveolaris, W. X. Sudduth, 178.
inflammation of the ear-chambers of dental origin, A. P. Brubaker, 642.
Catarrhal nature of oral affections, Harrison Allen, 688.
process in pyorrhœa alveolaris, W. X Sudduth, 743.
Catholics, diet of, J. T. Codman, 94.
Causal relations between diseases of the eye and the teeth, A. P. Brubaker, 640.
Cause and effect, S. B. Palmer, 383.
of mouth-breathing, R. C. Newton, 579.
Causes beyond control, Wm. H. Trueman, 456.
of decay, J. T. Codman, 22.
L. A. Faught, 354.
of failure in implantation, G. L. Curtis, 85.
of prominent superior oral teeth, E. H. Angle, 323.
S. H. Guilford, 152.
Cavities in tooth structure: why? Geo. S. Allan, 130.
utilizing in regulating, W. G. A. Bonwill, 80.
Cell, definition of, Wm. H. Atkinson, 553.
Drysdale's definition of, 551.
life, Atkinson, 46.
Heitzmann, 51.
Ottolengui, 51.
Sudduth, 46.
Truman, 50.
theory, the, Atkinson, 553.
Heitzmann, 551.
Sudduth, 543.
Cellular morphology, W. X. Sudduth, 540.
structure of alveolus, S. H. Guilford, 395.
Cement root-fillings, Jos. Head, 279.
Cemental decay, C. N. Peirce, 360.
Cementoblasts, S. H. Guilford, 397.
Cementum, caries in, C. N. Peirce, 332.
W. X. Sudduth, 353.
nutrition of, C. N. Peirce, 332.
structural arrangement of, C. N. Peirce, 332.
Censor, the, of the profession (editorial), 687.
Central incisors, protrusion of, E. H. Angle, 323.
W. G. A. Bonwill, 81.
J. N. Farrar, 271.
S. E. Gilbert, 373.
rotation of, E. H. Angle, 328.
superior, implantation of, B. A. R. Ottolengui, 338.
Tennessee College, Meharry dental department of, commencement of, 304.
Cervical border of proximate surfaces, S. G. Perry, 129.
Chain-bearing (editorial), 53.
Change in density of tooth structure, R. R. Andrews, 169.
G. V. Black, 169.
E. C. Kirk, 357.
C. N. Peirce, 168, 360.
W. X. Sudduth, 359.
of address and of printers (editorial), 430.

Changes following pulp devitalization, Wm. H. Trueman, 455.
Chaos, W. H. Atkinson on, 47, 49.
Charcot, J. M., on certain diseases of the nervous system, 118.
Character of power applied in regulating, S. H. Guilford, 399.
Cheap dental appliances, how to secure, W. G. A. Bonwill, 493.
dentures, J. M. Edmunds, 655.
Checking of dry teeth, G. L. Curtis, 86.
Chemical action in caries, Geo. S. Allan, 130.
changes in oral cavity, C. N. Peirce, 333.
composition of dental tissues, C. N. Peirce, 333.
metabolism, Dr. Hunter, 262.
Dr. Vignal, 263.
theory of caries, G. W. McElhaney, 637.
Chemistry of ceramics, W. H. Atkinson, 105.
physiological, W. H. Atkinson, 50.
W. X. Sudduth, 46.
study of, by dentists, W. H. Atkinson, 748.
Chewing-gum, R. C. Newton, 579.
Chicago College of Dental Surgery, commencement of, 301.
Dental Club and the International Journal, 764.
Society, election of officers, 318.
Children's teeth, gutta-percha fillings in, S. B. Palmer, 387.
Chitine, R. R. Andrews, 194.
Chloride of zinc, Jas. Truman, 212.
in tubuli, S. G. Perry, 223, 231.
Chloro-percha, S. B. Palmer, 389.
root-canal fillings, Joseph Head, 278.
Choice of material for root-filling, Wm. H. Trueman, 456.
of patient for implantation, G. L. Curtis, 87.
of teeth for implantation, G. L. Curtis, 86.
Cholera ptomaine, C. C. Barker, 263.
Chorea from dental irritation, A. P. Brubaker, 644.
Christian Science, Cornwall, 100.
G. L. Curtis, 101.
E. C. Kirk, 101.
Chromic acid in pyorrhœa alveolaris, Jas. Truman, 743.
Chronic abscess and implantation, L. C. Bryan, 567.
alveolar abscess, Wm. H. Trueman, 450.
pulpitis, superficial, Jas. Truman, 207.
pyorrhœa alveolaris, M. L. Rhein, 744.
Chupein, Theo. F., a suggestion, 582.
on cotton as root-canal filling, 371.
on the use of natural teeth in crown-work, 582.
Circular from H. A. Smith, 382.
Circulation, how affected, Jas. Truman, 3.
in dentine, 309.
in tooth substance, Geo. S. Allan, 137.
R. R. Andrews, 169.
Circulating system of dentine, C. N. Peirce, 167.
Clamps and pyorrhœa alveolaris, Jas. Truman, 742.
Clasps for narrow plates, E. A. Bogue, 185.
use of, in implantation, G. L. Curtis, 87.
Clean mouths, H. C. Register, 93.
Cleanliness and caries, E. A. Bogue, 438.
L. A. Faught, 355.
Cleavage of the enamel, J. N. Farrar, 268.
Clifford, E. L., a case of difficult eruption of wisdom teeth, 762.
Climax of blunders, the, W. G. A. Bonwill, 16.
Clinic, contour work, T. S. Waters, 107.
with cylinders, H. C. Register, 237.
gold, platinum, and iridium, C. F. Wheeler, 107.
implantation, G. L. Curtis, 108.
E. C. Kirk, 107.
in-lays, W. S. How, 238.
R. W. Starr, 238.
removable bridge-work, T. S. Waters, 237.
use of nerve and crown drills, J. G. Morey, 238.
Clinics, W. H. Atkinson on, 462.
W. G. A. Bonwill on, 84.
Thomas Fillebrown on, 633.
S. B. Palmer on, 386.
J. G. W. Werner on, 471.
college, Weatherby, 472.
New Jersey State Society, 107.
Odontological Society of Pennsylvania, 237.
invisibility of, Thomas Fillebrown, 633.
in regulating, W. G. A. Bonwill on, 84.
raised platforms for, Thomas Fillebrown, 633.
value of, S. B. Palmer, 386.
Clinical Conference of the Massachusetts State Dental Society, Wm. H. Atkinson, 463.
instruction, W. H. Atkinson on, 462.
S. B. Palmer on, 391.
lecture, James Garretson, 348.
medicine, the teeth in diagnosis, A. P. Brubaker, 645.
operations, W. H. Atkinson on, 462.
W. X. Sudduth on, 476.
J. G. W. Werner on, 471.
Closing mouths of tubuli, H. C. Register, 292.
Coagulators, Jas. Truman, 212.
Coaptation, perfect, of gold by Bonwill's method, E. C. Kirk, 523.
Cocaine, Dr. Cornwall, 100.
G. L. Curtis, 101.
W. H. Dwinelle, 98.
Dr. Hugenschmidt, 99.
E. C. Kirk, 99.
C. P. Pruyn, 316.
J. W. Russell, 651.
John Story, 190.
action of, G. L. Curtis, 101.
Dr. Hugenschmidt, 99.
E. C. Kirk, 99.

Cocaine, antidotes, E. C. Kirk, 99, 100.
caution, W. H. Dwinelle, 98.
dangers of self-injection, C. P. Pruyn, 316.
habit, the, C. P. Pruyn, 316.
in implantation, G. L. Curtis, 108.
in pulp-treatment, Jas. Truman, 206.
limit in quantity, W. H. Dwinelle, 99.
E. C. Kirk, 99.
Code of ethics, the, 184.
Codman, J. T., on enforced climate and diet as affecting the teeth, 22.
Coffin-plate, the, W. C. A. Bonwill, 10.
Cohesive foil, when to use, E. B. Davis, 274.
gold, S. G. Perry, 141.
a delusion and a snare, 293.
transition of, from cohesive to soft, Jas. Truman, 276.
Cold compress in inflammation, S. G. Perry, 87.
Collapse and mental emotion, W. X. Sudduth, 32.
or shock, Jas. Truman, 1.
symptoms of, Jas. Truman, 4.
College, the, as a factor in dental education, A. G. Bennett, 589.
clinics, I. J. Weatherby, 472.
commencements, 300.
diploma, the, S. B. Palmer, 385.
lectures, A. G. Bennett, 592.
of Physicians and Surgeons, New York (editorial), 112.
Colleges accepted by the National Association of Dental Examiners, list of, 558.
Color, change of, in implanted teeth, G. L. Curtis, 88.
of gum in pyorrhœa alveolaris, Geo. S. Allan, 715.
of teeth, changes in, C. N. Peirce, 167.
Columbian University, dental department commencement, 303.
Combination fillings, D. M. Clapp, 158.
of devices in bridge-work, T. S. Waters, 199.
Comfortable usefulness, W. H. Trueman, 453.
Commercial journalism, G. A. Gerry, 634.
Committee of Odontological Society of Pennsylvania on treatment of loss of muscular power of jaw, 366.
on colleges, National Association of Dental Examiners, 559.
Comparative biology, C. N. Peirce, 616.
loss of teeth from pyorrhœa alveolaris, Geo. S. Allan, 713, 745.
James Truman, 739.
Compiler, the: why needed, A. G. Bennett, 593.
Complimentary dinner to Dr. Wm. H. Atkinson, 248.
Compressed alveolar borders, W. G. A. Bonwill, 18.
hot air, H. C. Register, 291.
Jas. Truman, 213.
Compression of teeth in their sockets in regulating, E. H. Angle, 327.
Compression of pericementum in regulating, S. H. Guilford, 398.
Condensation of gold foil, Bonwill's method, E. C. Kirk, 521.
Condensed list of short-term colleges, 307.
Condensing lens, S. G. Perry, 148.
Conditions favorable for implantation, G. L. Curtis, 86.
inviting decay, Geo. S. Allan, 130.
of soft tissues in voluntary tooth-movement, S. H. Guilford, 153.
which promote or retard the progress of dental caries, C. N. Peirce, 329.
discussion, 352.
Conemaugh, H. C. Register, 528.
Congestion of ideas, A. G. Bennett, 596.
Conjunctivitis, W. H. Atkinson, 167.
Connection of pulp with tooth-body, Jas. Truman, 203.
Conservation of the dental pulp, Wm. H. Trueman, 456.
Conservatism (editorial), 179.
Constitution and habits as indicated by the teeth, R. C. Newton, 576.
Constitutional effects from copper amalgam, F. A. Cooke, 413.
diathesis theory of pyorrhœa alveolaris, Geo. S. Allan, 717.
Constitutionality of dental laws (editorial), 539.
Contact at grinding surface only, S. G. Perry, 148.
Contamination in rubbber dam, L. A. Faught, 296.
Continuous pressure in regulating, S. H. Guilford, 400.
Contraindications for implantation, G. L. Curtis, 87.
E. C. Kirk, 96.
Contour, the question of, Theo. F. Chupein, 173.
S. G. Perry, 66.
in amalgam fillings, F. S. Bassett, 600.
fillings, J. N. Crouse, 175, 413.
W. H. Dwinelle, 171, 176.
S. H. Guilford, 174.
C. A. Kingsbury, 173.
Dr. Dwinelle's, S. G. Perry, 72, 145.
objections to, W. H. Dwinelle, 172.
work, clinic of, H. C. Register, 107.
T. S. Waters, 107.
C. F. Wheeler, 107.
Contouring corners by inlays, C. Thomas, 317.
reasons for, W. H. Dwinelle, 171.
Contourists, the, Theo. F. Chupein, 173.
Flagg, 173.
Convex points, C. S. Beck, 537.
Convex-shaped pluggers, J. A. Libbey, 538.
Cooper, Dr. (Dublin), on deafness and the wisdom teeth, 642.
Copper amalgam, C. S. Beck, 607.
E. Parmly Brown, 674.
Charles S. Butler, 414.

Copper amalgam, F. A. Cooke, 413.
J. N. Crouse, 414.
G. L. Curtis, 412.
Oswald Fergus, 415.
George A. Maxfield, 416.
S. B. Palmer, 411.
T. H. Parramore, 416.
S. G. Perry, 141, 148.
J. W. Russell, 651.
W. H. Trueman, 675.
S. G. C. Watkins, 673.
I. J. Weatherby, 410, 416.
American *vs.* European, J. W. Russell, 652.
amount used, J. W. Russell, 651.
and acid secretions, J. W. Russell, 653.
Dr. Sanger, 675.
and alloys, J. W. Russell, 654.
and germs, George A. Maxfield, 416.
and gold in combination, J. W. Russell, 654.
and nickel screws, W. H. Trueman, 676.
and the weather, Dr. Gilson, 675.
antiseptic properties of, E. Parmly Brown, 674.
S. B. Palmer, 389.
bright fillings, J. W. Russell, 653.
Dr. Sanger, 674.
crowns, J. W. Russell, 654.
discussion of J. W. Russell's paper on, 673.
in proximate surfaces, S. G. Perry, 141.
leakage, Dr. Sanger, 675.
manipulation of, S. G. C. Watkins, 673.
methods of manufacture, J. W. Russell, 652.
W. H. Trueman, 675.
overheating, S. G. C. Watkins, 673.
quick-setting, S. G. C. Watkins, 673.
shrinkage of, W. H. Trueman, 676.
S. G. C. Watkins, 673.
St. George Elliott on, 652.
the future of, J. W. Russell, 655.
the zinc process, J. W. Russell, 653.
*vs.* copper alloy, Dr. Foote, 674.
weighing fillings, J. W. Russell, 653.
when and where to use, J. W. Russell, 654.
why? George F. Waters, 417.
Cornea, blood in the, W. H. Atkinson, 167.
lymph spaces in, W. H. Atkinson, 167.
Cornwall, Dr., on cocaine, 100.
on implantation, 100.
Coronal arch in bicuspids, S. G. Perry, 143.
Corpuscle, the, W. H. Atkinson, 47, 50.
Corundum points, Northrop forms, W. G. A. Bonwill, 492.
Cosmic dentistry, W. Storer How, 469.
Cosmoline, Joseph Head, 281.
and arsenious acid, A. B. Harrower, 298.
and cotton as root-filling, E. C. Kirk, 293.
as filling material, Jas. Truman, 371.
carbolized, in root-canals, E. C. Kirk, 293.
Cosmoline in root-canals, St. George Elliott, 281.
Jas. Truman, 285.
Cosmos, the, and the Dental Protective Association (editorial), 565.
Cotton as root-canal filling, George S. Allan, 225.
A. C. Boice, 287.
W. G. A. Bonwill, 288.
T. F. Chupein, 371.
J. N. Crouse, 227.
W. H. Dwinelle, 230.
L. Ashley Faught, 225, 286.
C. E. Francis, 227.
Joseph Head, 224, 277, 284.
E. C. Kirk, 292.
D. N. McQuillen, 372.
J. D. Patterson, 286.
H. C. Register, 289.
G. F. Root, 588.
Ambler Tees, 291.
James Truman, 213, 284.
and creosote as root-canal filling, T. F. Chupein, 371.
J. D. Patterson, 286.
W. H. Trueman, 372.
and carbolic acid as a filling material, Joseph Head, 282.
James Truman, 371.
and carbolized cosmoline a water-tight antiseptic filling material, Joseph Head, 370.
and sandarach as root-canal filling, J. D. Patterson, 286.
and sandrach varnish as a covering for arsenious acid, L. A. Faught, 296.
Louis Jack, 296.
D. Maynard, 296.
at apex of root-canal, Ambler Tees, 291.
dressings, advantages of, Joseph Head, 281.
when to use, Joseph Head, 281.
root-canal fillings, objections to, Joseph Head, 277.
Counter-electromotive force, H. E. Roberts, 707.
Counter-irritation, Joseph Head, 280.
Counter-sunk teeth in bridge-work, J. M. Edmunds, 659.
Covering for arsenious acid applications, A. C. Boice, 298.
L. A. Faught, 296.
Louis Jack, 296.
Dr. Maynard, 296.
Cracks as factors in caries, George S. Allan, 129.
in approximal surfaces, R. R. Andrews, 519.
Creative fiat, W. X. Sudduth on, 46, 51.
James Truman, 51.
Credit due to Dr. Younger, G. L. Curtis, 88.
Creolin, Thomas Fillebrown, 163.
Creosote, oil of cloves, and oxide of zinc in pulp-treatment, C. S. Beck, 670.
paste in pulp-treatment, James S. King, 649.

Creosote paste and iodoform in pulp-treatment, James T. King, 651.
Cretins, the teeth of, R. C. Newton, 577.
Crimped foil for Bonwill's method, E. C. Kirk, 521.
Critic, the, A. G. Bennett, 598.
Cross-bar in correcting irregularities, S. E. Gilbert, 373.
Crouse, J. N., and the International Tooth-Crown Company, 254, 571, 764.
on the Arthur method, 175.
on capping pulps, 226.
on carbolic acid, 226.
on contour fillings, 175, 413.
on copper amalgam, 414.
on cotton in root-canals, 227.
funds for defense of, 571.
resolutions of American Dental Association, 571.
suit against, 254, 571, 764.
Crowded arches a factor in caries, George S. Allan, 129.
Crowns and bridge-work, E. B. Davis, 272.
a perfect joint, L. C. Bryan, 61.
artificial, in implantation, G. L. Curtis, 86.
natural, in crown-work, T. F. Chupein, 582.
of copper amalgam, J. W. Russell, 654.
restoration of, W. H. Dwinelle, 172.
Crystal gold, W. H. Dwinelle, 175.
S. G. Perry, 140.
Crystallization of amalgam, W. H. Atkinson, 748.
W. S. Elliott, 730.
Cunningham, George, on treatment of pulpless and abscessed teeth, notice of, 117.
Cure of antral disease without tooth extraction, J. N. Farrar, 457.
Curriculum, our, accepted, E. H. Smith, 321.
the modern, W. H. Atkinson, 460.
Curtis, G. L., clinic, 108.
on absorption, 87.
on Christian Science, 101.
on cocaine, 101.
on copper amalgam, 412.
on the International Tooth-Crown Company, 482.
on the implantation of teeth, 85.
Cushing's instruments for pyorrhœa alveolaris, George S. Allan, 721.
Cuspid and bicuspid separator-bar, J. A. Woodward, 90.
eruption of, S. H. Guilford, 401.
lower, implantation of, B. A. R. Ottolengui, 346.
moving, in regulating, E. H. Angle, 328.
outside of arch, case of self-regulation, S. H. Guilford, 401.
W. X. Sudduth, 418.
superior, precautions in implantation, B. A. R. Ottolengui, 340.
Cutting away the teeth, E. J. Dunning, 74.
Benjamin Lord, 74.
edges, abrasion of, James Truman, 170.
Cylinders at cervical borders, S. H. Guilford, 175.

D.

Data, desired, on implantation, H. A. Smith, 382.
Davidson Rubber Company exhibit, 236.
Davis, E. B., on soft foil and progressive dentistry, 271.
Dead line, the, J. N. Farrar, 270.
pulp under fillings, Wm. H. Trueman, 452.
teeth, caries of, Fr. Abbott, 138.
George S. Allan, 138.
Deafness of dental origin, A. P. Brubaker, 642.
Death from nitrous oxide, W. B. Macleod, 758.
of pulp, causes, James Truman, 206.
progressive stages, James Truman, 206.
Decadence of American Dental Association (editorial), 627.
Decalcification of tooth tissues, C. N. Peirce, 335.
Decay, dental problem of, Geo. S. Allan, 129.
etiology of, E. B. Davis, 272.
prevention of, E. B. Davis, 272.
in enamel and dentine, W. X. Sudduth, 353.
in pits and fissures, G. V. Black, 516.
*vs.* caries, E. C. Kirk, 358.
C. N. Peirce, 360.
W. X. Sudduth, 352, 359.
Deciduous teeth, alveoli of, S. H. Guilford, 395.
copper amalgam for, J. W. Russell, 654.
preservation of, S. A. White, 124.
treatment of, J. Y. Crawford, 127.
Decomposed pulp, chemical relations of, James Truman, 209.
Defective manipulation, S. B. Palmer, 389.
tooth structure, R. R. Andrews, 512.
Deficient culture (editorial), 111.
process tissue in implantation, C. N. Peirce, 91.
Deformed central incisors, case of, L. C. Taylor, 647.
Degrading the dental profession, W. G. A. Bonwill, 493.
Degree of A.M. in dental schools, E. H. Smith, 322.
of M.D. for dentists, E. H. Smith, 320.
De Lange on implantation, 97.
Demand and supply, G. L. Curtis, 85.
in dental literature, A. G. Bennett, 595.
Density of tooth structure, James Truman, 356.
changes in, R. R. Andrews, 169.
G. V. Black, 169.
E. C. Kirk, 357.
C. N. Peirce, 168, 360.
W. X. Sudduth, 359.
Dental advertising in 1786, 380.

Dental appliances, how to cheapen, W. G. A. Bonwill, 493.
art, nature of, A. G. Bennett, 593.
progress of, C. J. Essig, 164.
artists, J. M. Edmunds, 655.
assertion and discussion, Joseph Head, 524.
discussion of, 533.
bibliography, George Crowley, 469.
of Cosmos, W. S. How, 469.
caries, etiology of, George S. Allan, 129.
C. N. Peirce, 168.
predisposing causes, George S. Allan, 129.
W. C. Wardlaw, 701.
problem of, George S. Allan, 129.
progress of, C. N. Peirce, 329.
and micro-organisms, C. C. Baker, 264.
club, a, W. X. Sudduth, 470.
J. G. W. Werner, 471.
colleges of Chicago (editorial), 764.
commencements, 300.
Congress, International, American Medical Association on, 503.
E. Brasseur, 377.
editorial, 374.
George P. Kuhn, 245.
P. Du Bois, 187.
W. H. H. Thackston, 245.
decay, problem of, George S. Allan, 129.
Department, Columbian University, commencement of, 303.
Harvard University, 303.
Southern Medical College, 304.
St. Louis College of Physicians and Surgeons, 304.
University of Iowa, 303.
of Tennessee, 204.
education, Eugene H. Smith, 319.
C. J. Essig on, 165.
L. C. Ingersoll on, 191.
W. H. Morgan on, 192.
J. Taft on, 192.
B. Holly Smith on, 190.
in medical colleges, E. H. Smith, 322.
engine, the, W. H. Dwinelle, 172.
W. S. Elliott, 729.
S. H. Guilford, 175.
exhibits, Ash & Son, 236.
Blair, H. C., & Sons, 236.
Davidson Rubber Company, 236.
Florence Manufacturing Company, 108.
Green, W. P., 237.
Justi, H. D., 237.
Keller Medicine Company, 236.
Mulford & Co., 237.
New Jersey State Dental Society, 108.
Odontological Society of Pennsylvania, 236.
Parke, Davis & Co., 236.
Rowan, Ed., 237.
Seabury & Johnson, 108, 236.
See, A. W., & Co., 108.
Dental exhibits, Sibley, Gideon, 108, 236.
Speakman, W. M., 237.
Welch & Co., 108.
White, S. S., & Co., 108, 236.
Williams, R. S., 236.
Wilmington Manufacturing Company, 236.
aluminum apparatus, C. C. Carroll, 237.
amalgam instruments, S. C. G. Watkins, 108.
antiseptic material, Seabury & Johnson, 108, 236.
benefits of, George W. Lovejoy, 405.
bone food, Parke, Davis & Co., 236.
crystalloid plastic gold, R. S. Williams, 236.
dentifrices, H. K. Mulford, 237.
editorial, 244.
electric appliances, S. S. White, 236.
hot-air apparatus, Parson's, 237.
electro-metallic plates, Ward, 108.
deposit plates, C. A. Timme, 107.
felt gold, Gideon Sibley, 236.
forceps, new shapes, V. C. Pond, 158.
Genese's specialties, 236.
gum cutter, Loveland, 161.
hydronaphthol specialties, Seabury & Johnson, 236.
McLean disks, 158.
mirror-frame, C. P. Wilson, 162.
napkin-holder, Hopkins, 162.
new instruments and methods, American Academy of Dental Science, 158.
rapid excavator, A. G. Bennett, 238.
Register's engine and hand-piece, 236.
rubber-dam clamp, Ivory, 108.
saw-frame, C. P. Wilson, 162.
separator, J. G. Morey, 238.
H. A. Parr, 238.
tooth-brushes, Florence Manufacturing Company, 108.
W. W. Walker on, 762.
graduates (editorial), 300.
histology, R. R. Andrews, 166.
History, No. 6, J. Hayhurst, 102.
A. G. Bennett, 595.
home, a, W. H. Atkinson, 462.
George W. Lovejoy, 473.
W. X. Sudduth, 470.
hospitals, I. J. Weatherby, 472.
J. G. W. Werner, 471.
implantation, data desired, H. A. Smith, 382.
inlays, clinic of W. S. How, R. W. Starr, 238.
irregularities, E. H. Angle, 323.
irritation and eye and ear disorders, A. P. Brubaker, 643.

Dental irritation, reflex effects of, A. P. Brubaker, 639.
journal as a commercial publication, G. A. Gerry, 634.
its mission, A. G. Bennett, 591.
journalism (editorial), 183.
law of Minnesota, 314.
legislation, Dr. Dean on, A. G. Bennett, 592.
literature, A. G. Bennett, 588.
B. Holly Smith, 190.
materia medica, James Truman, 209.
medical practitioner, E. H. Smith, 319.
nerve, inferior, excision of, Thomas Fillebrown, 215.
operations, prolonged, Jas. Truman, 5.
shock in relation to, James Truman, 1.
patients, L. C. Bryan, 246.
pharmaceutical preparations, James Truman, 204.
prosthesis, J. M. Edmunds, 655.
Protective Association, dues of, 765.
editorial, 182.
J. N. Crouse, 376, 477.
L. Ottofy, 318.
C. N. Peirce, 750.
W. X. Sudduth, 53, 751.
and the Cosmos (editorial), 687.
and the International Journal, 687.
membership of, J. N. Crouse, 482.
Brooklyn Dental Society on, 765.
New Jersey State Society on, 750.
Reform Society, Frank Harrison, 409.
Section of Tenth International Medical Congress (editorial), 431.
society, the, a factor in education, A. G. Bennett, 590.
societies, object of, C. J. Essig, 165.
surgery, advances in, R. C. Newton, 576.
tissues, chemical composition of, C. N. Peirce, 833.
sensibility of, C. N. Peirce, 167.
Vulcanite Company, suits against, H. C. Merriam, 485.
L. D. Shepard, 483.
I. J. Weatherby, 483.
"Dental Medicine," by F. J. S. Gorgas, notice of, 435.
"Dental Science," by L. C. Ingersoll, notice of, 433.
Dentinal canaliculi, Fr. Abbott, 133.
fibriles, 309.
septic influence of, in implantation, G. L. Curtis, 88.
tubuli, S. G. Perry, 222.
C. N. Peirce, 231.
James Truman, 212.
and oxychloride of zinc, J. D. Patterson, 286.
condition of, after pulp putrefaction, H. C. Register, 290.
desiccating, A. G. Bennett, 289.
treatment of, James Truman, 285.
Dentine, carious, micro-organisms in, George S. Allan, 131.
change in density of, R. R. Andrews, 169.
G. V. Black, 169.
E. C. Kirk, 357.
C. N. Peirce, 168, 360.
W. X. Sudduth, 359.
character of, S. B. Palmer, 388.
in devitalized teeth, Wm. H. Trueman, 452.
circulatory system of, R. R. Andrews, 169.
C. N. Peirce, 167.
decalcification, C. N. Peirce, 332.
desiccation of, H. C. Register, 291.
elasticity of, J. Morgan Howe, 697.
formation of, R. R. Andrews, 193.
germ, C. N. Peirce, 331.
injected red, W. H. Dwinelle, 169.
matrix, C. N. Peirce, 331.
organic matter of, H. C. Register, 290.
permeability of, C. N. Peirce, 167.
recalcified, W. H. Dwinelle, 229.
relation of pulp to, James Truman, 206.
reparatory changes in, R. R. Andrews, 169.
secondary, George S. Allan, 170.
deposition of, T. H. Parramore, 393.
senile, James Truman, 170.
structural defects of, C. N. Peirce, 331.
structure of, C. N. Peirce, 331.
translucent tubules in, James Truman, 170.
when formed, James Truman, 170.
Dentistry, a progressive profession, E. B. Davis, 272.
a science of demonstration, W. S. Elliott, 726.
an independent science, L. P. Ingersoll, 191.
and medicine, relations of, J. Taft, 191.
benefits conferred by, R. C. Newton, 582.
dignified position of, R. C. Newton, 578.
in America (editorial), Dr. Kuhn, 117.
not a specialty of medicine, L. P. Ingersoll, 191.
relation of, to medicine, C. E. Francis, 59.
Dentists' act, Frank Harrison, 409.
register, Frank Harrison, 409.
Dentists of Johnstown, Pa., and the Conemaugh disaster, H. C. Register, 529.
three grades of, S. B. Palmer, 384.
Department of Dental Surgery, University of Maryland, commencement of, 303.
of dentistry, Vanderbilt University, commencement of, 304.
Deposition of lime salts, W. X. Sudduth, 356.
James Truman, 304.
of secondary dentine, T. H. Parramore, 393.
Deposits, removal of, from teeth, Wm. N. Morrison, 190.

Deposits upon the teeth, character of, C. N. Peirce, 334.
Dermal appendages, development of, W. X. Sudduth, 548.
Deroutation of nerve currents, A. P. Brubaker, 645.
Description of process for stamping plates by hydraulic press, E. A. Bogue, 391.
Destruction of ear structures due to dental irritation, A. P. Brubaker, 642.
of process from arsenious acid, F. S. Bassett, 299.
of tissues by arsenious acid, L. A. Faught, 297.
Determining safe length of root in implantation, B. A. R. Ottolengui, 339.
Developing dentine, odontoblastic layer of, W. X. Sudduth, 544.
Development of dentistry, history of, W. Storer How, 466.
of the tooth, C. N. Peirce, 330.
as test of age, R. C. Newton, 581.
Devitalization of pulps, G. F. Root, 584.
Devitalized deciduous teeth, J. Y. Crawford, 127.
dentine over exposed pulp, C. S. Beck, 671.
teeth, reliability of, Wm. H. Trueman, 453.
pulp, removal of, G. F. Root, 585.
Diabetes and nitrous oxide, Dr. Thomas, 34.
A. N. Peirce, 9.
James Truman, 9.
Diagnosis in pulp preservation, James S. King, 650.
of arsenical ulcers, L. A. Faught, 297.
of serumic deposits, Geo. S. Allan, 718.
the teeth as a factor in, R. C. Newton, 575.
Diagnostic point, arsenious acid, L. A. Faught, 297.
skill, W. S. Elliott, 727.
Diamond drill, the, W. G. A Bonwill, 492.
in removing stone from the bladder, W. G. A. Bonwill, 495.
Dickey, S. J., on arsenious acid, 298.
"Die Microorganismen der Mundhöhle," by W. D. Miller, notice of, 691.
Diet, Dr. Bailey, 41.
W. G. A. Bonwill, 18.
Dr. Brown, 43.
J. T. Codman, 45.
C. S. Stockton, 39.
Dr. Thayer, 38.
James Truman, 39.
R. B. Winder, 41.
and character, Dr. Bailey, 42.
J. T. Codman, 30.
and dentition, W. G. A. Bonwill, 18.
and strength, J. T. Codman, 45.
and the teeth, James Truman, 39.
R. B. Winder, 41.
of New Englanders, J. T. Codman, 44.
C. S. Stockton, 39.
Difference of potential definition, H. E. Roberts, 705.
Differences of opinion, S. G. Perry, 67.
Differential diagnosis, arsenical and rodent ulcers, L. A. Faught, 297.
Difficult impressions, Sid. Holland, 125.
Digestive ferments, W. X. Sudduth, 354.
Dignity, a factor in character, S. B. Palmer, 385.
Dinner, complimentary, to Dr. Wm. H. Atkinson, 249.
of New York Odontological Society, 250.
Diploma, the, what it represents (editorial), 308.
Diploma-Mills, check to, J. H. Martindale, 570.
Discretion, J. W. Russell, 652.
Wm. H. Trueman, 675.
Discussion of Dr. Allan's paper on "Dental Caries," 166.
on "Pyorrhœa Alveolaris," 739.
Dr. Atkinson's paper on "Thoroughness," 470.
Dr. Bassett's paper on "The Use of the Matrix with Proximal Amalgam Fillings," 602.
Dr. Bogue's paper on "Making Dies and Casts and a Process of Swedging," 420.
Dr. Bonwill's paper on "Patents," 495.
Dr. Brubaker's paper on "Reflex Effects of Dental Irritation," 662.
Dr. Codman's paper on "Enforced Climate and Diet as Affecting the Teeth," 37, 43.
Dr. Crouse's remarks on "The Dental Protective Association," 477.
Dr. Curtis's paper on "Implantation," 94.
Dr. Edmund's paper on "Removable Bridge-work," 677.
Dr. Elliott's paper on "Light to the Gentiles," 747.
Dr. Faught's paper on "A Study of Arsenious Acid," 298.
Dr. Guilford's paper on "Physiology of Tooth Movement in Regulating," 418.
on "Voluntary Tooth Movement Resulting in Excessive Interdental Space," 176.
Dr. Hayhurst's paper on "Dental History," 104.
Dr. Head's paper on "Dental Assertion and Discussion," 533.
Dr. Head's paper on "The Advantages of Cotton as a Root-Canal Filling," 284.
Dr. King's paper on "Treatment of Exposed Tooth-Pulp," 668.
Dr. Kirk's paper on "The Bonwill Method of Packing Gold Foil," 536.
Dr. Newton's paper on "The Teeth as a Factor in Diagnosis," 611.
Dr. Ottolengui's paper on "Implantation Surgically Considered," 367.
Dr. Palmer's paper on "Elements of Success in Dental Practice," 410.
Dr. Parramore's paper on "Sterilized Sponge for Pulp Capping," 473.

Discussion of Dr. Peirce's paper on "Conditions which promote or Retard the Progress of Dental Caries," 352.
Dr. Perry's paper on "The Treatment of Proximate Surfaces," 171.
Dr. Robert's paper on "A Study of Electricity with a View of Comprehending its Application in Dentistry," 734.
Dr. Root's paper on "The Preparation and Filling of Root-Canals," 608.
Dr. Russell's paper on "Copper Amalgam," 673.
Dr. Sudduth's paper on "The Individuality of the Biological Cell," 551, 555.
Dr. Truman's paper on "Shock in Relation to Dental Operations," 32.
on "The Pulp and Treatment of Pulp Canals," 222.
Dr. Waters's paper on "Removable Bridge-Work," 232.
Dr. White's paper on "The Young Practitioner," 661.
of incidents of practice, 91, 372.
of lantern exhibits, 46, 166, 551, 555.
of topics:
amalgam, 606.
antiseptics, 93.
antral diseases, 422.
arsenious acid, 298.
assertion and proof, 533.
Bonwill method of packing gold, 536.
bridge-work, 232, 677.
caries, 166, 352.
cells, 46, 551.
changes in tooth density, 356.
climate and diet, 37, 43.
cocaine, 98.
copper amalgam, 410, 673.
corpuscles, 46.
cosmoline, 370, 610.
cotton root-canal fillings, 224, 284, 370.
dental clubs, 470.
dental history, 102, 104.
Dental Protective Association, 482, 750.
diet, 37, 43.
electricity, 734.
elements of success, 410.
excision of dental nerves, 218.
exposed pulps, 668.
food, 39.
hemorrhage, 367.
implantation, 94, 367, 421.
matrices, 602.
mouth breathing, 419, 611, 616.
packing gold foil, 536.
patents, 495.
perforation of antrum, 421.
porcelain teeth, 104.
proximal fillings, 171, 602.
pulp capping, 226, 473, 669.
Discussion of topics:
pyorrhœa alveolaris, 176,739.
reflex effects of dental irritation, 662.
root canals, 222, 284, 369, 608.
shock in relation to dental operations, 32.
sterilized sponge in pulp capping, 473.
swedging plates, 420.
syphilitic teeth, 613.
the cell doctrine, 46, 551, 555.
the pulp, 222.
the teeth in diagnosis, 611.
thoroughness, 470.
tooth movement in regulating, 418.
tooth movement from calculus, 176.
treatment of proximate surfaces, 171.
treatment of root canals, 223, 288, 608.
voluntary tooth movements, 176.
young practitioners, 661.
Discussions, value of, S. B. Palmer, 386.
"Diseases of the Nervous System," by J. M. Charcot, M.D., notice of, 118.
Diseases of childhood, effect of, upon dentine, C. N. Peirce, 332.
of the antrum, spray syringe for, J. N. Farrar, 457.
of the ear and the teeth, R. C. Newton, 579.
C. N. Peirce, 616.
of the nervous system,
of women, study of, by dental students, E. H. Smith, 321.
originating in pathological conditions of the teeth, A. P. Brubaker, 640.
Disinfection of decalcified dentine, C. S. Beck, 671.
of instruments, B. A. R. Ottolengui, 337.
Disinfectants in implantation, E. C. Kirk, 367.
B. A. R. Ottolengui, 337.
Dr. Weld, 337.
Disintegrated dentine, G. W. Chump, 668.
James S. King, 668.
Disintegration of cement, J. N. Farrar, 270.
Dissecting-rooms, teeth from, for implantation, Dr. Wetzel, 246.
Distended tubules in artificial caries, Geo. S. Allan, 132.
in caries, Geo. S. Allan, 138.
Diverted nutrition, C. N. Peirce, 616.
Dixon, C. M., on arsenious acid, 298.
Domestic correspondence:
Geo. S. Allan, 253.
Ed. H. Angle, 379.
E. A. Bogue, 186, 312, 438.
E. C. Briggs, 632.
E. L. Clifford, 761.
E. T. Darby, 631.

Domestic correspondence:
J. N. Farrar, 380.
L. A. Faught, 313.
Thomas Fillebrown, 633.
G. A. Gerry, 634.
F. J. S. Gorgas, 120.
J. Morgan Howe, 697.
M. G. Jenison, 570.
H. E. Keely, 509.
E. C. Kirk, 699.
F. A. Levy, 60, 121.
J. Bond Littig, 698.
W. E. Magill, 635.
John S. Marshall, 695.
J. H. Martindale, 570.
H. C. Register, 251.
M. L. Rhein, 380.
Wm. H. Rollins, 186.
Ambler Tees, 119.
F. T. Van Woert, 438.
W. W. Walker, 762.
George F. Waters, 313.
dog, bad breath in, S. C. G. Watkins, 42.
caries of teeth in, W. D. Miller, 244.
pyorrhœa alveolaris in, a case of, B. A. R. Ottolengui, 43.
tartar on teeth of, S. C. G. Watkins, 42.
teeth of, B. A. R. Ottolengui, 43.
W. X. Sudduth, 42.
S. C. G. Watkins, 42.
horse, caries in teeth of, W. D. Miller, 244.
Donaldson's nerve broaches, G. F. Root, 585.
Donation to the profession, W. G. A. Bonwill, 492.
T. S. Waters, 200.
Drilling for implantation, E. C. Kirk, 368.
B. A. R. Ottolengui, 338.
root-canals, W. H. Morgan, 126.
Drugs, acquaintance with, L. A. Faught, 293.
misuse of, L. A. Faught, 293.
Dry cotton as covering for arsenious acid, C. M. Dixon, 299.
teeth, checking of, G. L. Curtis, 86.
tooth in natural socket, implantation of, L. C. Bryan, 568.
Drysdale's definition of a cell, 551.
DuBois's "Aide Mémoire du Chirurgien-Dentiste," notice of, 691.
Ducts, closing mouths of, W. H. Dwinelle, 172.
Dunning, E. T., on cutting away the teeth, 74.
Durability of cement, A. H. Thompson, 439.
Dwinelle, W. H., and contour fillings, S. G. Perry, 72, 145.
and crystal gold, S. G. Perry, 145.
on cocaine, 98.
on implantation, 98.
Dynamo, the, H. E. Roberts, 708.
Dyspepsia, Frank Woodbury, 690.

E.

Ear-diseases and the teeth, R. C. Newton, 579.
C. N. Peirce, 616.
Early morning operations best: why? Jos. Head, 282.
Easy methods, S. G. Perry, 76.
Ebb-tide of blood currents, Jos. Head, 282.
Eclecticism, C. C. Carroll, 685.
Economizing time, W. X. Sudduth, 110.
"Editor and Bus. Manager," 432.
Edmunds, J. Marion, on extensive removable bridge-work, 655.
on æsthetic prosthesis, 655.
on loose teeth as supports in bridge-work, 678.
on shaping capped teeth in bridge-work, 680.
Education, fundamental principles of, L. D. Shepard, 412.
of patients, J. N. Crouse, 413.
J. C. White, 661.
three factors in, A. G. Bennett, 589.
Edwards, H. H., letter from, 436.
Effects of deposits upon the gums, George S. Allan, 717.
upon the teeth, C. N. Peirce, 335.
Elastic materials in regulating, S. H. Guilford, 400.
Electric mallet, the, W. B. Miller, 538.
right-angle plugger for, W. G. A. Bonwill, 490.
motor, the, H. E. Roberts, 710.
plugger-points for gutta-percha fillings, L. A. Faught, 735.
street-car current for dental uses, L. A. Faught, 738.
Electricity, comparison with stream of water, H. E. Roberts, 703.
for dental uses, danger of, W. E. Magill, 736.
H. E. Roberts, 736.
Electro-chemical theory, S. B. Palmer, 388.
magnetic mallet, Bonwill's, E. C. Kirk, 521.
Electrolysis, D. F. McGraw, 316.
Elements of success in dental practice, S. B. Palmer, 383.
J. N. Crouse, 413.
the study of, W. H. Atkinson, 748.
Elevation of college standards, A. G. Bennett, 589.
of dentistry, E. H. Smith, 323.
Elliott, W. S., on antiseptics, 727.
on campho-phenique, 727.
on crystallization of amalgam, 730.
on dentistry as a science, 726.
on diagnostic skill, 727.
on Light to the Gentiles, 726.
on sterilized sponge as pulp-capping, 475.
on the suspension engine, 729.
Elongation of teeth in regulating, E. H. Angle, 327.
Embryology (editorial), 240.
Embryonal bodies, Fr. Abbott, 136.

Embryonic life, tissues in photo-micrographs, W. X. Sudduth, 46.
Emersonian simplicity *vs.* Addisonian elegance, A. G. Bennett, 594.
Emigrants, changed diet of, and the teeth, J. T. Codman, 44.
C. S. Stockton, 43.
Emma Prewitt, Miss, 253.
Empiricism, W. H. Atkinson, 461.
S. B. Palmer, 388.
James Truman, 204.
Enamel and protoplasm, Geo. S. Allan, 136.
arterial connection of, C. N. Peirce, 331.
cap, interruption of continuity of, R. R. Andrews, 511.
cells, functional activity of, C. N. Peirce, 330.
nervous connection of, C. N. Peirce, 331.
prisms, morphology of, C. N. Peirce, 330.
rim of bicuspids, S. G. Perry, 143.
shrinkage of, from dentine in dry teeth, L. C. Bryan, 569.
structural defects of, C. N. Peirce, 331.
tissue normality dependent on? C. N. Peirce, 331.
vices of conformation, C. N. Peirce, 331.
Encapsulation in implantation, E. C. Kirk, 95.
W. X. Sudduth, 97.
Encroachment of secondary dentine, Wm. H. Trueman, 449.
Encourage the young men, B. H. Catching, 702.
L. A. Faught, 662.
J. C. Green, 661.
Sid Holland, 702.
J. C. White, 660.
S. A. White, 701.
Endowments (editorial), 111.
Enforced climate and diet as affecting the teeth, J. T. Codman, 22.
Engine-mallet, Bonwill's, E C. Kirk, 521.
English forceps, position of pins in, V. C. Pond, 159.
Enlargement of the arch, S. H. Guilford, 403.
Enlarging root-canals, G. F. Root, 587.
Entering proximate surfaces, S. G. Perry, 139.
Enthusiasm, S. B. Palmer, 384.
Environment, W. X. Sudduth, 46.
and the development of organs, W. X. Sudduth, 550.
of exposed pulps, J. N. Farrar, 271.
Epiblast, development of, W. X. Sudduth, 547.
microscopic appearance of, W. X. Sudduth, 548.
Epilepsy of dental origin, A. P. Brubaker, 644.
Epithelial tissue, transplantation of, W. X. Sudduth, 548.
Erosion, E. C. Kirk, 358.
Erosion, W. X. Sudduth, 353.
James Truman, 356.
Escharotics, James Truman, 214.
in pulp devitalization, G. F. Root, 584.
Essentials in contour work, S. H. Guilford, 174.
Essig, C. J., address of welcome, 164.
Ether-spray in implantation, B. A. R. Ottolengui, 338.
Etiology of decay, E. B. Davis, 272.
of dental caries, George S. Allan, 129.
C. N. Peirce, 168.
of pyorrhœa alveolaris, George S. Allan, 715.
of voluntary tooth-movement, S. H. Guilford, 150.
Eugenol, penetration of, in dry teeth, L. C. Bryan, 569.
qualities of, H. H. Johnston, 127.
Eureka embroidery silk for finer ligatures, Thomas Fillebrown, 633.
Evolution of mammalian dentition, Prof. E. D. Cope, 688.
Exaggeration of the shapes of the teeth, S. G. Perry, 67.
Excavating, philosophy of, J. N. Farrar, 268.
Excessive charges, J. C. White, 658.
malleting, S. B. Palmer, 389.
stretching of pulp in regulating, S. H. Guilford, 397.
Excision of inferior dental nerve, C. A. Brackett, 219.
Thos. Fillebrown, 215.
Dr. Williams, 221.
Exciting causes of decay, 221.
Exfoliation from arsenious acid, O. E. Inglis, 299.
Exostosed roots and neuralgic headaches, A. P. Brubaker, 644.
Exostosis and aural disease, E. C. Kirk, 664.
and eye-troubles, C. S. Beck, 665.
Expanding the arch, E. H. Angle, 326.
Expansion of gutta-percha fillings, S. G. Perry, 143.
Experiments of F. J. Bennett, R. R. Andrews, 196.
of Prof. Harting and Mr. Rainie, R. R. Andrews, 193.
of Brown-Séquard (editorial), 623.
Experience "ready-made," A. G. Bennett, 590.
Experimental proof of assertions, Joseph Head, 525.
Expert work of R. R. Andrews, George S. Allan, 132.
Explanation, an, 441.
Exploring needle in dentition, W. G. A. Bonwill, 10, 16.
Exposed pulps, J. N. Farrar, 271.
James S. King, 672.
B. A. R. Ottolengui, 340.
T. H. Parramore, 393.
antiseptics in treatment of, T. H. Parramore, 393.
capping, C. S. Beck, 672.
J. N. Crouse, 226.

Exposed pulps, capping, S. J. Dickey, 298.
W. H. Dwinelle, 229.
C. E. Francis, 227.
S. H. Guilford, 473.
James S. King, 649, 669.
W. D. Miller, 671.
T. H. Parramore, 393, 475.
James Truman, 204, 231.
I. J. Weatherby, 476.
environments of, J. N. Farrar, 271.
in lateral incisors, B. A. R. Ottolengui, 340.
painless protection of, J. N. Farrar, 271.
treatment of, James S. King, 649.
walls of cavity, J. N. Farrar, 270.
Extension of college terms (editorial), 562.
Extensive removable bridge-work, J. Marion Edmunds, 655.
External characteristics of teeth with interglobular spaces, R. R. Andrews, 517.
Extracted teeth, preservation of, Theo. F. Chupein, 583.
Extraction, James Truman, 177.
for regulation, S. H. Guilford, 157.
of first permanent molars, W. G. A. Bonwill, 84.
of posterior teeth and interdental spaces, S. H. Guilford, 153, 156.
of second molars, stiffening of jaw from, 366.
shock from, James Truman, 6.
Exudations in pyorrhœa alveolaris, Geo. S. Allan, 715, 717.

F.

Facial muscles, contraction of, from dental irritation, A. P. Brubaker, 643.
neuralgia, C. N. Peirce, 615.
Facilities for repair of bridge-work, T. S. Waters, 199.
Factors in æsthetic prosthesis, J. M. Edmunds, 656.
in dental caries, Geo. S. Allan, 129.
C. N. Peirce, 831.
in dental education, A. G. Bennett, 589.
in voluntary tooth movement, S. H. Guilford, 153, 155.
promoting tooth destruction, George S. Allan, 129.
Facts from experience in use of arsenious acid, L. A. Faught, 294.
Fads in dentistry, J. N. Russell, 651.
Failure, causes of, in implantation, G. L. Curtis, 85.
in contour filling, S. G. Perry, 69.
law of, S. B. Palmer, 388.
False facts, H. C. Register, 529.
Farrar, J. N., spray syringe for treatment of diseases of the antrum, 457.
retaining plugs used in regulating, 264.
Fat embolism in shock, James Truman, 3.
Fatal termination in administration of nitrous oxide gas, W. B. Macleod, 728.
Faught, L. Ashley, on arsenious acid, 293.
on Dr. Bonwill's right-angle plugger, 495.
on Dr. Brubaker's paper on "Reflex Effects of Dental Irritation," 662.
on Dr. Head's paper on "Dental Assertion and Discussion," 534.
on protracted operations, 34.
on root-canal filling, 286.
on sunshine, 35.
Faulty development a factor in dental caries, George S. Allan, 129.
Favorable conditions for implantation, G. L. Curtis, 86.
Fear-allaying in young patients, A. G. Bennett, 36.
Feldspar, test of, Dr. Fowler, 105.
Felted tin (editorial), W. C. B., 242.
Fermentation, its cause and effects, Prof. Laplace, 688.
Ferments, H. C. Register, 531.
Fibres of pericementum, characteristics of, S. H. Guilford, 397.
Fibrillæ, the, H. C. Register, 531.
Fifth nerve, connections of, A. P. Brubaker, 640.
File and binder for dental journals, L. C. Bryan, 760.
Filing teeth, S. G. Perry, 67.
Fillebrown's method of excision of inferior dental nerve, 215.
of packing gold, H. E. Roberts, 538.
"Operative Dentistry," notice of, 434.
Filling pulpless teeth, H. C. Register, 289.
object in, S. B. Palmer, 387.
the ideal, S. G. Perry, 139.
root-canals, A. G. Bennet, 289.
A. C. Boice, 287.
W. G. A. Bonwill, 287.
T. F. Chupein, 371.
G. L. Curtis, 86.
L. A. Faught, 286.
Jos. Head, 277, 369, 525.
Sid. Holland, 127.
H. H. Johnson, 127.
D. N. McQuillen, 372.
W. B. Miller, 609.
W. H. Morgan, 126.
B. A. R. Ottolengui, 372.
J. D. Patterson, 286.
H. C. Register, 289.
G. F. Root, 584.
Ambler Tees, 291.
James Truman, 284.
in implantation, G. L. Curtis, 86.
the object in, Alonzo C. Boice, 287.
Filo-bacteria, C. C. Barker, 258.
Finding his level, S. B. Palmer, 384.
Fine chemicals and drugs, Merck's index, 432.
Fine silk for ligatures, Thomas Fillebrown, 633.

Finishing amalgam fillings, F. S. Bassett, 601.
A. C. Boice, 605.
J. R. C. Ward, 605.
Finney, Wm. B., clinic, 107.
Fir balsam, J. N. Farrar, 268.
First dentition in hereditary syphilis, R. C. Newton, 577.
in rickets, R. C. Newton, 577.
First District Dental Society, New York, the phonograph at, 754.
First molars, extraction of, W. G. A. Bonwill, 84.
James Truman, 177.
Fusion of protozoa, W. X. Sudduth, 542, 545.
Fissures as factors in caries, Geo. S. Allan, 129.
of soft palate, operation for, James E. Garretson, 348.
Fixed bridges, W. H. Dwinelle, 232.
J. M. Edmunds, 679.
Dr. Pinney, 680.
W. L. Rhein, 683.
C. S. Stockton, 679.
T. S. Waters, 197, 682.
caps in regulating, E. H. Angle, 326.
Flagg's standard alloy, A. C. Boice, 606.
James S. King, 606.
J. R. C. Ward, 605.
Florence Manufacturing Company's exhibit, 108.
Fluids, absorption of, by cotton root-fillings, Jos. Head, 297.
of mouth-washing, action on proximate surfaces, S. G. Perry, 139.
Fogg, J. S., on galvanic currents, 366.
Food, Wm. H. Atkinson, 37.
C. S. Stockton, 39.
W. J. Thayer, 38.
James Truman, 39.
for bacteria, Geo. S. Allan, 130.
Foramina of palatal canals in young subjects, B. A. R. Ottolengui, 339.
Force of nature in regulating, S. H. Guilford, 401.
H. A. Baker, 418.
Forceful extirpation of pulp, Jas. Truman, 205, 214.
Forceps, a factor in irregularities, W. G. A. Bonwill, 17.
Pond's view, V. C. Pond, 158.
Foreign correspondence:
E. Brasseur, 377.
L. C. Bryan, 61, 189, 246, 567, 694, 760.
Al. Burne, 629.
Geo. Cunningham, 507.
P. Dubois, 187.
H. H. Edwards, 436, 693.
W. S. G. Elliott, 124.
C. R. Fletcher, 124.
W. Harrison, 247.
G. P. E. Kuhn, 245.
W. B. Macleod, 61, 759.
W. D. Miller, 310.
J. C. Woodburn, 124.
Brazil: Sáo Paulo, 124.
Foreign correspondence:
England: Brighton, 247, 628.
Cambridge, 507.
London, 124.
France: Paris, 187, 245, 377.
Germany: Berlin, 312.
New South Wales: Sidney, 629.
Scotland: Edinburgh, 61, 758.
Glasgow, 124.
Spain: Madrid, 436, 693.
Switzerland: Basel, 61, 189, 246, 567, 694, 760.
Foreigners, teeth of, J. T. Codman, 44.
Form, the meaning of, A. G. Bennett, 593.
Formation of calco-globulin, R. R. Andrews, 193.
of dentine, R. R. Andrews, 193.
Forming socket for implantation, G. L. Curtis, 87.
Formula for arsenious acid, L. A. Faught, 294.
for root-canal filling paste, Van Woert, 372.
for Wood's fusible alloy, 392.
Foster's moulds, W. H. Atkinson, 105.
Fox (1803) on defective tooth structure and its causes, 512.
on enamel fissures, 511.
Geo. H., "Photographic Illustrations of Skin-Diseases," notice of, 118.
Frail cavity walls, safety for, in Bonwill's method, E. C. Kirk, 523.
Fraternal duties, 184.
instinct in the dental profession, C. J. Essig, 164.
privileges, 184.
Free margins in proximate cavities, S. G. Perry, 140, 149.
scholarships (editorial), 114.
French Dental Congress, E. Brasseur, 377.
editorial, 374, 500.
Geo. P. E. Kuhn, 245.
New Jersey State Dental Society on, 503.
Pennsylvania State Dental Society on, 503.
Southern Dental Journal on, 506.
Freshly-extracted teeth in implantation—why? G. L. Curtis, 86.
Fruit-eater, man normally a, J. T. Codman, 22.
Frontispiece, March, dental caries, facing page 129.
April, borderland of calcification, facing page 193.
September, pits and fissures of the enamel, facing page 511.
Full upper dentures in removable bridge-work, J. M. Edmunds, 657.
Function of bacteria, C. N. Peirce, 168.
of microbes, G. V. Black, 317.
of osteoblasts, S. H. Guilford, 397.
of peridental membrane, S. H. Guilford, 397.
of a plate, T. S. Waters, 198.

Functional activity of enamel cells, C. N. Peirce, 330.
diseases of the eye caused by the teeth, A. P. Brubaker, 641.
Future of dentistry, the, C. N. Peirce, 168.
success of the profession, J. C. White, 661.

G.

Galezowski on ocular disease and the teeth, A. P. Brubaker, 640.
Galvanic battery, the, H. E. Roberts, 705.
current in loss of muscular power in jaw, 366.
Garretson, James E. clinical lecture, 348.
Gas-bubbles in tubuli, R. R. Andrews, 518.
narcosis, W. B. Macleod, 758.
General anatomy of the dental pulp, Wm. H. Trueman, 455.
Generation of electricity, H. E. Roberts, 705.
Geometrical law of articulation, W. G. A. Bonwill, 84.
German silver for bands in regulating, E. H. Angle, 324.
for matrices, F. S. Bassett, 601.
Germ theory, the, Geo. S. Allan, 130, 138.
W. C. Browne, 637.
H. C. Register, 529.
W. C. Wardlaw, 637.
editorial, 179.
of disease, James Truman, 209.
Germs, harbor for, in leaking root-fillings, Jos. Head, 369.
putrefactive, Jos. Head, 279.
Germicides in dental practice, T. H. Paramore, 393.
in root-canal treatment, H. C. Register, 291.
Gerry, G. A., on the Hodge-Weber patent, 634.
Gestation and implantation, E. C. Kirk, 96.
Gifts to the profession, W. G. A. Bonwill, 491.
Gilbert, S. E., on correction of irregularities, 373.
on galvanic current, 366.
stopping as covering for arsenious acid, L. A. Faught, 296.
C. E. Hopkins, 299.
Glass tube-tests in root-filling, Jos. Head, 370.
J. D. Patterson, 286.
James Truman, 285.
Globular formations in dentine, R. R. Andrews, 193, 196.
editorial, 240.
Glue-giving basis-substance, Fr. Abbott, 133.
Gold and alloys, W. H. Dwinelle, 176.
as filling material, J. N. Crouse, 413.
George A. Maxfield, 416.
S. B. Palmer, 689.
as root-canal filling, A. C. Boice, 287.
J. N. Crouse, 226.
Gold as root-canal filling, W. H. Dwinelle, 230.
Jos. Head, 224, 278.
S. G. Perry, 223.
G. F. Root, 587.
Ambler Tees, 292.
James Truman, 284.
bands in regulating, E. H. Angle, 324.
bridge patent, the, J. N. Crouse, 478.
caps for worn-away teeth, H. H. Edwards, 693.
cohesive, S. G. Perry, 141.
facing for amalgam fillings, F. S. Bassett, 602.
for copper fillings, J. W. Russell, 654.
fillings in children's teeth, S. B. Palmer, 387.
S. G. Perry, 148.
saturated with chloride of zinc, H. C. Register, 291.
soft, S. G. Perry, 141, 147.
splint in implantation, B. A. R. Ottolengui, 248.
the best? S. B. Palmer, 889.
transition of, from cohesive to soft, James Truman, 276.
use of, in large masses, S. G. Perry, 146.
waste of, S. G. Perry, 141.
Watt's Crystal, S. G. Perry, 141.
Gomphosis, Fr. Abbott, 128.
Gorgas's "Dental Medicine," notice of, 435.
Gothic domes, S. G. Perry, 71.
Gouty patients and peridontitis, Jos. Head, 283.
teeth, R. C. Newton, 577.
C. N. Peirce, 614.
James Truman, 612.
Government bureaux (editorial), 112.
pensions (editorial), 112.
Go West? (editorial), 300.
Grades in dentistry, S. B. Palmer, 384.
Gradual-stopping, Flagg's method of, Jos. Head, 281.
Graduation, standard for, A. G. Bennett, 589.
"Grain" of the enamel, J. N. Farrar, 268.
Granulation in implantation, Fr. Abbott, 128.
Granulating surfaces, protection for, Thos. Fillebrown, 218.
Grape-cure, and destruction of teeth, W. D. Miller, 311.
Gratuitous services, J. G. W. Werner, 471.
I. J. Weatherby, 472.
Great grinding arch, the, of bicuspids, S. G. Perry, 139, 142.
Green's, W. P., exhibit, 108, 237.
on Dr. Robert's paper on electricity, 737.
Greenwood, Dr. John, old advertisement 380.
Greetings, 406.
Grinding surfaces, treatment of, S. G. Perry, 66.

Gross, Prof., on the Bonwill surgical engine, 496.
Grounds against the inflammatory theory of caries, W. D. Miller, 310.
Guilford, S. H., "Physiology of Tooth Movement in Regulating," 395.
"Voluntary Tooth Movement resulting in Excessive Interdental Spaces," 149.
on galvanic current, 366.
on nitrous oxide, 35.
on sterilized sponge as pulp capping, 473.
on the matrix, 174.
on tooth movement in regulating, 395.
Guilford's matrix, S. G. Perry, 146.
"Orthodontia," notice of, 690.
Gulliver's travels, C. C. Barker, 257.
Gum-color, Elias Wildeman's, J. Hayhurst, 103.
colored porcelain inlays, A. H. Thompson, 318.
cutter, Loveland, 161.
sandarach, use of, in regulating, W. G. A. Bonwill, 80.
Gutta-percha, C. S. Beck, 670.
J. N. Crouse, 414.
S. H. Guilford, 175.
S. B. Palmer, 389.
S. G. Perry, 143.
aniline tests, Jos. Head, 279.
J. D. Patterson, 286.
James Truman, 285.
as covering for arsenious acid, F. S. Bassett, 299.
L. A. Faught, 296.
Louis Jack, 296.
as pulp-capping, S. H. Guilford, 473.
as root-canal filling, A. C. Boice, 287.
Jos. Head, 292.
J. D. Patterson, 286.
G. F. Root, 587.
Ambler Tees, 292.
expansion of, J. N. Crouse, 414.
S. G. Perry, 143.
for sensitive teeth, S. B. Palmer, 388.
in approximal cavities, S. H. Guilford, 175.
in children's teeth, S. B. Palmer, 387.
in spreading the jaws, W. G. A. Bonwill, 76.
leakage of, L. A. Faught, 296.
Jos. Head, 309.
over capped pulps, C. S. Beck, 670.
W. B. Miller, 671.
points, S. G. Perry, 223.
stay plate in regulating, W. G. A. Bonwill, 77.
Guy's hospital, dental department, Walter Harris, 247.

H.

Hair and teeth, analogous development of, C. Heitzmann, 553.
W. X. Sudduth, 549.
dissimilarity in development of, C. Heitzmann, 553.
development of, W. X. Sudduth, 548.
Hand mallet, the, E. C. Kirk, 520.
J. A. Libbey, 538.
Hard filling over exposed pulp, C. S. Beck, 670.
rubber corundum disk, W. G. A. Bonwill, 492.
Harris's "Principles and Practice of Dentistry," notice of, 435.
Harrower, A. B., on arsenical poisoning, 298.
Harshley, Prof., on the Bonwill surgical engine, 496.
Haversian canal and osteoblasts, microscopic appearance of, W. X. Sudduth, 544.
Hayden Dental Society, officers elected, 572.
Hayhurst, J., "Dental History," 102.
on death of R. V. Jenks, 750.
Head, Joseph, on cotton as root-canal filling, 224, 277, 284.
on dental assertion and discussion, 524.
on patents, 497.
Headaches from impacted teeth, A. P. Brubaker, 644.
Healing process in regulating, E. H. Angle, 326.
Health of soft tissues in bridge-work, J. M. Edmunds, 656.
Heated air, compressed, Jas. Truman, 213.
Register's method, 213.
broach as disinfectant, J. H. Wooley, 315
Heavy foil, J. N. Crouse, 175.
Heitzmann, C., on Dr. Sudduth's paper on "The Individuality of the Biological Cell," 551.
on the cellular doctrine, 552.
Hemorrhage in antrum, B. A. R. Ottolengui, 369.
Jas. Truman, 369.
from perforation of antrum, E. C. Kirk, 421.
in implantation, case of, B. A. R. Ottolengui, 342, 345.
Herbert method, the, C. S. Beck, 539.
Hereditary conditions of tooth-structure, Salter on, R. R. Andrews, 514.
mal-relation of jaws, S. H. Guilford, 151.
syphilis, first dentition in, R. C. Newton, 577.
the teeth in, R. C. Newton, 576.
C. N. Peirce, 613.
tendencies in the tissues, W. X. Sudduth, 555.
of individual cells, W. X. Sudduth, 540.
Heredity, L. A. Faught, 354.
B. A. R. Ottolengui, 43.
C. N. Peirce, 331.
W. X. Sudduth, 46.
R. B. Winder, 41.
a factor in caries, L. A. Faught, 354.
W. C. Wardlaw, 701.
laws controlling, C. N. Peirce, 331.
Heroic method, the, of pulp extirpation, Jas. Truman, 205.
Hip-joint disease and molar eruption, A. P. Brubaker, 644.

Histological development of dentine, C. N. Peirce, 331.
points in implantation, W. X. Sudduth, 97.
work of G. V. Black, Jas. Truman, 739.
Histology, A. G. Bennett, 590.
History, dental, J. Hayhurst, 102.
Hodge-Weber patent, the, G. A. Gerry, 634.
"Home Gymnastics for the Well and Sick," by E. Angerstein, notice of, 433.
Homogeneous condensation of foil, by Bonwill's method, E. C. Kirk, 523.
Hopkins, Chas. E., on arsenious acid, 299.
Horses' teeth, caries in, W. D. Miller, 244.
Hot air in root-canal treatment, H. C. Register, 291.
in intentional pulp devitalization, Wm. H. Trueman, 456.
House, a, for dental societies, Wm. H. Atkinson, 462.
W. X. Sudduth, 470.
Howard University, dental department, commencement of, 303.
How to adjust Woodward's separator, 90.
wash the hands, B. A. R. Ottolengui, 337.
How, W. Storer, clinic, 238.
on some phases of dental literature, 466.
How, why, and what? A. G. Bennett, 598.
Human ovum, the, W. X. Sudduth, 546.
race, improvement of, James Truman, 39.
"Human Physiology," by Landois and Stirling, notice of, 689.
Hyaline cartilage, microscopic appearance of, W. X. Sudduth, 543.
Hydraulic press for stamping plates, E. A. Bogue, 391.
Hydriodic acid, James Truman, 211.
Hydrogen, sulphide of, action of, on iodine, James Truman, 211.
Hydrochlorate of spermine, 626.
Hydronaphthol, W. X. Sudduth, 94.
for instruments, B. A. R. Ottolengui, 337.
Hygiene, G. J. Fredricks, 190.
"Hygiene of the Nursery," by Louis Starr, notice of, 434.
Hygienic relations of artificial dentures, E. C. Kirk, 199.
Hypersensibility of molar, case of, H. H. Edwards, 436.
Hypoblast, development of, W. X. Sudduth, 547.
"Hysteria and Epilepsy," by J. Leonard Corning, notice of, 118.
Hysteria from dental irritation, A. P. Brubaker, 644.

I

Ice age, the, Wm. H. Atkinson, 38.
Dr. Brown, 43.
J. T. Codman, 28.
Ichthyosis of the mouth, James E. Garretson, 349.
Ideal arch, the, E. H. Angle, 324.
dentist, the, A. G. Bennett, 591.
filling, the, S. G. Perry, 139.
in proximate cavities, S. G. Perry, 139.
material, the, E. B. Daire, 273.
the, in prosthetic dentistry, W. W. Allport, 317.
reader, the, A. G. Bennett,
root-canal filling, the, Joseph Head, 608.
successful case, the, Wm. H. Trueman, 451.
Idiocy, the teeth in, R. C. Newton, 578.
C. N. Peirce, 615.
James Truman, 611.
Idiosyncrasies, W. H. Dwinelle, 98.
"I don't know," S. B. Palmer, 385.
Illinois State Board of Dental Examiners, 255.
Illuminating apparatus, J. A. Woodward, 238.
Immediate pulp extirpation, Jas. Truman, 205.
root filling (editorial), W. C. B., 242.
S. G. Perry, 223.
H. C. Register, 289.
G. F. Root, 586.
Wm. H. Trueman, 448.
James Truman, 207, 210, 214.
after use of arsenic, James Truman, 207.
separation, S. H. Guilford, 174.
Immobility of teeth, securing, after regulating, S. H. Guilford, 403.
Impacted cuspid and eye-twitching, C. S. Beck, 664.
and squint-eye, E. C. Kirk, 664.
teeth and reflex disorders, A. P. Brubaker, 644.
wisdom teeth, J. S. Fogg, 366.
S. H. Guilford, 366.
Dr. Jefferis, 91.
E. P. Quick, 365.
Implantation surgically considered, W. C. Barrett, 242.
E. A. Bogue, 312.
L. C. Bryan, 245, 567.
B. H. Catching, 191.
Dr. Cornwall, 100.
G. L. Curtis, 85, 108.
Dr. De Lange, 97.
W. H. Dwinelle, 98.
Dr. Jefferis, 91.
E. C. Kirk, 94, 107.
B. A. R. Ottolengui, 336, 368.
C. N. Peirce, 91.
H. C. Register, 92.
Dr. Smith, 101.
H. A. Smith, 382.
W. X. Sudduth, 97.
J. Taft, 128.
James Truman, 367.
absorption of root in, G. L. Curtis, 87.
adjusting root in, G. L. Curtis, 87.
anchorage, G. L. Curtis, 87.
anchylosis in, L. C. Bryan, 568.

Implantation and bacteria, E. A. Bogue, 312.
and chronic abscess, L. C. Bryan, 567.
and gestation, E. C. Kirk, 96.
and nutrition, E. C. Kirk, 96.
and scrofulous diathesis, E. C. Kirk, 96.
and sponge grafting, J. Taft, 128.
and tuberculosis, E. C. Kirk, 96.
antisepsis in, G. L. Curtis, 87.
E. C. Kirk, 96, 367.
B. A. R. Ottolengui, 337.
W. X. Sudduth, 97.
Dr. Weld, 337.
benefits of, to profession, G. L. Curtis, 88.
causes of failure in, G. L. Curtis, 85.
case of, G. L. Curtis, 108.
E. C. Kirk, 107.
C. N. Peirce, 91.
H. C. Register, 92.
H. C. Herring, E. C. Kirk, 95.
choice of patient, G. L. Curtis, 87.
of teeth, G. L. Curtis, 87.
commended to scientific dentists, G. L. Curtis, 88.
conditions for permanency, E. C. Kirk, 95.
contraindications, E. C. Kirk, 96.
credit due to Dr. Younger, G. L. Curtis, 88.
dangers in, James Truman, 367.
data desired, H. A. Smith, 382.
discussion of Dr. Ottolengui's paper on, 367.
failures, E. C. Kirk, 94.
favorable conditions, G. L. Curtis, 86.
filling root-canal, G. L. Curtis, 86.
forming sockets, G. L. Curtis, 87.
B. A. R. Ottolengui, 338.
freshly extracted teeth, why? G. L. Curtis, 86.
for bridge-work, W. X. Sudduth, 98.
granulation in retention, Fr. Abbott, 128.
histological points, W. X. Sudduth, 97.
in abscessed socket, L. C. Bryan, 567.
in atrophied ridge, G. L. Curtis, 86.
inflammatory process in, G. L. Curtis, 87.
E. C. Kirk, 96.
in natural socket, H. C. Register, 92.
legal liabilities, G. L. Curtis, 101.
W. X. Sudduth, 98.
medical aspect of, W. X. Sudduth, 98.
method of attachment, Fr. Abbott, 128.
L. C. Bryan, 567.
W. X. Sudduth, 97.
of molar root with Logan crown for bicuspid, L. C. Bryan, 567.
of teeth devoid of pericementum, L. C. Bryan, 567.
of third molars, B. A. R. Ottolengui, 347.
pain of, Dr. Cornwall, 100.
E. C. Kirk, 101.
Implantation, pathological points in, W. X. Sudduth, 97.
perforation of antrum in, G. L. Curtis, 87.
E. C. Kirk, 421.
B. A. R. Ottolengui, 341.
porcelain roots, L. C. Bryan, 246.
practical side of, G. L. Curtis, 85.
preparation of tooth for, G. L. Curtis, 86.
promise for the future, E. C. Kirk, 97.
pus formation, E. C. Kirk, 96.
reproduction of alveolar wall after, L. C. Bryan, 568.
scientific aspect of, G. L. Curtis, 85.
septic influences in, G. L. Curtis, 86.
splint for, E. H. Angle, 379.
staple of platinum wire, G. L. Curtis, 108.
use of, G. L. Curtis, 87.
Dr. Smith, 101.
teeth for, Dr. Wetzel, 246.
from battle-fields for, 246.
from dissecting-room for, 246.
how long extracted? E. C. Kirk, 95.
ultimate success, G. L. Curtis, 85.
E. C. Kirk, 96.
use of clasp, G. L. Curtis, 87.
use of cocaine, G. L. Curtis, 108.
use of staples, G. L. Curtis, 87.
with deficient process tissue, C. N. Peirce, 91.
Importance of the peridental membrane in regulating, S. H. Guilford, 398.
of teeth, Prof. N. S. Davis, R. C. Newton, 578.
Important dispatch from J. N. Crouse, 376.
Impression for operation on fissure of soft palate, James E. Garretson, 348.
for retaining splints in implantation, B. A. R. Ottolengui, 348.
Improved separator for the incisors, cuspids, and bicuspids, W. A. Woodward, 89.
Inborn elements, S. B. Palmer, 384.
Incandescent circuit for running the mallet, H. E. Roberts, 710.
current, the, H. E. Roberts, 709.
for the mallet, C. S. Beck, 734.
H. E. Roberts, 734.
Incidents of office practice, E. A. Bogue, 185.
S. E. Gilbert, 373.
Dr. Jefferis, 91.
C. N. Peirce, 91.
B. F. Place, 372.
H. C. Register, 92.
Incipient abscess, tapping the root, L. A. Faught, 296.
Incisor tooth in the nostril, S. A. White, 701.
lower, implantation of, B. A. R. Ottolengui, 346.
single outstanding, regulation of, E. H. Angle, 327.
Increase in price of platinum (editorial), 431.
of density in tooth substance, E. C. Kirk, 357.

Increase of density in tooth substance, C. N. Peirce, 361.
James Truman, 362.
Independent *vs.* trade journalism (editorial), 564.
reply to *Cosmos* on, 686.
Index of dental literature wanted, W. S. How, 467.
India-rubber plates, W. G. A. Bonwill, 10.
Indian skull with copper plate, Geo. F. Waters, 417.
Indiana Dental College Commencement, 301.
Indictment of Dr. H. A. Parr, 254.
Individuality of the biological cell, W. X. Sudduth, 539.
discussion of, 551, 555.
of tissues, C. N. Peirce, 330.
Infantile paralysis from dental irritations A. P. Brubaker, 644.
Infection (editorial), 180.
Inferior dental nerve-canal, location of, B. A. R. Ottolengui, 346.
excision of, Thos. Fillebrown, 214.
Inflamed gingivæ and pyorrhœa alveolaris deposits, George S. Allan, 717.
Inflammation, chronic, of inferior dental nerve, Thos. Fillebrown, 215.
cold compression for, S. G. Perry, 87.
in implantation, G. L. Curtis, 87.
in non-vascular tissues, 309.
W. X. Sudduth, 166.
of dentine, W. X. Sudduth, 309.
S. B. Palmer, 388.
of nasal membrane, Seiler's spray in, 192.
of pericementum, James Truman, 214.
of pulp, Wm. H. Trueman, 449.
Inflammatory conditions in caries, George S. Allan, 137.
diseases of the eye, caused by the teeth, A. P. Brubaker, 640.
results of pulp irritation, James Truman, 203.
theory of caries, George S. Allan, 138.
W. D. Miller, 310.
Influence of acid foods, W. D. Miller, 311.
of medicines, W. D. Miller, 313.
of lesions of the pulp, James Truman, 203.
of temperament in implantation, G. L. Curtis, 87.
of wisdom teeth in disease, J. Y. Crawford, 127.
Infirmary of dental college, L. P. Ingersoll, 191.
Inglis, Otto E., on arsenious acid, 299.
on exfoliation, 299.
Inherited peculiarities of interdental spacing, S. H. Guilford, 151.
tendencies, R. R. Andrews, 516.
Inhibitory agents, James Truman, 93.
Injury from opening into antrum, Joseph Head, 424.
E. C. Kirk, 423.
H. C. Register, 425.
James Truman, 423.
Injunctions, 637.
Inlays and gum-colored porcelain, A. H. Thompson, 318.
from broken teeth, Dr. Dorrance, 317.
Inoculation, A. L. Loomis, 261.
by germs, Dr. Semmola, 260.
Insanity from dental irritation, A. P. Brubaker, 644.
of dental origin, J. C. M. Hamilton, 666.
Inspiration, G. L. Curtis, 85.
Instantaneous-break dental engine, George W. Whitefield, 439.
Instruments for implantation, B. A. R. Ottolengui, 368.
for removal of deposits in pyorrhœa alveolaris, George S. Allan, 721.
new, exhibit of, by American Academy of Dental Science, 158.
sterilization of, James Truman, 211.
Insulator, an, for exposed pulp, J. N. Farrar, 270.
Intentional devitalization of the dental pulp, Wm. H. Trueman, 447.
Interchange of ideas, E. J. Essig, 164.
Interdental spaces, S. H. Guilford, 149.
etiology of, S. H. Guilford, 150.
fan-shaped, S. H. Guilford, 154.
gradual formation of, S. H. Guilford, 152.
inherited, S. H. Guilford, 150.
Interglobular spaces, R. R. Andrews, 517.
C. N. Peirce, 332.
editorial, 241.
Internal decay of teeth, Kelly on, R. R. Andrews, 513.
International Dental Congress, E. Brasseur, 377.
P. Du Bois, 187.
editorial, 374.
Dental Register on, 506.
Minnesota State Dental Society on, 503.
New Jersey State Dental Society on, 503.
Pennsylvania State Dental Society on, 505.
Southern Dental Journal on, 506.
a second, J. S. Marshall, 695.
in America, W. W. Allport on, 504.
American Journal of Dental Surgery on, 506.
A. E. Baldwin on, 505.
B. H. Catching on, 506.
Chicago Dental Club on, 503.
editorial, 502.
John S. Marshall on, 695.
C. Stoddard Smith on, 505.
sections of, P. Du Bois, 187.
officers of, P. Du Bois, 188.
Medical Congress, Dental Section, American Medical Association on, 503.
E. Brasseur on, 377.
P. Du Bois, 187.
editorial, 374.
George P. E. Kuhn, 245.

International Dental Journal and the Chicago Dental Club, 764.
and the Dental Protective Association, W. X. Sudduth, 486.
Vol. X. (editorial), 753.
Tooth Crown Company, J. N. Crouse, 478.
G. L. Curtis, 482.
G. A. Maxfield, 483.
H. C. Merriam, 485.
L. D. Shepard, 483.
W. X. Sudduth, 486.
T. S. Waters, 190, 203.
and Dr. Crouse, 764.
relief from domination of, T. S. Waters, 203.
*vs.* J. N. Crouse, 254.
*vs.* H. A. Parr, 254.
Interrupted pressure in regulating, S. H. Guilford, 400.
Inventions, American (editorial), 110.
Inventive genius, protection for, L. C. Bryan, 246.
Inventors, C. J. Essig, 165.
Inventing small pieces for soldering, J. Bond Littig, 698.
Invoice your knowledge, S. B. Palmer, 385.
Iodide of bismuth, James Truman, 212.
Iodine, action of hydrogen sulphide on, James Truman, 211.
and aconite in root-canal treatment, G. F. Root, 586.
tincture of, in arsenical ulcerations, L. A. Faught, 297.
Iodoform, L. A. Faught, 225.
T. Fillebrown, 217.
James Truman, 210.
action of, in putrefactive conditions, James Truman, 212.
and eucalyptus, C. M. Bailey, 315.
as a disinfectant, C. M. Bailey, 315.
in pulp treatment, James Truman, 206.
in root-canals, A. G. Bennett, 289.
paste, James Truman, 211.
Iodol paste for root-canal filling, Van Woert, 372.
Iron in copper amalgam, J. W. Russell, 653.
Irregularities of the teeth, S. H. Guilford, 149.
causes of, R. C. Newton, 580.
from lip-sucking, T. H. Parramore, 419.
from mouth-breathing, C. N. Peirce, 616.
W. X. Sudduth, 419.
S. C. G. Watkins, 419, 617.
from serumal tartar in pyorrhœa alveolaris, George S. Allan, 723.
from laws described, S. E. Gilbert, 373.
from methods of correcting, W. G. A. Bonwill, 10, 76.
E. H. Smith, 320.
nutrition a factor in, W. G. A. Bonwill, 17.
Irritation of the gums, S. G. Perry, 68.
of pulp, causes of, James Truman, 206.
inflammatory results of, James Truman, 203.
products of, James Truman, 170.
Ivory for artificial teeth, J. Hayhurst, 102.
Ivory, J. W., case of replantation, 97.
exhibit, 108.
napkin clamps, 237.
rubber-dam clamps, 237.

J.

Jack's matrix, S. H. Guilford, 145.
Jackscrew for elevating fracture of the skull, W. G. A. Bonwill, 493.
Jack spring, W. G. A. Bonwill, 20.
Japanese bibulous paper in packing amalgam, F. S. Bassett, 601.
Jaw, development of, W. X. Sudduth, 550.
adjustable for clamps, W. H. Rollins, 186.
Jefferis on implantation, 92.
Johns Hopkins University (editorial), 112.
Johnstown calamity, the, 440.
Joints, bevelled, for forceps, Dr. Ham, 161.
rounded, for forceps, V. C. Pond, 159.
Journal, the, a factor in dental education, A. G. Bennett, 590.
dental, literature of, A. G. Bennett, 589.
the, and the Dental Protective Association, J. N. Crouse, 482.
Journalism, independent (editorial), 52.
Jumbo glass, the, S. G. Perry, 148.
"Jumping" a tooth, J. N. Farrar, 271.
Justi, H. D., exhibit, 237.

K.

Kansas City Dental College, commencement of, 301.
Kaolin, what is it? W. H. Atkinson, 105.
Karline Hoy, 253.
Karyokinsis, W. X. Sudduth, 51.
Keeping shapes of teeth in proximal surfaces, S. G. Perry, 141.
Keller Medicine Company, exhibit, 236.
Kells, C. E., electrical appliances, 115.
Kelly on decay, 513.
King, James S., treatment of exposed pulps with a view of preservation, 649.
Kingsbury & Andrew's teeth, W. H. Atkinson, 105.
Kingsley on the development of the permanent teeth, 580.
Kirk, E. C., clinic, 107.
on cocaine, 99.
on Dr. Brubaker's paper on "Reflex Effects of Dental Irritation," 663.
on Dr. Head's paper on "Dental Association," etc., 533.
on exostosis, 664.
on hygienic relations of artificial dentures, 199.
on implantation, 94, 367.

Kirk, E. C., on the Bonwill method of packing gold foil, 519.
on tobacco, 93.
Klapp's matrix, S. G. Perry, 146.
Knobs of gold, S. G. Perry, 68.
Koch, laboratory work of (editorial), 180.
Kolliker on histology, 553.
Kratzer, C. V., on Dr. Head's paper on "Dental Assertion," etc., 534.

L.

Lactic acid, George S. Allan, 130.
editorial, 180.
H. C. Register, 532.
absorption of lime salts, George S. Allan, 131.
in artificial caries, George S. Allan, 132.
Laminated fillings, J. N. Farrar, 266.
Lancet-beak forceps, Ambler Tees, 497.
Landor's "Human Physiology," notice of, 690.
Lanolin and arsenious acid, A. B. Harrower, 298.
Lanugo hairs, W. X. Sudduth, 549.
Lateral incisor, superior, precautions in implantation, B. A. R. Ottolengui, 340.
Larynx, neuroses of, of dental origin, A. P. Brubaker, 643.
Law of correspondence and temperament in relation to selection of artificial teeth, 692.
of failure, S. B. Palmer, 388.
of success, S. B. Palmer, 388.
Laws governing electric currents, H. E. Roberts, 703.
L.D.S. degree, Frank Harrison, 409.
Lead cap over arsenious acid, James Truman, 296.
poisoning, the teeth in, R. C. Newton, 578.
Leakage of gutta-percha fillings, L. A. Faught, 296.
Joseph Head, 292, 370.
James Truman, 292.
of oxychlorate and oxyphosphate fillings, Joseph Head, 370.
of root-canal filling materials, Joseph Head, 608.
Leaky gold fillings, S. B. Palmer, 389.
Lectures, public, course of, Odontological Society of Pennsylvania (editorial), 688.
system, the, L. P. Ingersoll, 191.
Leland Stanford University (editorial), 112.
Lemon-cure, the, and destruction of teeth, W. D. Miller, 311.
Length of root, to determine, in implantation, B. A. R. Ottolengui, 339.
Lengthening of college term (editorial), 308.
Leptothrix buccalis, C. N. Peirce, 168.
Lessons taught by "the new departure," E. B. Davis, 272.
"Let it alone," James E. Garretson, 350.
Leucocytes, W. H. Atkinson, 37, 228.
C. C. Barker, 263.
James Truman, 232.
Leucoplakia buccalis, James E. Garretson, 349.
Leverage in regulating, J. N. Farrar, 265.
Liability to fracture of devitalized teeth, Wm. H. Trueman, 451.
Liberal education, time requisite for, E. H. Smith, 321.
Library, a, a necessity, W. Storer How, 468.
the dentist's, S. B. Palmer, 386.
Licensing power, where should it lie? (editorial), 308.
Licentiate dental surgeon degree, Fr. Harrison, 409.
Ligatures in regulating, W. G. A. Bonwill, 77.
to hold, W. G. A. Bonwill, 12, 80.
Light and illumination in proximate surfaces, S. G. Perry, 148.
Light to the Gentiles, W. S. Elliott, 726.
discussion of, 747.
Liliput furnace, Ambler Tees, 497.
Liliputian fauna and flora, C. C. Barker, 257.
Lime-salts, manner of deposit of (editorial), 239.
water, beneficial effects of, upon the teeth, Ambler Tees, 364.
Lining cavities, soft foil for, E. B. Davis, 273.
membrane of antrum, E. C. Kirk, 422.
Lip-sucking, T. H. Parramore, 419.
I. J. Weatherby, 420.
Liquid beef tonic, formula, 125.
Lister, Prof., and antiseptics, A. G. Bennett, 597.
Listerine, E. C. Kirk, 94.
in antral disease, L. A. Faught, 426.
method, James Truman, 210.
Litmus-paper, tests in the mouth, C. N. Peirce, 333.
Littig, J. Bond, on sterilized sponge in pulp capping, 476.
Living and dead bone, the difference in touch of, S. A. White, 637.
matter in tooth substance, Fr. Abbott, 133.
tissues in dentine, Fr. Abbott, 309.
Local applications in pyorrhœa alveolaris, Geo. S. Allan, 723.
changes preceding pulp devitalization, Wm. H. Trueman, 455.
effects of arsenious acid, L. A. Faught, 297.
irritants in pyorrhœa alveolaris, Geo. S. Allan, 717.
malaria from abscess of upper incisor! E. H. Smith, 322.
Locke's block teeth, Dr. Fowler, 104.
Lock-jaw and tooth extraction, J. C. Green, 667.
from dental irritation, E. C. Kirk, 663.
Logic of experience, the, S. G. Perry, 74.
Long-pin teeth, L. C. Taylor, 648.
Looking ahead (editorial), 308.
at both sides, A. G. Bennett, 599.

Loose teeth as supports in bridge-work, E. P. Brown, 678.
J. M. Edmunds, 678.
C. S. Stockton, 684.
S. C. G. Watkins. 678.
bridge-work for, E. Parmly Brown, 678.
holding firm, M. L. Rhein, 381.
in pyorrhoea alveolaris, Geo. S. Allan, 714.
roots, treatment of, J. M. Edmunds, 657.
Lord, Benjamin, on cutting away the teeth, 74.
Loss of density in tooth structure, E. C. Kirk, 357.
Lost gifts to the profession, Jos. Head, 497.
Louisiana State Dental Society, proceedings of, 250.
Low patent, the, G. L. Curtis, 482.
T. S. Waters, 203.
bridge, the, J. N. Crouse, 479.
J. M. Edmunds, 656.
Lower animals, tissues of, photo-micrographs, W. X. Sudduth, 45.
jaw, implantation in, precautions, B. A. R. Ottolengui, 346.
Lowered nerve tone and the teeth, L. A. Faught, 355.
W. X. Sudduth, 355.
Ludwig, R. Y., suspension engine, 765.
Lymphatic circulation in cornea, W. X. Sudduth, 309.
patients and peridontitis, Joseph Head, 283.
system of pericementum, S. H. Guilford, 397.
Lymphatics of pulp, S. H. Guilford, 396.

M.

Mackenzie and Frederick III., Wm. H. Atkinson, 50.
light-concentrator, 238.
Macleod, W. B., letters from, 61, 758.
Magill, W. E., on Bonwill's method for frail walls, 536.
on Dr. Head's paper on "Dental Assertion," etc., 533.
band, the, S. H. Guilford, 403.
Magitot on congenital imperfection of structure, 515.
Magnesia in arsenical ulceration, L. A. Faught, 298.
Malaria and pyorrhœa alveolaris, George S. Allan, 719.
Malformations and decay, W. X. Sudduth, 354.
as a cause of caries, W. C. Wardlaw, 701.
Mallet, the automatic, S. G. Perry, 147.
the Bonwill mechanical, S. G. Perry, 147.
Mal-nutrition of peridental membrane, M. L. Rhein, 619.
occlusion a factor in voluntary tooth-movement, S. H. Guilford, 157.
Mal-practice, S. B. Palmer, 386.
in extraction, James Truman, 6.
"Man and his World," by Dr. James E. Garretson, notice of, 638.
Mandibular action, normal, W. G. A. Bonwill, 18.
Manipulative ability in dentistry, E. H. Smith, 319.
art, time required to acquire, E. H. Smith, 321.
Manual training, E. H. Smith, 323.
Manufacture of dental supplies (editorial), 429.
Massachusetts State Dental Society, proceedings of, 405, 470.
Masseter muscles, force exerted by, T. S. Waters, 198.
persistent spasm of, from dental irritation, A. P. Brubaker, 643.
Mastication, strain of, on pulpless teeth, J. Morgan Howe, 697.
Materia medica, acquaintance with, L. Ashley Faught, 293.
Matrix, the, S. H. Guilford, 174.
James S. King, 606.
C. V. Kratzer, 604.
W. B. Miller, 603.
S. G. Perry, 144, 146.
G. L. Robb, 603.
J. R. C. Ward, 604.
a new, W. G. A. Bonwill, 490.
advantages of, S. A. Guilford, 174.
crank, a, W. B. Miller, 603.
objections to, S. H. Guilford, 174.
removal of, from amalgam fillings, F. S. Bassett, 603.
L. A. Faught, 602.
W. B. Miller, 603.
G. L. Robb, 603.
securing, S. G. Perry, 145.
use of, in amalgam fillings, F. S. Bassett, 603.
L. A. Faught, 602.
G. L. Robb, 603.
S. G. Perry, 145.
in proximal cavities, S. G. Perry, 144.
wedging, F. S. Bassett, 601.
Maxfield, Dr. George A., on the International Tooth Crown Company, 483.
Maximum of movement, with minimum of pain in regulating, S. H. Guilford, 400.
Maynard on modern dentistry, S. G. Perry, 75.
McLean disks, Thomas Fillebrown, 158.
McQuillen, D. N., on cotton as root-canal filling, 372.
Mechanical dentists, J. M. Edmunds, 655.
elements in success, S. B. Palmer, 391.
extirpation of pulp, G. F. Root, 584.
irritants in pyorrhœa alveolaris, Geo. S. Allan, 719.
mallet, the, H. C. Register, 252.
right-angled plugger for, W. G. A. Bonwill, 890.
practitioner, the, E. H. Smith, 319.
skill, E. H. Smith, 320.

Mechanism of reflex nerve action, A. P. Brubaker, 645.
Meckel's cartilage, microscopic appearance of, W. X. Sudduth, 543.
Medical aspects of the teeth, R. C. Newton, 576.
course, European and American (editorial), 110.
education of early dentists, E. H. Smith, 321.
press, comments of, on extension of dental college course, 563.
profession and patented articles, James Truman, 498.
Medicaments in antral disease, S. H. Guilford, 426.
Medico-dental schools, Walter Harrison, 247.
Medulla oblongata, the, a switch-board, A. P. Brubaker, 646.
Medullary conditions, Geo. S. Allan, 136.
Meharry School of Dentistry, commencement of, 314.
Membrana eboris, the, R. R. Andrews, 195.
Memorial Meeting of the American Dental Association, 1892 (editorial), 627, 765.
John S. Marshall, 695.
Memoriam, E. J. N. Brasseur, 181.
J. J. P. Ostrader, 119.
Leon Rideout, 436, 765.
John W. Scarborough, 119.
Flavius Searle, 181, 437.
Mental strain and the teeth, C. N. Peirce, 616.
J. L. Williams, 580.
"Merck's Index of Fine Chemicals and Drugs," notice of, 432.
Mercurial poisoning from amalgam fillings, G. S. Beck, 668.
Mercuric chloride, James Truman, 210.
bichloride, disguising taste of, Geo. S. Allan, 253.
Mesentery, section of, microscopic appearance of, W. X. Sudduth, 546.
Mesoblast, development of, W. X. Sudduth, 547.
Metamorphosis, retrograde, of tissues (Abbott), Geo. S. Allan, 134.
Metazoa, the, W. X. Sudduth, 540.
Methods and principles, patenting, Jos. Head, 497.
W. X. Sudduth, 496.
of correcting irregularities, W. G. A. Bonwill, 10.
Michael Angelo, S. G. Perry, 71.
Middle ear catarrh of dental origin, E. C. Kirk, 664.
Micro-bacteria, C. C. Barker, 258.
Microbes, Dr. De Chaumont, 262.
A. L. Loomis, 261.
and dental decay, C. N. Peirce, 361.
H. C Register, 531.
W. X. Sudduth, 354.
and digestion, Dr. Pasteur, 263.
and disease, A. L. Loomis, 261.
function of, G. V. Black, 317.
of the alimentary canal, Dr. Vignal, 262.
the battle with, W. S. Elliott, 727.
Micrococci, C. C. Barker, 258.
Micro-organic forms in oral cavity, James Truman, 93.
life in pulp irritation, James Truman, 208.
Micro-organisms, a sequence of caries (Abbott), Geo. S. Allan, 134.
and dead pulps, H. C. Register, 290.
as sole factors of disease, E. M. Hunter, 261.
and dental caries, C. C. Barker, 264.
and disease, Dr. DeBarry, 262.
editorial, 180.
E. M. Hunter, 261.
E. Klein, 262.
putrefaction, James Truman, 210.
cotton as filter for, James Truman, 285.
in carious dentine, George S. Allan, 131.
in devitalized dentine, C. S. Beck, 671.
in pyorrhæa alveolaris, George S. Allan, 720.
W. X. Sudduth, 744.
Jas. Truman, 744.
morphology of, W. X. Sudduth, 688.
of the oral cavity, W. X. Sudduth, 354.
pathogenic (editorial), 180.
rôle of, E. M. Hunter, 262.
Dr. Maguin, 262.
"Micro-organisms of the Oral Cavity," by W. D. Miller, notice of, 691.
Microscope, powers of, E. M. Hunter, 261.
utility of, C. C. Barker, 257.
Microscopic appearance of carious dentine, W. D. Miller, 310.
of Meckel's cartilage, W. X. Sudduth, 543.
of mucous membrane, W. X. Sudduth, 543.
of section of mesentery, W. X. Sudduth, 546.
life in pulp-canal, S. G. Perry, 222.
organisms and decay, E. A. Bogue, 313.
Microtome, the, W. X. Sudduth, 540.
Milestones (editorial), W. C. B., 243.
Miller, W. D., analytic work of, George S. Allan, 131.
list of antiseptics, 211.
synthetic work of, George S. Allan, 131.
work of, James Truman, 210.
Mind cure, the, Dr. Cornwall, 100.
S. E. Weeks, 315.
Minnesota State Dental law, 314.
J. H. Martindale, 370.
Mirror frame, a new, C. P. Wilson, 162.
Missing lower bicuspids, case of, James E. Garretson, 351.
superior lateral incisors, S. H. Guilford, 150.
Missouri Dental College commencement, 301.
Modern dental literature, A. G. Bennett, 588.
Molar root-canals, treatment of, Joseph Head, 277.

Molar root, with Logan crown, implantation of, L. C. Bryan, 567.
teeth as anchorage in regulation, E. H. Angle, 324.
Molars, implantation of, B. A. R. Ottolengui, 346.
Moral character, the basis of, S. B. Palmer, 384.
Morphine and wood creosote for exposed pulps, J. N. Farrar, 271.
habit, the, Erlenmeyer, 690.
Morphology of cells, W. X. Sudduth, 546.
of enamel prisms, C. N. Peirce, 330.
of micro-organisms of the mouth, W. X. Sudduth, 688.
Mother knew ! S. G. Perry, 71.
Mouth and teeth, the, in illness, E. C. Briggs, 632.
-breathing, R. C. Newton, 579.
C. N. Peirce, 616.
James Truman, 611.
S. C. G. Watkins, 419.
and irregularities of the teeth, S. C. G. Watkins, 617.
the, a culture apparatus, H. C. Register, 532.
-washes, antiseptic, value of, C. N. Peirce, 91.
Mouths of canals, enlarging, Jos. Head, 282.
of tubuli, cleansing, H. C. Register, 292.
Movable-jaw matrix, W. G. A. Bonwill, 490.
Movement of apex in regulating, S. H. Guilford, 390.
of the teeth, S. G. Perry, 73.
Moving cuspids in regulating, E. H. Angle, 328.
Mucous membrane, effect of arsenious acid on, L. A. Faught, 296.
microscopic appearance of, W. X. Sudduth, 643.
sanitary condition of, in permanent bridge-work, T. S. Waters, 167.
Mulford, H. K., & Co.'s exhibit, 237.
Mulreany on the connection between hip-joint disease and dentition, A. P. Brubaker, 644.
Muriate tincture of iron in arsenical ulceration, L. A. Faught, 297.
Muscular disorders of dental origin, A. P. Brubaker, 643.
fibres, contraction of, A. P. Brubaker, 643.
Museum of inventions, W. G. A. Bonwill, 494.

### N.

Nagel's observations on the human ova, 546.
Napkin-holder, a, Dr. Hopkins, 162.
Naphthol solution, James Truman, 211.
Narrow clasps, E. A. Bogue, 185.
Nasal catarrh, E. C. Kirk, 96, 422.
National Association of Dental Examiners (editorial), 558.
officers elected, 560.
National Association of Dental Faculties, 510, 560, 766.
officers elected, 564.
proceedings, 560.
resolutions passed, 308.
Nationality as a cause of caries, W. C. Wardlaw, 701.
Natriumsulfibenzoat, E. Heckel, 126.
Natural adaptation, S. B. Palmer, 384.
crowns in crown-work, Theodore F. Chupein, 582.
socket in implantation, H. C. Register, 92.
Nature our guide, S. G. Perry, 68.
Neal's artificial teeth, J. Hayhurst, 103.
Neck caries, W. D. Miller, 311.
Necrosis from arsenious acid, L. A. Faught, 299.
of antral walls, E. C. Kirk, 423.
of lower jaw, Dr. Martindale, 316.
of palate and superior maxillary, result of abscess and a rubber plate, W. D. Babcock, 464.
Nerveache diagnosis, Thos. Fillebrown, 218.
Nerve and crown drills, clinic of, J. G. Morey, 238.
Nerve-canal in inferior maxilla, B. A. R. Ottolengui, 336.
over superior cuspid sockets, B. A. R. Ottolengui, 340.
Nervous connection of the enamel, C. N. Peirce, 331.
influences in decay, L. A. Faught, 355.
prostration, effects of, on the teeth, C. N. Peirce, 334.
W. X. Sudduth, 353.
system, diseases of the, by J. M. Charcot (editorial), 118.
Nervousness and the teeth, R. C. Newton, 581.
Nests for bacteria, E. A. Bogue, 312.
Neuralgia, cases of, 216.
characteristic indications of, Dr. Brackett, 220.
differential diagnosis, Dr. Brackett, 221.
Thos. Fillebrown, 218.
exciting causes, Dr. Brackett, 220.
Thos. Fillebrown, 218.
from dental lesions, E. H. Smith, 322.
from imbedded wisdom teeth, Dr. Jeferis, 92.
of the eyeball, and the teeth, A. P. Brubaker, 641.
of the sciatic plexuses of dental origin, A. P. Brubaker, 644.
Neuralgic pain after implantation, B. A. R. Ottolengui, 343.
from pulp nodules, W. H. Trueman, 449.
toothache, W. H. Fundenberg, 667.
New bone deposits in sockets after extraction, B. A. R. Ottolengui, 342.
departure, lessons taught by, E. B. Davis, 272.
engine and mallet, H. C. Register, 251.
method of excision of inferior dental nerve, Thos. Fillebrown, 215.
pattern handles for forceps, V. C. Pond, 158.

New-Englanders, diet of, J. T. Codman, 26, 44.
teeth of, J. T. Codman, 44.
New England Dental Society, officers elected, 766.
Jersey State Dental Society, proceedings, 37, 94, 539, 611, 673, 739.
on Dental Protective Association, 750.
York College of Dentistry, commencement of, 302.
State Board and the American Dental Association, W. E. Magill, 633.
State Dental Society, 57.
Newton, Richard C., "The Teeth as a Factor in Diagnosis," 575.
Nitrate of amyl, E. C. Kirk, 100.
of silver in treating chronic alveolar abscess, R. B. Adair, 701.
Nitrous oxide, W. B. Macleod, 758.
S. H. Guilford, 35.
C. N. Peirce, 9.
Dr. Thomas, 33.
James Truman, 9.
and diabetes, C. N. Peirce, 9.
Dr. Thomas, 34.
and shock, S. H. Guilford, 35.
and tight lacing, 759.
causes of anxiety in administration of, W. B. Macleod, 758.
death concurrent with administration of, W. B. Macleod, 758.
gas narcosis, W. B. Macleod, 758.
on full stomach, W. B. Macleod, 759.
specialists in, L. A. Faught, 34.
"No such word as fail," S. B. Palmer, 391.
Nodulary dentine, W. H. Trueman, 449.
Noise of mechanical mallet, L. A. Faught, 495.
W. G. A. Bonwill, 495.
D. N. McQuillen, 495.
Jos. Head, 495.
Nomenclature, W. X. Sudduth, 541.
of pyorrhœa alveolaris, Geo. S. Allan, 716.
Non-cohesive gold lining, S. B. Palmer, 389.
secret medicines, 236.
vascularity of enamel, Geo. S. Allan, 137.
of tooth tissue, Geo. S. Allan, 136.
C. N. Peirce, 168.
Normal action of the medulla oblongata, A. P. Brubaker, 646.
class in denistry, J. Taft, 192.
race condition of man, J. T. Codman, 24.
tooth structure, S. B. Palmer, 388.
Northrup, forms of corundum points, W. G. A. Bonwill, 492.
Not good practice, E. C. Kirk, 368.
Note of warning, W. H. Dwinelle, 172.
Novel features of Register's engine, 237.
Number of root-canals, Jos. Head, 277.
Nutrition, diverted, W. G. A. Bonwill, 16.
perverted, W. G. A. Bonwill, 17.
polluted, W. G. A. Bonwill, 16.
and caries, L. A. Faught, 355.
and implantation, E. C. Kirk, 96.
of enamel fibres, C. N. Peirce, 331.
Nutrient currents of dental pulp, Wm. H. Trueman, 455.
supply of dental tissues, S. H. Guilford, 396.
Nutritive conditions. E. C. Kirk, 96.
control, E. C. Kirk, 96.
distribution, interruption of, C. N. Peirce, 330.

O.

Obituary, E. J. N. Brasseur, 181.
T. L. Cook, 749.
F. S. Eggert, 749.
R. V. Jenks, 749.
J. J. P. Ostrader, 119.
Leon Rideout, 437, 765.
J. W. Scarborough, 109.
Flavius Searle, 181, 437.
Objects of the American Dental Trade Association, 442.
of the Dental Protective Association, J. N. Crouse, 478.
Objections to matrices, S. H. Guilford, 174.
Oblique angle in using Bonwill's automatic process mallet, E. C. Kirk, 522.
Obliteration of dentinal tubuli, case of, James Truman, 363.
Obscure symptoms of pyorrhœa alveolaris, George S. Allan, 715.
Obstetrics, study of, by dental students, E. H. Smith, 321.
Obtundents with arsenic in pulp devitalization, G. F. Root, 585.
Obtunding sensitive dentine by electrolysis, D. F. McGraw, 316.
Occipital bandage in regulating, E. H. Angle, 324.
Occlusion, abnormal, S. H. Guilford, 153.
Ocular diseases and the teeth, A. P. Brubaker, 640.
Odontoblasts, the, George S. Allan, 136.
function of, C. N. Peirce, 687.
Odontological Society of Chicago, officers elected, 766.
of New York and the *Cosmos*, 687.
of Pennsylvania, 32, 91, 163, 222, 277, 352, 367, 421, 486.
Ambler Tees on, 119.
clinics, 237.
editorial, 688.
exhibits, 236.
Office of the pulp, W. X. Sudduth, 352.
Officers elected, American Dental Society of Europe, 694.
Chicago Dental Society, 318.
Hayden Dental Society of Chicago, 572.
International Dental Congress, 188.

Officers elected, Louisiana State Dental Society, 251.
Minnesota State Dental Society, 510.
National Association of Dental Examiners, 560.
National Association of Dental Faculties, 564.
National Association of Dental Secretaries, 766.
New England Dental Society, 766.
New York State Dental Society, 438.
Odontological Society of Chicago, 766.
Pennsylvania Association of Dental Surgeons, 702.
Ohio College of Dental Surgery, commencement of, 302.
Ohm, definition of, H. E. Roberts, 704.
Ohm's law, H. E. Roberts, 706.
Oil of cinnamon, W. X. Sudduth, 94.
of peppermint, H. C. Register, 94.
W. X. Sudduth, 94.
of wintergreen, W. X. Sudduth, 94.
Old methods, C. S. Stockton, 104.
plugs, arsenical ulceration after removal of, L. A. Faught, 296.
theories of caries, W. X. Sudduth, 353.
women's remedies, Wm. H. Atkinson, 461.
Older methods in treatment of proximate surfaces, S. G. Perry, 142.
Omnivorous animal, man not an, J. T. Codman, 23.
One idea progress, E. B. Davis, 271.
One of us (editorial), 115.
One horse-power in electricity, H. E. Roberts, 706.
Onward and upward (editorial), 430.
Open root-canals, W. G. A. Bonwill, 287.
Opening the bill, S. H. Guilford, 402.
Operating on approximal surfaces, S. G. Perry, 139.
Operation of drugs, Wm. H. Atkinson, 462.
"Operative Dentistry," by Thomas Fillebrown, notice of, 434.
Oppressive ethical laws, Ambler Tees, 497.
Oral antiseptics, W. X. Sudduth, 94.
hygiene, L. A. Faught, 355.
surgery, ignorance of the general surgeon on, R. C. Newton, 575.
Orbicularis oris muscle, force of, C. H. Hayward, 420.
W. X. Sudduth, 419.
S. C. G. Watkins, 419.
Organ, an, for the profession (editorial), 566.
Organic matter, inflammation of, in teeth, S. B. Palmer, 389.
tissue in the tubuli, James Truman, 214.
Organisms manufacturing acids, Geo. S. Allan, 130.
Organization (editorial), 52.
of American Dental Trade Association, 443.
of New York dentists, 57.
precedes function, W. X. Sudduth, 550.
Origin, bacterial, of caries, Geo. S. Allan, 132.
of collapse, James Truman, 1.
of life, W. X. Sudduth, 46.
of textual doctrine, Wm. H. Atkinson, 461.
of tooth-germs, C. N. Peirce, 330.
Original ideas, credit for, James Truman, 499.
"Orthodontia," by S. H. Guilford, notice of, 510, 691.
W. G. A. Bonwill, 10.
S. H. Guilford, 690.
Osmosis in dentine, 309.
Osmotic circulation in tooth substance, L. C. Bryan, 569.
Ossification of pulp, Wm. H. Trueman, 449.
Ossified pulp-canals, J. C. M. Hamilton, 666.
Osteoblasts, S. H. Guilford, 397.
W. X. Sudduth, 353.
Osteo-odontoma, James E. Garretson, 350.
Ostrader, J. J. P., obituary, 119.
Otalgia from carious wisdom teeth, E. C. Kirk, 664.
Otitis and dentition, E. C. Kirk, 664.
chronic, and the wisdom teeth, A. P. Brubaker, 642.
Ottolengui, B. A. R., "Implantation Surgically Considered," 336.
Our accepted curriculum, E. H. Smith, 321.
final reply on trade journalism (editorial), 756.
imperfect culture (editorial), 110.
institutions (editorial), 112.
position (editorial), 752.
topical index (editorial), 55.
proximate surfaces, S. G. Perry, 139.
Outstanding incisor, regulation of, E. H. Angle, 327.
Oval face pluggers, W. G. A. Bonwill, 492.
Overbite, abnormal, S. H. Guilford, 152.
Overlaps of incisors, W. G. A. Bonwill, 13.
Overlapping teeth, regulation of, J. N. Farrar, 200.
Overmedication, L. A. Faught, 426.
S. H. Guilford, 426.
Overstrain, effects of, James Truman, 4, 7.
Ovum, initial development of, W. X. Sudduth, 546.
Oxide of arsenic, L. A. Faught, 294.
Oxychloride of zinc, R. R. Andrews, 169.
editorial, W. C. B., 242.
H. H. Johnson, 127.
James Truman, 213.
absorption of fluids, Jas. Truman, 292.
as pulp capping, J. N. Crouse, 226.
as root-canal filling, Jos. Head, 292.
J. D. Patterson, 286.
S. G. Perry, 223.
G. F. Root, 587.
A. Tees, 291.
James Truman, 285.

Oxychloride of zinc and bevelled flattened gold wire in root-canals, Sid. Holland, 127.
leakage of, Jos. Head, 309.
linings, S. B. Palmer, 389.
and tooth hardening, R. R. Andrews, 169.
Oxyphosphate of zinc, S. G. Perry, 144.
and pulp devitalization, S. B. Palmer, 389.
leakage of, Jos. Head, 309.
linings, S. B. Palmer, 389,

P.

Pain after extraction, cause of, B. F. Place, 273.
pulp capping, James S. King, 669.
Painless destruction of pulp, James Truman, 206.
protection of exposed pulps, J. N. Farrar, 271.
Palmer, S. B., "Elements of Success in Dental Practice," 383.
Papilla of hair, formation of, W. X. Sudduth, 548.
the dental, C. N. Peirce, 331.
Paraffine as root-canal filling, E. C. Kirk, 293.
Paralysis from dental irritation, A. P. Brubaker, 644.
of ocular muscles from dental irritation, A. P. Brubaker, 641.
Paralyzing agents in pulp extirpation, James Truman, 206.
Paris Exposition, G. P. E. Kuhn, 245.
Parke, Davis & Co.'s exhibit, 236.
Parr, H. A., suit against, 254.
remarkable bridge-work, 238.
Parramore, T. H., on "Sterilized Sponge for Capping Pulps," 393.
Partial plates, clasps for, E. A. Bogue, 185.
Partz battery for dental mallet, L. A. Faught, 735.
Patent laws of Switzerland, L. C. Bryan, 246.
Patentees in the dental profession, W. G. A. Bonwill, 491.
Patenting methods, Joseph Head, 497.
W. X. Sudduth, 496.
Patents in dentistry, W. G. A. Bonwill, 486.
L. C. Bryan, 246.
G. A. Gerry, 637.
Joseph Head, 497.
H. C. Merriam, 250.
W. X. Sudduth, 54, 495.
James Truman, 497.
of the International Tooth Crown Company, J. N. Peirce, 478.
validity of, W. X. Sudduth, 54.
Pathogenic bacteria, C. C. Barker, 259.
Dr. De Chaumont, 267.
micro-organisms (editorial), 180.
James Truman, 93.
conditions of pulp, treatment of, James Truman, 206.
Pathological difficulties in pulp devitalization, James Truman, 206.
Pathological points in implantation, W. X. Sudduth, 97.
predispositions of enamel tissue, C. N. Peirce, 331.
results of implantation, L. A. Faught, 424.
of perforation of the antrum, E. C. Kirk, 421.
state of pulp, management of, James Truman, 204.
Patient, choice of, for implantation, G. L. Curtis, 87.
overtaxing, G. V. Black, 5.
Patterson, J. D., on root-canal filling, 286.
Peabody Museum, visit to, 58.
Peculiarities of inheritance, S. H. Guilford, 150.
Peg and mallet in pulp extirpatian, G. L. Root, 584.
teeth, Dr. Gibney, 577.
Peirce, C. N., on "Conditions which promote or retard the Progress of Caries," 329.
on galvanic current, 366.
on implantation, 91.
on Dr. Newton's paper on "The Teeth as a Factor in Disease," 612.
on nitrous oxide and diabetes, 9.
on protracted operations, 34.
Penetrating antrum in implantation, G. L. Curtis, 87.
E. C. Kirk, 367.
James Truman, 367.
Pennsylvania Association of Dental Surgeons, officers elected, 702.
College of Dental Surgery, commencement of, 302.
Odontological Society, 19, 32, 91, 163, 277, 352, 421, 426.
State Dental Society, 528, 600, 658, 734.
Percentage of dental graduates in practice (editorial), 300.
of graduates to matriculates (editorial), 306.
Percussive force in condensing gold, E. C. Kirk, 537.
Perforation of antrum, pathological results of, E. C. Kirk, 421.
in implantation, E. C. Kirk, 421.
of root, L. A. Faught, 296.
Pericemental inflammation, James Truman, 214.
membrane, implantation of tooth devoid of, L. C. Bryan, 567.
Pericementitis, James Truman, 208, 214.
Pericementum, the, W. H. Atkinson, 235.
S. H. Guilford, 397.
and pulp, relations of, James Truman, 216.
inflammation of, James Truman, 210.
preservative, action of table-salt on, L. C. Bryan, 568.
various functions of, S. H. Guilford, 398.
Peridental membrane, importance of, in regulating, S. H. Guilford, 398.
in implantation, Younger, 97.

Peridental membrane, revitalization of, E. C. Kirk, 95.
vitality of, E. C. Kirk, 91.
Periodic neuralgic pain and tooth-extraction, W. E. Magill, 666.
Periodicity of decay, L. A. Faught, 354.
E. C. Kirk, 358.
C. N. Peirce, 333.
W. X. Sudduth, 353.
Periosteum, building up of, James Truman, 742.
of implanted teeth, L. C. Bryan, 567.
Periostitis from arsenious acid, L. A. Faught, 297.
Perishable teeth, cause of, R. C. Newton, 580.
Permanent bridge-work, A. G. Bennett, 232.
W. H. Dwinelle, 232.
T. S. Waters, 197.
root-canal fillings, Joseph Head, 278.
James Truman, 285.
separations, Theo. L. Chupein, 173.
S. H. Guilford, 174.
C. A. Kingsbury, 173.
S. G. Perry, 66, 69.
splint in implantation, B. A R. Ottolengui, 348.
Permeability of dentine, C. N. Peirce, 167.
Peroxide of hydrogen, James Truman, 211.
in pyorrhœa alveolaris, Geo. S. Allan, 722.
James Truman, 743.
in root-canal filling, G. F. Root, 586.
Perry, S. G., on the treatment of proximate surfaces, 65, 139.
on the matrix, 144.
" Perry's chasm," 70.
Perseverance an element of success, J. N. Crouse, 413.
Persistent force in regulating, S. H. Guilford, 400.
Personal controversies (editorial), 686.
equation, the, S. G. Perry, 67.
Phenol sodique in implantation, E. C. Kirk, 368.
Phenomena of dental caries, W. D. Miller, 310.
Philadelphia County Dental Society, special meeting of, 293.
Dental College, commencement of, 302.
Philosophy of excavating, J. N. Farrar, 264, 268.
Phonograms (editorial), 756.
Phonograph, the, in society meetings (editorial), 754.
Phosphate of zinc and exposed pulps, J. N. Farrar, 270.
Phosphates and the teeth, R. C. Newton, 580.
diminished supply of, W. G. A. Bonwill, 16.
" Photographic Illustrations of Skin-Diseases," by George Henry Fox, notice of, 118.
Photogravure (editorial), 179.
Photo-micrographs, the, of R. R. Andrews, G. S. Allan, 132.
W. H. Atkinson, 553.
editorial, 239.
W. X. Sudduth's, 45.
accuracy of, W. X. Sudduth, 541.
how made, W. X. Sudduth, 540.
Photo-micography (editorial), 179.
Phthisis and pyorrhœa alveolaris, M. L. Rhein, 745.
Physical culture (editorial), 433.
strain, collapse from, James Truman, 6.
Physicians' Leisure Library, " Abdominal Surgery," by H. C. Wyman, 118.
" Bright's Disease," by Alfred L. Loomis, 435.
" Certain Diseases of the Nervous System," by J. M. Charcot, 118.
" Dyspepsia," by Frank Woodbury, 690.
" Hysteria and Epilepsy," by J. Leonard Corning, 118.
" Morphia Habit," by Erlenmeyer, 690.
" Pulmonary Tuberculosis," by David J. Doherty, 435.
" The Radical Cure of Hernia," by H. O. Marcy, 435.
Physics, Wm. H. Atkinson, 38.
Physiological action in movement of a tooth, S. H. Guilford, 398.
changes in surrounding tissues in regulating, S. H. Guilford, 395.
chemistry, W. X. Sudduth, 46.
elements in success, S. B. Palmer, 391.
Physiology of repair, W. S. Elliott, 475.
of tooth movement in regulating, S. H. Guilford, 395.
study of, by dental students, A. P. Brubaker, 640.
Pinney on bridge-work, 680.
Pins, position of, in new forceps, V. C. Pond, 159.
set in cavities, J. N. Farrar, 264.
Pipes and bands in regulating, E. H. Angle, 324, 379.
Pits and fissures of the enamel, R. R. Andrews, 511.
Geo. S. Allan on, R. R. Andrews, 519.
G. V. Black on, R. R. Andrews, 516.
Kelly (1843) on, R. R. Andrews, 513.
Magitot on, R. R. Andrews, 515.
Wm. Robertson (1835) on, R. R. Andrews, 512.
Salter on, R. R. Andrews, 514.
Tomes on, R. R. Andrews, 514.
Wedl on, R. R. Andrews, 515.
location of, R. R. Andrews, 517.
Planishing movement in packing gold foil, E. C. Kirk, 699.
Plastic fillings, E. B. Davis, 272.
in proximate cavities, S. G. Perry, 144.
gold (editorial), W. C. B., 242.
Plate and staples in crown-work, Theo. F. Chupein, 583.
Platinized gold wire in regulating, E. H. Angle, 324.

Platinum bands in regulating, E. H. Angle, 324.
price of (editorial), 431.
supply of (editorial), 431.
Plethoric patients and peridontitis, Jos. Head, 283.
Plugger points for Bonwill's engine mallet, E. C. Kirk, 521.
for the Bonwill method of packing gold foil, E. C. Kirk, 699.
Pointed fissure burr, W. G. A. Bonwill, 492.
Pond's extract in antral disease, L. A. Faught, 426.
forceps, V. C. Pond, 158.
new pattern, handles in, V. C. Pond, 158.
Porcelain bridge-work, E. Parmly Brown, 684.
M. L. Rhein, 683.
T. S. Waters, 682.
fillings, L. L. Davis, 316.
inlays, W. H. Dorrance, 317.
C. Thomas, 317, 439.
A. H. Thompson, 439.
clinic, W. S. How, 238.
gum-colored, A. H. Thompson, 318.
with platinum matrix, C. Thomas. 317.
teeth for implantation, L. C. Bryan, 246.
forming cavities in, H. A. Keely, 509.
Post-graduate course, J. G. W. Werner, 471.
education, A. G. Bennett, 592.
school, a, Wm. H. Atkinson, 463.
Potassa, mercuric iodide solution in implantation, B. A. R. Ottolengui, 348.
Potassium cyanide, action of, on heart, Jordan, 2.
Power applied in regulating, character of, S. H. Guilford, 399.
behind, the, in tooth eruption, S. H. Guilford, 401.
of association, G. L. Curtis, 315.
producing appliances in regulating, S. H. Guilford, 400.
production of, in the mouth in regulating, S. H. Guilford, 399.
Powdered bone food, Parke, Davis & Co., 236.
Practice, office, incidents of, E. A. Bogue, 185.
S. E. Gilbert, 373.
C. R. Jefferis, 91.
C. N. Peirce, 91.
B. F. Place, 372.
H. C. Register, 92.
Practical office work, S. B. Palmer, 386.
points in operating, S. G. Perry, 139.
in treatment of proximate surfaces, S. G. Perry, 139.
side, the, of implantation, G. L. Curtis, 85.
training in dentistry, E. H. Smith, 320.
work, E. H. Smith, 321.
Practitioner and dealer, relations between (editorial), 244.
Precautions in implantation, B. A. R. Ottolengui, 338.
in use of arsenious acid, L. A. Faught, 295.
rubber dam, L. A. Faught, 295.
Predisposing causes of dental caries, George S. Allan, 129.
W. D. Miller, 310.
W. C. Wardlaw, 701.
of decay, C. N. Peirce, 335.
W. X. Sudduth, 354.
Preliminary wedging in treatment of proximal surfaces, S. G. Perry, 143.
Premature eruption of deciduous teeth, W. H. Atkinson, 619.
C. N. Peirce, 612.
Preparation and filling of root-canals, G. F. Root, 584.
of roots and crowns in bridge-work, T. S. Waters, 203.
of tooth for implantation, G. L. Curtis, 86.
Preparatory education, E. H. Smith, 323.
knowledge, thoroughness of, W. H. Atkinson, 460.
Prerequisites for successful teaching, W. H. Atkinson, 747.
Preservation of extracted teeth, Theodore F. Chupein, 583.
President's address, H. C. Register, 528.
Pressure of plate, effects of, on tissues, T. S. Waters, 198.
in regulating, W. G. A. Bonwill, 11.
Prevention of absorption in implanted teeth, G. L. Curtis, 88.
of decay, E. A. Bogue, 313.
of shock in regulating, E. H. Angle, 326.
Price of platinum (editorial), 431.
Primary lesions justifying pulp devitalization, Wm. H. Trueman, 448.
Primitive bridge-work, T. S. Waters, 197.
Primogeniture and the laws of heredity, W. H. Atkinson, 618.
Probes and patience, R. W. Thornton, 125.
Problem of dental decay, George S. Allan, 129.
Process of decay, the, George S. Allan, 309.
Products of micro-organisms, the, Hunter, 263.
of putrefaction and caries, George S. Allan, 135.
Professional atmosphere and morals, H. C. Meriam, 250.
crimes, George S. Allan, 112.
elements in success, S. B. Palmer, 391.
holiday, a, George Cunningham, 117.
Professor Austen on the trigeminus nerve, 580.
Progress of decay from enamel fissures, R. R. Andrews, 511.
in youth, C. N. Peirce, 333.
W. X. Sudduth, 353.

Progress of dental art, C. J. Essig, 164.
caries, C. N. Peirce, 329.
of the past, S. G. Perry, 149.
Progressive dentist, the, S. B. Palmer, 384.
dentistry, E. B. Davis, 271.
the, *vs.* the conservative man, A. G. Bassett, 599.
pathological changes in pulp devitalization, Wm. H. Trueman, 447.
Proliferation of bone cells, G. L. Curtis, 87.
Prominence of superior oral teeth, E. H. Angle, 323.
correction of, J. N. Farrar, 271.
S. E. Gilbert, 373.
percentage of, E. H. Angle, 323.
Proof of success, the, Joseph Head, 526.
Prophecy, the spirit of, W. H. Dwinelle, 172.
Prophylatic treatment, C. N. Peirce, 335.
Prophylaxis, James Truman, 209.
Prosthetic dentistry, E. B. Davis, 272.
J. M. Edmunds, 655.
artistic adaptation, Dr. Comstock, 317.
productions, W. W. Allport, 317.
temperamental aspect of the question, 692.
the ideal in, W. W. Allport, 317.
Prostista, H. C. Register, 531.
Protoplasmic theory, the, George S. Allan, 130.
net-work theory, the, George S. Allan, 134.
Protozoa, W. X. Sudduth, 540.
Protracted operations, L. A. Faught, 34.
S. H. Guilford, 35.
C. N. Peirce, 34.
James Truman, 7.
Protruding teeth, correction of, J. N. Farrar, 271.
Proximal amalgam fillings, use of matrix, F. S. Bassett, 600.
L. A. Faught, 602.
G. L. Root, 603.
Proximate cavities in incisors, S. G. Perry, 144, 147.
in temporary teeth, S. G. Perry, 148.
in very soft teeth, S. G. Perry, 140.
on posterior surfaces, S. G. Perry, 140.
red gutta-percha fillings in, S. G. Perry, 143.
surfaces, aid of mouth mirrors, S. G. Perry, 139.
crystal gold in, S. G. Perry, 141.
importance of subject, S. G. Perry, 66.
of front teeth, S. G. Perry, 147.
treatment of, S. G. Perry, 65, 139.
where decay begins, S. G. Perry, 141.
with extensive decay, S. G. Perry, 139.
with slight decay, S. G. Perry, 139.
Psychic effects of nitrous oxide gas, James Truman, 9.
life of micro-organisms (Binet on), W. X. Sudduth, 542.
Psychial influences, W. X. Sudduth, 32.
James Truman, 9.
Psoriasis, James E. Garretson, 349.
Ptomaines, George S. Allan, 130.
C. C. Barker, 263.
editorial, 180.
bacteria produced, George S. Allan, 138.
in pyorrhœa alveolaris, Geo. S. Allan, 720.
Public inspection of colleges (editorial), 306.
Publication bureau, a, for the profession, W. H. Atkinson, 463.
Pulp, the, S. H. Guilford, 396.
amputation, (editorial), W. C. B., 242.
and dentine, relations of, Jas. Truman, 206.
pericementum, relations of, James Truman, 206.
canals, treatment of, James Truman, 203.
capping, C. S. Beck, 672.
J. N. Crouse, 226.
S. J. Dickey, 298.
W. H. Dwinelle, 229.
C. E. Francis, 227.
S. H. Guilford, 473.
James S. King, 649, 669.
W. B. Miller, 671.
T. H. Parramore, 393, 475.
James Truman, 204, 231.
I. J. Weatherby, 476.
conditions of success, James Truman, 207.
sterilized sponge for, T. H. Parramore, 393.
cases, T. H. Parramore, 475.
material, chloride paper, 650.
Japanese bibulous paper, 474.
gutta-percha, 473.
letter-paper, 474.
Canada balsam and chloroform, 474.
platinum cap and paste, 672.
sterilized sponge, 474.
spunk of white birch, 476.
varnish, 474.
vulcanite, 669.
zinc chloride, 473.
causes of death, James Truman, 206.
chamber, reasons for filling, H. C. Register, 290.
connection of, with tooth body, James Truman, 203.
death of, by gradual process, James Truman, 208.
destruction by escharotics, James Truman, 205.
devitalization, W. H. Atkinson, 228.
C. E. Francis, 227.
W. B. Miller, 671.
G. F. Root, 584.
in regulating, S. H. Guilford, 396.

Pulp exposure, W. B. Miller, 671.
exposed, and varnish, J. N. Farrar, 270.
and wood creosote, J. N. Farrar, 270.
walls of cavity, J. N. Farrar, 270.
function of, W. X. Sudduth, 352.
James Truman, 204.
intentional devitalization, Wm. H. Trueman, 447.
irritation and micro-organic life, James Truman, 208.
causes of, James Truman, 206.
thermal influence, James Truman, 204.
pathological, changes due to, Wm. H. Trueman, 449.
lesions, W. X. Sudduth, 255.
office of, W. X. Sudduth, 352.
pathological state of, James Truman, 204.
preservation, James S. King, 649.
relations of, to surrounding tissues, James Truman, 206, 209.
reparatory action in, R. R. Andrews, 169.
stones, W. H. Atkinson, 228.
strangulation, James S. King, 668.
suppuration, W. B. Miller, 672.
Pulpless teeth, and pyorrhœa alveolaris, Geo. S. Allan, 722.
caries of, Geo. S. Allan, 138.
three conditions found, H. C. Register, 290.
Wm. H. Trueman, 448.
Pus, an inflammation, Geo. S. Allan, 746.
James Truman, 740.
formation, E. C. Kirk, 96.
in pulps, James Truman, 207.
in pyorrhœa alveolaris, Geo. S. Allan, 716, 746.
origin of, W. H. Atkinson, 228.
what is it? James Truman, 740.
Putrefactive conditions and the teeth, Geo. S. Allan, 135.
Putresced pulp, Jos. Head, 281.
Putrescent pulp, chemical changes in, James Truman, 209.
treatment of, in root-canal, G. F. Root, 586.
Pyogenetic conditions, causes of, E. L. Clifford, 761.
Pyorrhœa alveolaris, Geo. S. Allan, 712, 745.
Wm. H. Atkinson, 176.
E. B. Davis, 272.
S. H. Guilford, 177.
E. C. Kirk, 96.
W. D. Miller, 311.
B. A. R. Ottolengui, 43.
C. N. Peirce, 260.
M. L. Rhein, 619, 744.
W. X. Sudduth, 178, 354, 743.
James Truman, 177, 739.
a constitutional affection, M. L. Rhein, 744.
Pyorrhœa alveolaris, a local disease with local cause, George S. Allan, 719.
a misnomer, George S. Allan, 716.
James Truman, 740.
and Bright's disease, M. L. Rhein, 745.
and caries, W. D. Miller, 311.
and decay, C. N. Peirce, 360.
W. X. Sudduth, 354.
and phthisis, M. L. Rhein, 745.
avoidance of, by dentists, George S. Allan, 712.
causes of, M. L. Rhein, 745.
W. X. Sudduth, 743.
chronic, M. L. Rhein, 744.
diagnosis of, M. L. Rhein, 744.
in dog, B. A. R. Ottolengui, 43.
in pulpless teeth, George S. Allan, 722.
origin of, W. X. Sudduth, 743.
James Truman, 742.
probabilities of success in treatment, George S. Allan, 713.
splint for loose teeth in, E. H. Angle, 379.
to hold loose teeth firm in, M. L. Rhein, 381.
treatment of, W. X. Sudduth, 744.
James Truman, 742.

### Q.

Quack, the, J. C. White, 658.
Qualities of eugenol, H. H. Johnson, 127.
Quick, Dr. E. Payson, letter from, 365.
reply to letter from, 366.
Quick-setting amalgam, W. S. Elliott, 730.
copper amalgam, J. W. Russell, 653.
Quinia, James Truman, 210.

### R.

Race peculiarities, American, R. C. Newton, 579.
"Ragnarok," J. T. Codman, 24.
Rainie, Mr., experiments of (editorial), 239.
Rapid breathing as an obtunder, W. G. A. Bonwill, 491.
excavator, A. G. Bennett, 238.
"Rats," John Story, 190.
Reader, the, A. G. Bennett, 595.
Reading, value of, S. B. Palmer, 391.
with the eyes shut, 755.
Reasons for filling the pulp-chamber, H. C. Register, 290.
Recession of capped pulps, James S. King, 669.
G. W. Klump, 669.
of gums in pyorrhœa alveolaris, George S. Allan, 714.
Record and report, R. C. Newton, 576.
Recuperative powers of nature, Wm. H. Trueman, 450.
of the teeth, E. C. Kirk, 357.
C. N. Peirce, 335.
W. X. Sudduth, 352.

Recurrent caries, L. A. Faught, 355.
decay, E. B. Davis, 273.
S. G. Perry, 70.
prevention of, E. B. Davis, 273.
in devitalized teeth, Wm. H. Trueman, 452.
Red gutta-percha, S. G. Perry, 143.
Reducing size of nerve broach, device for, W. G. A. Bonwill, 488.
Reflecting mirror, use of, S. G. Perry, 144.
Reflex dental irritation, J. C. Green, 667.
effects of dental irritation, Albert P. Brubaker, 639.
discussion, Albert P. Brubaker, 662.
nerve action, Geo. W. Whitefield, 731.
Register, H. C., address as President of Pennsylvania State Dental Society, 528.
clinic, 237.
on antiseptics, 93, 291.
on desiccation of dentine, 291.
on the germ theory, 529.
on pulp devitalization, 296.
on root-canal filling, 289.
on tooth decay, 530.
Registry for original ideas, James Truman, 498.
Regulating appliances, Edward H. Angle, 323.
as a specialty, W. G. A. Bonwill, 84.
clinical instruction in, W. G. A. Bonwill, 84.
fulcrums in, W. G. A. Bonwill, 12.
physiology of tooth movement in, S. H. Guilford, 395.
valuation of, W. G. A. Bonwill, 12.
when to begin, W. G. A. Bonwlll, 10.
Regulators and methods of correcting irregularities, W. G. A. Bonwill, 10, 76.
Re-inventions, C. J. Essig 165.
Relations of dentistry and medicine, J. Taft, 191.
Removable bridge-work, T. S. Waters, 197.
E. Parmly Brown, 677.
J. M. Edmunds, 655.
H. A. Parr, 238.
Pinney, 680.
C. S. Stockton, 679, 684.
T. S. Waters, 197, 238, 681.
R. B. Winder, 238.
clinic, T. S. Waters, 237.
entire dentures, T. S. Waters, 238.
control of wearer, T. S. Waters, 199.
patents on, T. S. Waters, 190.
canal fillings, W. H. Atkinson, 228.
W. H. Dwinelle, 230.
L. A. Faught, 286.
Jos. Head, 224.
S. G. Perry, 224.
James Truman, 231, 285.
root fillings, why? L. A. Faught, 286.
Jos. Head, 280.
James Truman, 285.
retainers, S. H. Guilford, 405.
splint in implantation, B. A. R. Ottolengui, 348.
Removal of irritants in treatment of pyorrhœa alveolaris, Geo. S. Allan, 721.
of prematurely erupted teeth, W. H. Atkinson, 620.
Repairing bridge-work, W. H. Dwinelle, 232.
T. S. Waters, 681.
Reparative action in dentine, R. R. Andrews, 169.
of pulps, R. R. Andrews, 169.
construction in regulating, S. H. Guilford, 399.
Repetition, necessity for, S. G. Perry, 65.
Replantation, H. S. Colding, 127.
J. W. Ivory, 97.
Reply to *Cosmos* on trade journalism, (editorial), 686.
"Report to the Board of Public Instruction in Paris," by Dr. Kuhn, notice of, 117.
Report of Proceedings of the American Academy of Dental Science, 158, 215.
Massachusetts Dental Society, 405, 470.
New Jersey State Dental Society, 37, 94, 539, 611, 673, 739.
Odontological Society of Pennsylvania, 32, 91, 163, 222, 277, 352, 421, 486.
Pennsylvania State Dental Society, 528, 600, 658, 734.
Philadelphia County Dental Society, 293.
Representative caste, the, S. B. Palmer, 384.
Reputation and real merit, S. B. Palmer, 385.
Resistance-box, the, H. E. Roberts, 710.
machine, White's, C. S. Beck, 734.
H. E. Roberts, 710.
Resistive action in the tooth, W. X. Sudduth, 352.
power of the tooth, Geo. S. Allan, 130.
C. N. Peirce, 360.
Resolution of thanks to Dr. Bonwill for gifts to the profession, W. X. Sudduth, 499.
Restoration of shapes of teeth, S. G. Perry, 66.
Retaining appliances, H. A. Baker, 418.
S. H. Guilford, 403.
fixtures in implantation, B. A. R. Ottolengui, 338.
implanted teeth, B. A. R. Ottolengui, 348.
pits, J. Morgan Howe, 697.
plugs used in regulating, J. N. Farrar, 264.
points in proximate surfaces, S. G. Perry, 140.
wire in regulating, E. H. Angle, 327.
Rete Malpighi, the resulting products of, W. X. Sudduth, 540.
Retention, mode of, in implantation, Fr. Abbott, 128.
of deciduous teeth, S. A. White, 124.

Reticulum of living matter in tooth substance, George S. Allan, 133.
Retrograde metamorphosis, M. L. Rhein, 619.
in pulp tissue, James Truman, 207.
of tissues (Abbott on), George S. Allan, 134.
Reverence for the natural teeth, S. G. Perry, 25.
Revitalization of peridental membrane, E. C. Kirk, 95.
Rhein, M. L., on Dr. Allan's paper on "Pyorrhœa Alveolaris," 744.
on Dr. Newton's paper on "The Teeth as a Factor in Diagnosis," 618.
on bridge-work, 683.
on pyorrhœa alveolaris, 744.
Rheumatism and hip-joint disease of dental origin, Dr. Dickie, 666.
Rheumatoid arthritis and the teeth, R. C. Newton, 581.
Rickets, causes of, R. C. Newton, 581.
Hutchinson's teeth in, R. C. Newton, 576.
first dentition in, R. C. Newton, 577.
Ridge, atrophied, implantation in, G. L. Curtis, 86.
Right-angle attachment to dental engines, W. G. A. Bonwill, 492.
plugger for electric or mechanical mallet, W. G. A. Bonwill, 490.
Rise and progress of American dentistry, W. Storer How, 466.
of dentistry, the, W. H. Atkinson, 461.
Roberts, H. E., on "Electricity and its Application in Dentistry," 703.
Robertson, Wm. (1835), "A Practical Treatise on the Human Teeth," R. R. Andrews, 512.
Rodent ulcers, appearance of, L. A. Faught, 297.
location of, L. A. Faught, 297.
Rôle of micro-organisms in pyorrhœa alveolaris, George S. Allan, 720.
Rollin's tubular knife in implantation, B. A. R. Ottolengui, 338.
"Room at the top," S. B. Palmer, 384.
Root, G. F., on "Preparation and Filling of Root Canals," 584.
on bichloride of mercury, 587.
Root, adjustment of, in implantation, G. L. Curtis, 87.
preparation of, for crowning, Theodore F. Chupein, 582.
-canal filling, A. G. Bennett, 288.
A. C. Boice, 287.
W. G. A. Bonwill, 287.
H. S. Colding, 127.
G. L. Curtis, 86.
W. H. Dwinelle, 230.
L. A. Faught, 286.
C. E. Francis, 228.
Joseph Head, 277, 525.
Sid Holland, 127.
H. H. Johnson, 127.
E. C. Kirk, 292.
Root-canal filing, W. H. Morgan, 126.
J. D. Patterson, 286.
H. C. Register, 289.
G. F. Root, 584.
Ambler Tees, 291.
W. H. Trueman, 456.
James Truman, 213, 285.
Van Worst's formula, 372.
drilling, W. H. Morgan, 126.
earlier methods of treatment, James Truman, 205.
enlarging, A. G. Bennett, 289.
filling for implantation, G. L. Curtis, 86.
immediate filling, H. C. Register, 289.
number of, Joseph Head, 277.
preparation of, G. F. Root, 584.
treatment of, A. G. Bennett, 289.
W. H. Dwinelle, 230.
C. E. Francis, 288.
unfilled, W. G. A. Bonwill, 287.
when ready to fill, James Truman, 212.
filling, choice of material, Wm. H. Trueman, 456.
materials, base plate gutta-percha, J. D. Patterson, 286.
carbolized cosmoline, Joseph Head, 281.
J. L. Patterson, 286.
James Truman, 285.
cement, Joseph Head, 279.
cotton, A. G. Bennett, 288.
W. G. A. Bonwlll, 288.
L. A. Faught, 286.
Joseph Head, 277.
J. D. Patterson, 286.
James Truman, 284.
and sandarach, J. D. Patterson, 386.
Gilbert's patent, H. S. Golding, 127.
gold, A. G. Bennett, 289.
A. C. Boice, 287.
Joseph Head, 278.
James Truman, 284.
gutta-percha, A. G. Bennett, 289.
A. C. Boice, 287.
J. D. Patterson, 286.
oxychloride of zinc, A. G. Bennett, 289.
J. D. Patterson, 286.
James Truman, 285.
sulphate of zinc, A. C. Boice, 287.
tin, James Truman, 285.
white gutta-percha, J. D. Patterson, 286.
Roots, badly decayed, to extract, Dr. Preston, 162.
"Rose-red," W. H. Atkinson, 105.
Rose-water and mercuric bichloride, George S. Allan, 253.
Rotation of central incisors, E. H. Angle, 328.

Roughness of neck of tooth, James Truman, 741.
in pyorrhœa alveolaris, George S. Allan, 720, 746.
Rounded joints for forceps, V. C. Pond, 159.
Routinist, the, A. G. Bennett, 591.
Rowan, Edward, exhibit of, 237.
Royalties of the International Tooth Crown Company, J. N. Crouse, 480.
Rubber and cheap dentistry, J. M. Edmunds, 655.
dam and arsenical ulceration, L. A. Faught, 295.
and pyorrhæa alveolaris, James Truman, 742.
arsenic in, L. A. Faught, 296.
clamps, W. H. Rollins, 186.
precautions in use of, L. A. Faught, 295.
plate and necrosis of the palate, W. D. Babcock, 464.
and catarrh, W. D. Babcock, 465.
Russell, J. W., "Copper Amalgam," 651.

### S.

Saddle-shaped jaws, R. C. Newton, 579.
James Truman, 611.
Safe dose of arsenious acid, L. A. Faught, 294.
Saliva a factor in caries, C. N. Peirce, 333.
W. X. Sudduth, 353.
Salivary calculus and caries, C. N. Peirce, 335.
tartar and pyorrhœa alveolaris, Geo. S. Allan, 717.
Salter on defects in the enamel, 514.
Sandarach tampon in oral wounds, Thomas Fillebrown, 217.
varnish and cotton as root filling, W. B. Miller, 609.
Sarcolactic acid, conversion of, Geo. S. Allan, 135.
Saw-frame, a new, C. P. Wilson, 162.
Scar tissue, W. H. Atkinson, 49.
Scavengers for bacteria, Geo. S. Allan, 131.
Schmidt, Professor, on diseases of the eye and the teeth, A. P. Brubaker, 641.
School of dentistry, Meharry Central Tennessee College, commencement of, 304.
Sciatic plexuses, neuralgia of, from dental irritation, A. P. Brubaker, 644.
Science, progress made in, A. G. Bennett, 590.
scientific advances in, C. C. Barker, 257.
Scientific aspect in implantation, G. L. Curtis, 85.
research, W. X. Sudduth, 111.
Screw-power in regulating, S. H. Guilford, 400.
press for plates, E. G. Leech, 420.
Scrofulous diathesis and implantation, E. C. Kirk, 96.
teeth, Dr. Gibney, 577.
Scurvy, the teeth in, R. C. Newton, 578.
Seabury & Johnson's exhibit, 108, 236.
Sea-horse teeth, Hayhurst, 102.
Searle, Dr. F., illness of (editorial), 123.
memorial resolutions, 437.
Second-grade dentists, S. B. Palmer, 384.
wisdom tooth, a case of, C. R. Jefferis, 92.
Secondary abscess, James Truman, 423.
decay, S. B. Palmer, 389.
dentine, W. H. Dwinelle, 229, 309.
deposition of, T. H. Parramore, 393.
development of, W. X. Sudduth, 352.
encroachments of, on pulp, W. H. Trueman, 449.
inducing deposits of, T. H. Parramore, 393.
production of, W. S. Elliott, 476.
Secretions, abnormal, effects of, on the teeth, W. X. Sudduth, 354.
of the mouth, quality of, C. N. Peirce, 333.
See, A. W., & Co's exhibit, 108.
"Seeing is believing," S. B. Palmer, 386.
Seiler's spray, 192.
Selection of bicuspids for implantation, B. A. R. Ottolengui, 341.
of teeth for implantation, B. A. R. Ottolengui, 338.
Self-cleansing bridge-work, T. S. Waters, 168.
Semi-decalcified dentine, zone of, Geo. S. Allan, 131.
in artificial caries, Geo. S. Allan, 132.
Senile dentine, James Truman, 170, 363.
Sensation, Geo. W. Whitefield, 731.
causation of, Geo. W. Whitefield, 731.
in dentine, W. X. Sudduth, 545.
Sensibility of dental tissues, C. N. Peirce, 167.
Sensitive cavities, copper amalgam for, J. W. Russell, 654.
dentine, S. B. Palmer, 389.
and its control, T. E. Weeks, 316.
teeth, filling of, S. B. Palmer, 388.
and Bright's disease, R. C. Newton, 621.
Sensitiveness in devitalized pulp, W. H. Trueman, 455.
Sensory paralysis, C. A. Brackett, 220.
Separationists, the, Dr. Flagg, 172.
Separation of the teeth, J. L. Eisenbrey, 178.
Separators, J. N. Crouse, 175.
S. H. Guilford, 238.
J. G. Morey, 238.
S. G. Perry, 143.
adjustment of, S. G. Perry, 139.
improved, W. A. Woodward, 89.
spreading with, S. G. Perry, 70.
Separator bar for cuspids and bicuspids, W. A. Woodward, 90.
for short teeth, W. A. Woodward, 90.
Septa of alveolar process, S. H. Guilford, 395.

Septic influences in implantation, G. L. Curtis, 86.
of devitalized dental fibres in implantation, G. L. Curtis, 88.
poison, C. C. Barker, 263.
Septicæmia after implantation, E. C. Kirk, 421.
case of, E. C. Kirk, 367.
Septum, thickening of, S. H. Guilford, 152.
Serous exudation from gums, and neck-caries, W. D. Miller, 311.
Serumal secretions, W. X. Sudduth, 178.
tartar, James Truman, 741.
in pyorrhœa alveolaris, George S. Allan, 716.
M. L. Rhein, 744.
W. X. Sudduth, 743.
Serumic calculus, character of, George S. Allan, 718, 747.
Sexton, Dr., on aural disease and the teeth, A. P. Brubaker, 641.
on dead teeth, A. P. Brubaker, 641.
on the importance of a knowledge of oral surgery for medical students, 575.
Sexual powers of protozoa, W. X. Sudduth, 542.
Shapes of beaks in new forceps, V. C. Pond, 159.
of teeth, restoration of, S. G. Perry, 66, 69.
Shaping capped teeth in bridge-work, J. M. Edmunds, 680.
cavities in proximate surfaces, S. G. Perry, 139.
Sharp stick in pulp extirpation, G. F. Root, 584.
Shellaced disk, W. G. A. Bonwill, 492.
Shell-caps for deformed incisors, L. C. Taylor, 647.
Shock in relation to dental operation, James Truman, 1.
Dr. Thomas, 33.
classification of, James Truman, 3.
definition of, G. V. Black, 1.
factors in, James Truman, 3.
from anæsthetics, James Truman, 8.
peculiar features, S. H. Guilford, 36.
secondary effects of, James Truman, 2.
subjective side, Theodore F. Chupein, 32.
L. A. Faught, 35.
S. H. Guilford, 35.
C. N. Peirce, 34.
James Truman, 7.
under anæesthetics, L. A. Faught, 34.
Dr. Thomas, 33.
James Truman, 8.
Short cuts (editorial), W. C. B., 241.
sittings, A. G. Bennett, 36.
C. N. Peirce, 34.
W. X. Sudduth, 32.
Dr. Thomas, 33.
teeth, separator bar for, W. A. Woodward, 90.
Shrady, Dr., on the importance of surgical diseases of the teeth, 575.
Shredded tin (editorial), W. C. B., 242.
Sibley's, Gideon, exhibit, 108, 236.
Sickness during calcification of the teeth, effects of, R. C. Newton, 578.
Side tracks (editorial), W. C. B., 241.
Significance of salivary calculus, C. N. Peirce, 335.
Silex: what is it? W. H. Atkinson, 105.
Silico-fluoride of soda, W. X. Sudduth, 94, 315.
in pyorrhœa alveolaris, W. X. Sudduth, 744.
Silk and rubber ligatures in regulating, 325.
Silk travelling-cap as occipital bandage in regulating, E. H. Angle, 326.
Silver disk as cap for exposed pulps, S. H. Guilford, 473.
proportion of, in amalgams, L. A. Faught, 607.
James S. King, 606.
Similarity in development of hair and teeth, W. X. Sudduth, 539.
Simple elements in nature, S. B. Palmer, 383.
Single rooted teeth, treatment, canals of, Joseph Head, 277.
Sixth-year molars, extraction of, C. N. Peirce on, A. P. Brubaker, 642.
value of, W. G. A. Bonwill, 11.
Skeleton plates, S. A. White, 701.
Skin-diseases, study of, by dental students, E. H. Smith, 321.
Skin, section of, microscopic appearance, W. X. Sudduth, 544.
Slides (Dr. Abbott's), George S. Allan, 134.
Sliding bar in separator, W. A. Woodward, 89.
Sloughing from ether spray, B. A. R. Ottolengui, 338.
out of stitches, in operation for fissure of soft palate, James E. Garretson, 349.
Small shank pluggers, S. G. Perry, 140.
Smith, Eugene H., on dental education, 319.
on implantation, 101.
Snow & Lewis's automatic mallet, E. C. Kirk, 520.
Social elements in success, S. B. Palmer, 391.
Society, dental, value of, A. G. Bennett, 590.
libraries, W. Storer How, 468.
membership, S. B. Palmer, 391.
Socket forming for implantation, G. L. Curtis, 87.
for lateral incisor implantation, B. A. R. Ottolengui, 339.
for lower bicuspid implantation, precautions, B. A. R. Ottolengui, 347.
for superior central incisor implantation, precautions, B. A. R. Ottolengui, 338.
Soft foil, advantages of, S. G. Perry, 76.
and cohesive, in connection, E. B. Davis, 274.
James Truman, 276.
in contour work, E. B. Davis, 274.

Soft foil and progressive dentistry, E. B. Davis, 271.
as a time-saver, E. B. Davis, 274.
as a tooth-saver, E. B. Davis, 273.
at cervical borders, J. A. Libbey, 539.
change to cohesive, James Truman, 276.
experts in, E. B. Davis, 273.
for lining cavities, E. B. Davis, 274.
objections to, James Truman, 276.
when to use, E. B. Davis, 274.
gold, S. G. Perry, 141, 147.
and cohesive, use of both, E. B. Davis, 276.
in large masses, S. G. Perry, 147.
rubber clasps, E. A. Bogue, 185.
disks, W. G. A. Bonwill, 492.
Soldering collars to bands in regulating, E. H. Angle, 325.
small pieces of gold work, J. Bond Littig, 698.
Some phases of dental iiterature, W. Storer How, 466.
Source of supply of platinum, (editorial), 431.
Southern Medical College, Dental Department, commencement of, 304.
Spasm of voluntary muscles of dental origin, A. P. Brubaker, 643.
Spasmodic neuralgia, Thomas Fillebrown, 216.
Speakman, W. M., exhibit, 237.
Special schools of dentistry, E. H. Smith, 322.
offer to students (editorial), 117.
Specialists, S. B. Palmer, 385.
Specialized cells of tooth structure, C. N. Peirce, 330.
Specialties, W. Storer How, 466.
S. B. Palmer, 385.
Specimens, microscopic (Abbott's), Geo. S. Allan, 134.
Spence metal in the hydraulic press, E. A. Bogue, 391.
Sphero-bacteria, C. C. Barker, 258.
Spinal cord tissue, microscopic appearance of, W. X. Sudduth, 544.
Spiral spring in regulating, W. G. A. Bonwill, 10, 491.
Spirilla, C. C. Barker, 258.
Spiro-bacteria, C. C. Barker, 258.
Splints in implantation, B. A. R. Ottolengui, 338.
Split posts in bridge-work, W. H. Dwinelle, 232.
T. S. Waters, 201.
Sponge gold, J. N. Crouse, 175.
Sponge grafting and implantation, J. Taft, 28.
for exposed pulps, T. H. Parramore, 394.
sterilized, for pulp capping, W. H. Atkinson, 748.
W. J. Elliott, 728.
T. H. Parramore, 393.
Spontaneous generation, W. X. Sudduth, 51.
Sporulation of protozoa, W. X. Sudduth, 542, 545.
Spray apparatus, H. C. Register, 93.
syringe for treatment of diseases of the antrum, J. N. Farrar, 457.
Spring catch in bridge-work, T. S. Waters, 199.
Squaring temporary teeth in regulating, W. G. A. Bonwill, 81.
St. Louis College of Physicians and Surgeons, dental department, commencement of, 304.
Dental Society, 123.
St. Vitus's dance and the teeth, S. C. G. Watkins, 617.
Stamping plates by hydraulic press, E. A. Bogue, 391.
Standards, a comparison of (editorial), 305.
for graduation, raising the (editorial), 300.
of admission to dental colleges, A. G. Bennett, 589.
Standing of dental diplomas (editorial), 306.
room at the bottom, S. B. Palmer, 384.
Staple, use of, in implantation, G. L. Curtis, 87, 108.
Dr. Smith, 101.
Starr, R. N., clinic in porcelain inlays, 238.
Starr's "Hygiene of the Nursery," notice of, 434.
removable bridge-work, J. M. Edmunds, 656.
Starting-point, the, S. B. Palmer, 384.
State and national societies, A. G. Bennett, 590.
Statistics in proof of assertion, L. A. Faught, 535.
Jos. Head, 527.
J. R. C. Ward, 535.
of literature, Dr. Faught on, A. G. Bennett, 592.
Status of dentistry in America, Dr. Kuhn, 117.
Stay-plates, W. G. A. Bonwill, 12.
gutta-percha, in regulating, W. G. A. Bonwill, 77.
Stentor, cœruleous, reproduction of, W. X. Sudduth, 545.
Stent's composition, E. A. Bogue, 391.
Sterilizing instruments, James Truman, 211.
root-canals, Jos. Head, 280.
Sterilized sponge as pulp capping, W. H. Atkinson, 748.
W. S. Elliott, 728.
T. H. Parramore, 393.
in lesions of the soft tissues, W. S. Elliott, 728.
Stevens's filling instruments, J. A. Libbey, 538.
Stiffened Jaw, case of, E. B. Quick, 305.
Stock in trade, S. B. Palmer, 386.
Stockton, C. S., on bridge-work, 679.

Stockton's teeth, W. H. Dwinelle, 106.
Dr. Fowler, 104.
J. Hayhurst, 102.
James Truman, 104.
Storage battery, H. E. Roberts, 707.
Stored radiancy, W. H. Atkinson, 38, 47, 554, 620.
Stowell's Atlas (editorial) 117.
Strangulation of pulp, James S. King, 650.
in regulating, S. H. Guilford, 390.
Structural arrangement of cementum, C. N. Peirce, 332.
of dentine, C. N. Peirce, 332.
of enamel, C. N. Peirce, 330.
defects of dentine C. N. Peirce, 332.
of enamel, C. N. Peirce, 331.
Studies in etiology, E. M. Hunter, 261.
Style in dental literature, A. G. Bennett, 594.
Sub-alveolar forceps, Ambler Tees, 497.
Sub-carbonate of potash in glycerine, effects of, on tooth substance, F. J. Bennett, 196.
Subscription bills (editorial), 376.
Success in dental practice, S. B. Palmer, 383.
law of, S. B. Palmer, 388.
ultimate, of implantation, G. L. Curtis, 85.
Sudduth's, W. X., lantern exhibit, 45, 166, 542.
Sudduth, W. X., on antisepsis, 94.
on beginnings of life, 51.
on bridge and crown patents, 496.
on Dr. Allan's paper on "Pyorrhœa Alveolaris," 743.
on implantation, 97.
on the individuality of the biological cell, 539.
on patents, 495.
on psychical influences, 32.
on pyorrhœa alveolaris, 743.
reply to Dr. Heitzmann, on the cellular theory, 556.
Suit against J. N. Crouse, 254, 764.
H. A. Parr, 254.
Sulphate of quinia as an antiseptic, James Truman, 742.
in pyorrhœa alveolaris, James Truman, 743.
of zinc as root-canal filling, A. C. Boice, 287.
Sulpho-carbolic acid, Laplace, 128.
Sulphuric acid in pyorrhœa alveolaris, Geo. S. Allan, 722.
W. X. Sudduth, 744.
James Truman, 743.
Sunshine for dentists, L. A. Fuaght, 35.
Superior merits of Bonwill's method of packing foil, E. C. Kirk, 523, 536.
Supernumerary teeth, Joseph Leidy, 313.
Superficial chronic pulpitis, James Truman, 207.
Surgeons, American, skill of (editorial), 110.
Survival of the fittest, E. A. Bogue, 312.
S. G. Perry, 149.
Suspension engine, W. S. Elliot, 729.
Ludwig's, run by electric motor, 765.
Swamp angels (editorial), W. C. B., 242.
Switch-board, S. B. Palmer, 390.
Switzerland, patent laws of, L. C. Bryan, 246.
Syllabus of lectures, J. Taft, 192.
Symptoms of collapse, James Truman, 3.
of pyorrhœa alveolaris, George S. Allan, 713.
Syncope, E. C. Kirk, 99.
Synthetic work of Dr. W. D. Miller, George S. Allan, 131.
Syphilis and the teeth, C. N. Peirce, 612.
James Truman, 611.
and implantation, E. C. Kirk, 95.
Syracuse reunion of New York District Dental Societies, 57.
Systemic abnormity, causes of, C. N. Peirce, 334.
effects of, on tooth structure, C. N. Peirce, 334.
conditions and influences, in pyorrhœa alveolaris, George S. Allan, 719.
effects of, on the teeth, C. N. Peirce, 360.
disturbance and the teeth, C. N. Peirce, 514.

T.

Tabulate your experiments, S. B. Palmer, 385.
Taking off the enamel, S. G. Perry, 71.
Talbot spiral spring, W. G. A. Bonwill, 19.
Tannin and glycerine paste, W. H. Atkinson, 177.
Tape in wedging, S. G. Perry, 143.
Tartar and pyorrhœa alveolaris, W. X. Sudduth, 743.
James Truman, 742.
as cause of pyorrhœa alveolaris, George S. Allan, 716.
M. L. Rhein, 744.
James Truman, 740.
on dog's teeth, S. C. G. Watkins, 42.
the prime cause of pyorrhœa alveolaris, George S. Allan, 716, 719, 746.
Taylor, L. C., two interesting cases, 647.
Tees, Ambler, on cotton as root-canal filling, 291.
on patents, 497.
Teeth, the, S. H. Guilford, 396.
as factors in disease, Dr. Main, 127.
in length of life, J. Y. Crawford, 127.
association of, with other organs, A. P. Brubaker, 639.
changes in character of, Geo. S. Allan, 170.
in color of, C. N. Peirce, 167.
in density of, C. N. Peirce, 168.
choice of, in implantation, G. L. Curtis, 86.
compressing, in sockets in regulating, E. H. Angle, 327.

Teeth, connection of, with general arterial and nerve systems, C. N. Peirce, 167.
development of, as standard of physical capacity, R. C. Newton, 581.
elongation of, in regulating, E. H. Angle, 327.
from battle-fields for implantation, L. C. Bryan, 246.
from dissecting rooms for implantation, Wetzel, 246.
influence of, on nutrition, A. P. Brubaker, 639.
lost by pyorrhœa alveolaris, comparative estimate of number of, Geo. S. Allan, 713, 745.
James Truman, 739.
made from glacial epoch boulder, Wm. H. Atkinson, 105.
of early races, E. A. Bogue, 312.
of emigrants, C. S. Stockton, 44.
of New-Englanders, J. T. Codman, 44.
of old age, Geo. S. Allan, 171.
of the sick, E. C. Briggs, 633.
the rôle of, A. P. Brubaker, 639.
Telescoping bridge-work, W. H. Dwinelle, 232.
caps in bridge-work, C. C. Carroll, 685.
J. M. Edmunds, 656.
Temperament, influence of, in implantation, G. L. Curtis, 87.
Temporary teeth as fulcrums in regulating, W. G. A. Bonwill, 76.
preservation of, W. G. A. Bonwill, 10.
slots cut in, in regulating, W. G. A. Bonwill, 77.
Ten don'ts, D. M. C., 252.
Tenth International Medical Congress (editorial), 375.
dental section of (editorial), 431.
Test for iron in copper amalgam, J. W. Russell, 653.
for leakage in root-canal fillings, Jos. Head, 370.
Tested antiseptics, James Truman, 211.
Tetanin, Dr. Rosenboch, 263.
Tetanotoxin, Dr. Rosenboch, 263.
Tetanus bacilli, Dr. Brieger, 263.
Text-books, dental literature of, A. G. Bennett, 589.
Thanks (editorial), 115.
to Dr. W. G. A. Bonwill, 499.
votes of, 109.
The advantages of cotton as a root-Canal filling, Joseph Head, 277.
discussion, 284.
The American Dental Trade Association (editorial), 429.
The antipyrin habit (editorial), 500.
The basis substance in calcified products (editorial), 239.
The Bonwill method of packing gold foil, E. C. Kirk, 519.
discussion, E. C. Kirk, 536.
The border land of calcification, R. R. Andrews, 193.
The Brown-Séquard elixir (editorial), 622
The cell, W. H. Atkinson, 47, 553.
C. Heitzmann, 47, 553.
B. A. R. Ottolengui, 48, 51.
W. X. Sudduth, 46, 541.
James Truman, 50.
The corpuscle, W. H. Atkinson, 46, 50.
The dentist born, not made, W. H. Atkinson, 461.
The etiology of dental caries, Geo. S. Allan, 129.
discussion, 166.
The French Congress (editorial), 374, 500.
The germ theory (editorial), 179.
The implantation of teeth, G. L. Curtis, 85.
discussion, G. L. Curtis, 194.
The Journal, position of (editorial), 566.
The ministry a learned profession, why? E. H. Smith, 320.
The Odontological Society of Pennsylvania, (editorial), 688.
The phonograph in the First District Dental Society, New York, 754.
The pulp and treatment of pulp-canals, James Truman, 203.
discussion, James Truman, 222.
The reason why, J. N. Crouse, 413.
L. D. Shepard, 412.
The seat of war, root-canals, A. G. Bennett, 288.
The teeth as a factor in diagnosis, Richard C. Newton, 575.
discussion, Richard C. Newton, 611.
The treatment of proximate surfaces, Safford G. Perry, 65, 139.
discussion, Safford G. Perry, 171.
The use of the matrix with proximate amalgam fillings, F. S. Bassett, 600.
discussion, F. S. Bassett, 602.
The vascularity of tissue (editorial), 309.
The work of the future, removable bridge-work, T. S. Waters, 203.
The young practitioner, J. C. White, 658.
discussion, J. C. White, 661.
Theory, the germ, George S. Allan, 130.
protoplasmic, George S. Allan, 130.
of caries, Dr. Abbott's, George S. Allan, 134.
Therapeutic treatment for pulp devitalization, W. H. Trueman, 447.
Therapy of the future, the, James Truman, 93.
Thermal changes, S. B. Palmer, 388.
and capped pulps, C. S. Beck, 670.
and the pulp, W. H. Trueman, 449.
and the tooth, H. C. Register, 532.
in metallic fillings, James S. King, 650.
influence in pulp irritation, James Truman, 204.
Thick rubber dam, D. M. C., 253.
Thickening of alveolar septum, S. H. Guilford, 152.
Thin, scaly, smooth calculus, in pyorrhœa alveolaris, George S. Allan, 718.

Thomas on nitrous oxide, 34.
on shock, 33.
Thompson, A. H., on cements, 439.
on gouty teeth, 577.
Thorough finishing, T. H. Parramore, 415.
Thoroughness, W. H. Atkinson, 460.
F. S. Bassett, 601.
Thousand dollars reward, W. H. Dwinelle, 176.
Timme, C. A., clinic, 107.
Tin, antiseptic qualities of, S. B. Palmer, 389.
as root-canal filling, James Truman, 285.
value of, in amalgams, James S. King, 606.
Tipping forward of molar teeth in regulating, E. H. Angle, 324.
Tissue builders, W. X. Sudduth, 542.
of pericementum, characteristics of, S. H. Guilford, 397.
Tissues of lower animals, photo-micrographs, W. X. Sudduth, 45.
individuality of, C. N. Peirce, 330.
of tooth structure, C. N. Peirce, 330.
To prevent blackened flakes, W. C. Browne, 637.
Toasts at dinner to Dr. Atkinson, 249.
Tobacco an antiseptic, E. C. Kirk, 93.
Todd and Bowman on the enamel cord, 553.
Toleration of nature, W. H. Trueman, 452.
Toll-gates (editorial), W. C. B., 243.
Tonic applications in pyorrhœa alveolaris, George S. Allan, 723.
Tonsils, enlarged, and mouth-breathing, R. C. Newton, 579.
Tooth Crown Company, claims of, 376.
patents of, 478.
suits of, 254, 764.
Tooth, condition of, after pulp extirpation, H. C. Register, 290.
decay, H. C. Register, 530.
destruction, acids in, George S. Allan, 130.
factors promoting, George S. Allan, 129.
development, C. N. Peirce, 330.
H. C. Register, 530.
microscopic appearance of, W. X. Sudduth, 550.
discoloration, James Truman, 207.
environment, H. C. Register, 530.
germs, origin of, C. N. Peirce, 330.
nutrition, C. N. Peirce, 361.
James Truman, 362.
pullers, J. C. Green, 661.
*vs.* tooth savers, J. C. White, 661.
rotation, Adair's method of, 700.
softening with copper amalgam, J. W. Russell, 653.
structure, cavities in, George S. Allan, 130.
changes in, R. R. Andrews, 169.
substance, living matter in, Fr. Abbott, 133.
Topical Index (editorial), 55.
thanks for, L. C. Bryan, 189, 246.
Touters (editorial), W. C. B., 242.
Townsend, H., on arsenious acid, 298.
amalgam, I. J. Weatherby, 411.
Toxæmia (editorial), 180.
Traction-bar in regulating, E. H. Angle, 325.
Trade, influence of, 184.
journalism (editorial), 686.
final reply (editorial), 756.
Training for students, E. H. Smith, 319.
Transmutation of basis substance, George S. Allan, 137.
Transparent zone, the, W. X. Sudduth, 357.
James Truman, 356.
Transplantation, W. X. Sudduth, 191.
Traumatic injury of pulp, W. H. Trueman, 449.
irritation, W. H. Trueman, 455.
Travelling expenses of delegates, W. E. Magill, 633.
Treason, S. B. Palmer, 387.
Treatment of exposed pulps with a view to preservation, James S. King, 649.
discussion, 668.
Treatment of proximate surfaces, Safford G. Perry, 65, 139.
discussion, 171.
"Treatment of Pulpless and Abscessed Teeth," by George Cunningham, notice of, 117.
Treatment of arsenical ulceration, L. A. Faught, 297.
of children, S. B. Palmer, 386.
of exposed pulps, antiseptics in, T. H. Parramore, 373.
of pathological conditions of pulp, James Truman, 204.
of pulp-canals, Jas. Truman, 203, 210.
Trifacial nerve, the, and the teeth, A. P. Brubaker, 639.
Trigeminal neuralgia, M. L. Rhein, 619.
Trimming our curriculum, E. H. Smith, 321.
the cervical edges for amalgam filling, F. S. Bassett, 604.
C. N. Kratzer, 604.
Trismus, case of, E. L. Clifford, 761.
Trophic centres, abnormal condition of, E. C. Kirk, 96.
disorders of dental origin, A. P. Brubaker, 643.
Truman, James, on antisepsis, 93.
on diet, 39.
on Dr. Allan's paper on "Pyorrhœa Alveolaris," 739.
on Dr. Codman's paper on "Enforced Climate and Diet," etc., 39.
on Dr. Newton's paper on "The Teeth as a Factor," etc., 611.
on galvanic current, 366.
on micro-organisms, 93.
on nitrous oxide, 9.
on patents, 497.
on practical operations, 7.
on pyorrhœa alveolaris, 739.
on "Shock in Relation to Dental Operations," 1.

Trueman, Wm. H. on "The Pulp and Treatment of Pulp-Canals," 203.
on cotton as root-canal filling, 372.
on intentional devitalization of the dental pulp, 447.
Tuberculosis and implantation, E. C. Kirk, 96.
Tuberculous teeth, Dr. Gibney, 577.
C. N. Peirce, 615.
Tubuli, calcific deposits in, George S. Allan, 71.
R. R. Andrews, 169.
G. V. Black, 169.
calco-spherites in, R. R. Andrews, 169.
condition of, after pulp devitalization, James Truman, 231.
in senile dentine, James Truman, 170.
mercury in, George S. Allan, 171.
Tumor in nerve tissue, Thomas Fillebrown, 218.
Turgidity of gum tissue as a cause of tooth-movement, S. H. Guilford, 155.
Two interesting cases, L. C. Taylor, 647.
Typal demands, Wm. H. Atkinson, 554.
W. X. Sudduth, 550.
Typhoid fever, mouth and teeth in, E. C. Briggs, 633.
Typical forms, W. H. Atkinson, 47.
Tyron, George S. Allan, 225.

U.

Ulceration, arsenious, L. A. Faught, 295.
of cornea and sclerotic and the teeth, A. P. Brubaker, 641.
Ultimate success of implantation, G. L. Curtis, 85.
E. C. Kirk, 96.
Underbite, abnormity of, W. G. A. Bonwill, 14.
normal, W. G. A. Bonwill, 13.
Undercuts in proximate surfaces, S. G. Perry, 140.
Unicellular bodies, W. X. Sudduth, 542.
Unilateral blindness and the teeth, A. P. Brubaker, 641.
defective power of accommodation, and the teeth, A. P. Brubaker, 641.
Union is strength, S. B. Palmer, 386.
of dentine and cementum fibres, C. N. Peirce, 333.
with alveolar process, E. C. Kirk, 95.
United brotherhood, a, S. B. Palmer, 386.
Uniting teeth by gold bar, M. L. Rhein, 380.
Universal separator, H. A. Parr, 238.
University, Columbian, Dental Department, commencement of, 303.
Howard, Dental Department, commencement of, 303.
of Iowa, Dental Department, commencement of, 303.
of Maryland, Dental Department, commencement of, 303.
of Tennessee, Dental Department, commencement of, 304.
University, Vanderbilt, Dental Department, commencement of, 304.
of Maryland and the National Association of Dental Examiners, correspondence of, F. J. S. Gorgas, 120, 122.
F. A. Levy, 60, 121.
Urine in pyorrhœa alveolaris, M. L. Rhein, 744.
Use and abuse of drug-habits (editorial), 500.
Use of the matrix with proximate amalgam fillings, F. S. Bassett, 600.
discussion, 602.
Use of dam in treatment of proximate surfaces, S. G. Perry, 139.
of electricity in dentistry, H. E. Roberts, 711.
Uterus, neuroses of, of dental origin, A. P. Brubaker, 643.
Utilitarian aspects (editorial), 110.
Utilizing cavities in regulating, W. G. A. Bonwill, 80.

V.

V-shaped jaws, R. C. Newton, 579.
Vacuum, a, and pulp vitality, G. W. Klump, 669.
a, and the pulp, James S. King, 669.
Value of a knowledge of the tissues, C. N. Peirce, 329.
of the anatomy of the maxillæ, B. A. R. Ottolengui, 336.
of a new idea (editorial), 111.
of essential oils, A. W. Harlan, 315.
of the pulp, W. H. Trueman, 453.
of the teeth, R. C. Newton, 578.
Vanderbilt University, Dental Department, commencement of, 304.
Variations from law, S. H. Guilford, 153.
in opinion, James Truman, 204.
Varied functions of the pericementum, S. H. Guilford, 398.
Varney pluggers, S. G. Perry, 142.
Varney's contour fillings, S. G. Perry, 69.
Varnish and exposed pulps, J. N. Farrar, 270.
as lining for cavities, S. B. Palmer, 389.
Vascular system of the pulp, S. H. Guilford, 396.
Vascularity of the alveolar process, S. H. Guilford, 395.
of tissue, S. H. Guilford, 309.
of tooth substance, Geo. S. Allan, 136.
Vaso-motor disorders of dental origin, A. P. Brubaker, 643.
nerves, action of, James Truman, 2.
excito-motor centre of (Goltz), James Truman, 3.
theory of Simon, James Truman, 3.
of Wagner, James Truman, 2.
Velocity of blood currents, Jos. Head, 282.
Velvet rubber dam, 236.
Venting root-canal fillings, Jos. Head, 280.

Verbal assertion *vs.* scientific fact, Jos. Head, 524.
Vessels in the inferior maxilla, location of, B. A. R. Ottolengui, 336.
in the superior maxilla, B. A. R. Ottolengui, 336.
Vices of conformation in enamel tissue, C. N. Peirce, 331.
Visceral disorders of dental origin, A. P. Brubaker, 643.
Vital action in caries (Abbott), George S. Allan, 134.
fluid of dental tissues, C. N. Peirce, 167.
Vitality, loss of, in capped pulps, J. S. King, 653.
of peridental membrane, George S. Allan, 723.
after extraction, E. C. Kirk, 95.
Vitiated secretions, correction of, C. N. Peirce, 335.
of antrum, E. C. Kirk, 423.
Victims to antipyrin (editorial), 500.
Volt, definition of, H. E. Roberts, 704.
Voluntary tooth movement resulting in excessive interdental spaces, S. H. Guilford, 149.
discussion, 176.
means for correction of, 156.
prevention of, 156.
Vulcanizer, zinc in, to prevent blackened flasks, W. C. Browne, 637.

W.

Walker–Younger trephine, B. A. R. Ottolengui, 342.
Walls of nerve-canal in inferior maxilla, B. A. R. Ottolengui, 336.
Waltham Emery Wheel Co.'s exhibit, 230.
Want of cleanliness, and caries, C. N. Peirce, 335.
Ward, J. R. C., on Dr. Head's paper on "Dental Assertion," 535.
Ward's electro-metallic plate exhibit, 108.
Warning, a note of, W. H. Dwinelle, 172.
Wart retaining-plugs, J. N. Farrar, 264.
Washing action of the fluids of the mouth, S. G. Perry, 139.
the hands, how to do it, B. A. R. Ottolengui, 337.
Waste products of bacterial life, George S. Allan, 130.
Water-tight antiseptic fillings, Joseph Head, 370.
Water-tight filling, Joseph Head, 278.
Waters, T. S., clinic, 107, 237.
donation to the profession, 200.
"Removable Bridge-Work," 197.
discussion, 232.
exhibit of bridge-work, 237.
on absorption of tissues, 198.
on bridge-work, 197, 681.
Watkins, S. C. G., clinic, 108.
"Watt," definition of, H. E. Roberts, 705.
Watt's crystal gold, E. T. Darby, 632.
S. G. Perry, 141.
Way to success, the, S. B. Palmer, 391.
Weariness, what is it? James Truman, 4.
Weather, the, and copper amalgam, F. E. Gilson, 675.
Webb's method of condensing gold, C. S. Beck, 538.
E. C. Kirk, 538.
Wedging a matrix, F. S. Bassett, 603.
W. B. Miller, 603.
Welch Dental Company's exhibit, 108.
alloy, C. S. Beck, 607.
Welding soft gold foil, W. G. A. Bonwill, 493.
Weston's non-irritant cement, James S. King, 649.
Wet moulding-sand as investment in soldering, J. Bond Littig, 698.
Weatherby, I. J., on sterilized sponge as pulp-capping, 476.
Wheeler, C. F., clinic, 107.
Where do all the graduates go? (editorial), 300.
White, J. C., on "The Young Practitioner," 658.
discussion, 661.
S. S., and the old Vulcanite Company, (editorial), 565.
& Co's dental exhibit, 108.
electric exhibit, 236.
White gutta-percha as root-canal filling, J. D. Patterson, 286.
zone in artificial caries, R. R. Andrews, 169.
in natural caries, R. R. Andrews, 169.
Whitefield, George W., on sensation, 731.
Whose ox is gored? W. G. A. Bonwill, 494.
Why? *vs.* how? (editorial), 752.
Wiggling bridge-work, J. M. Edmunds, 681.
W. Pinney, 680.
Wild on antiseptics in replantation, B. A. R. Ottolengui, 337.
Williams's, R. S., exhibit, 237.
Wilmington Manufacturing Company's exhibit, 236.
Winder, R. B., on diet, 46.
on removable bridges, 238.
Wire retaining-pegs, J. N. Farrar, 264.
Wisdom tooth, carious, and otalgia, E. C. Kirk, 664.
copper amalgam for, J. W. Russell, 654.
difficult eruption, case of, E. L. Clifford, 762.
and deafness, A. P. Brubaker, 642.
exostosed, and ear catarrh, E. C. Kirk, 664.
in implantation of molars, B. A. R. Ottolengui, 346.
influence of, J. Y. Crawford, 126.
Woakes on ear affections of dental origin, A. P. Brubaker, 642.
Wood creosote and morphine, J. N. Farrar, 271.
and zinc oxide in pulp preservation, James S. King, 649.

Wood screw in extraction of roots, Dr. Penton, 162.
Wood's fusible alloy, E. A. Bogue, 392.
Woodward, W. A., improved separator for incisors, cuspids, and bicuspids, 89.
  illuminating apparatus, 238.
  matrix, S. G. Perry, 146.
  separator, to adjust, 90.
Work of Odontological Society of Pennsylvania, C. J. Essig, 164.
Workings of the medulla oblongata, A. P. Brubaker, 646.
Worn-away teeth, restoration of, H. H. Edwards, 693.
Worden on aural diseases and the teeth, A. P. Brubaker, 641.
Writer, the, A. G. Bennett, 592.
  in the dental profession (editorial), 502.
Wry neck from dental irritation, A. P. Brubaker, 643.

Y.

Young practitioner, the, J. C. White, 658.
  discussion, 661.
Young members of the profession, encouragement of, B. H. Catching, 702.
  L. A. Faught, 662.
Young members of the profession, J. C. Green, 661.
  Sid Holland, 702.
  J. C. White, 660.
  S. A. White, 701.
Your correct name and address (editorial), 375.

Z.

Zinc cement, disintegration of, J. N. Farrar, 270.
    with exposed pulps, J. N. Farrar, 270.
  chloride as pulp-capping, S. H. Guilford, 473.
  in the vulcanizer to prevent blackened flasks, W. C. Browne, 637.
  oxychloride, James Truman, 213.
    as root-canal filling, Ambler Tees, 292.
  phosphate and exposed pulps, J. N. Farrar, 270.
  process, the, in making copper amalgam, J. W. Russell, 653.
  value of, in amalgams, James S. King, 606.
Zone of resistance, R. R. Andrews, 169.
  of semi-decalcified, Geo. S. Allan, 138.
  transparent, James Truman, 170.
Zymotic diseases, James Truman, 209.

### SUPPLEMENTARY INDEX OF AUTHORS.

Abbott, Frank, New York City, N. Y.
Abel, F. H., Erie, Pa.
Adam, R. B., Gainesville, Ga.
Alexander, J. H., Mystic River, Mass.
Allan, Geo. S., New York, N. Y.
Allport, W. W., Chicago, Ill.
Andrews, R. R., Cambridge, Mass.
Angle, Ed. H., Minneapolis, Minn.
Arnold, N. F., Rochester, N. Y.
Atkinson, W. H., New York, N. Y.

Babcock, Wm. D., Los Angeles, Cal.
Baker, H. A., Boston, Mass.
Banfield, F. E., Boston, Mass.
Barrett, W. C., Buffalo, N. Y.
Basset, Frank, Philadelphia, Pa.
Baumgardner, J. G., Philadelphia, Pa.
Beck, G. S., Wilkesbarre, Pa.
Bennett, A. G., Philadelphia, Pa.
Black, G. V., Chicago, Ill.
Bogue, E. A., New York, N. Y.
Boice, Alonzo C., Philadelphia, Pa.
Bonwill, W. G. A., Philadelphia, Pa.
Brackett, C. A., Newport, R. I.
Briggs, Ed. C., Boston, Mass.
Brown, E. Parmly, Flushing, L. I.
Brown, G. C., Elizabeth, N. J.
Browne, W. C., Atlanta, Ga.
Brubaker, A. P., Philadelphia, Pa.
Bryan, L. C., Basel, Switzerland.
Burne, A., Sydney, N. S. Wales.
Butler, C. S., Buffalo, N. Y.

Carpenter, L. D., Atlanta, Ga.
Carroll, C. C., Meadville, Pa.
Catching, B. H., Atlanta, Ga.
Cheney, G. F., St. Johnsbury, Vt.
Chewning, G. L., Fredericksburg, Va.
Chupein, T. F., Philadelphia, Pa.
Clapp, D. M., Boston, Mass.
Codman, J. T., Boston, Mass.
Colding, H. S., Savannah, Ga.
Conrad, Wm., St. Louis, Mo.
Cook, C. D., Brooklyn, N. Y.

Cooke, F. A., Boston, Mass.
Cornwall, B. C., St. Paul, Minn.
Crawford, J. Y., Nashville, Tenn.
Crouse, J. N., Chicago, Ill.
Cunninghan, G., Cambridge, Eng.
Curtis, G. L., Syracuse, N. Y.

Darby, E. T., Philadelphia, Pa.
Darby, F. B., Elmira, N. Y.
Davis, E. B., Concord, N. H.
Davis, L. L., Chicago, Ill.
De Lange, C. E., Philadelphia, Pa.
Dickey, S. J., Philadelphia, Pa.
Dixon, C. M., Philadelphia, Pa.
Dorrance, W. H., Ann Arbor, Mich.
Dubois, P., Paris, France.
Dwinelle, W. H., New York, N. Y.

Edmunds, J. M., New York, N. Y.
Edwards, H. H., Madrid, Spain.
Eisenbrey, J. L., Philadelphia, Pa.
Elliott, W. S., Hartford, Conn.
Elliott, W. St. G., London, Eng.
Essig, C. J., Philadelphia, Pa.

Farrar, J. N., New York, N. Y.
Faught, L. A., Philadelphia, Pa.,
Fergus, O., Glasgow, Scotland.
Fillebrown, T., Boston, Mass.
Fletcher, C. R., Sao Paulo, Brazil.
Francis, C. E., New York, N. Y.
Fundenberg, W. F., Pittsburg, Pa.

Garretson, J. E., Philadelphia, Pa.
Gerry, G. A., Lowell, Mass.
Gilbert, S. E., Philadelphia, Pa.
Gilson, F. E., Groton, Mass.
Gorgas, F. J. S., Baltimore, Md.
Green, J. C., Westchester, Pa.
Guilford, S. H., Philadelphia, Pa.
Gunn, C. B., Leavenworth, Kan.

Hamilton, J. C. M., Tyrone, Pa.
Harlan, A. W. Chicago, Ill.
Harrison, F., Sheffield, Eng.
Harrison, W., Brighton, Eng.
Harrower, A. B., Philadelphia, Pa.
Hayward, C. H., Peterborough, N. H.
Hayhurst, J., Lambertville, N. J.
Head, Joseph, Philadelphia, Pa.
Heitzmann, Carl, New York, N. Y.
Holland Sid, Atlanta, Ga.
Hopkins, C. E., Philadelphia, Pa.
Hopkins, E. E., Boston, Mass.
How, W. S., Philadelphia, Pa.
Howe, J. M., New York, N. Y.

Ingersoll, L. P., Keokuk, Iowa.
Inglis, Otto E., Philadelphia, Pa.
Ivory, J. W., Philadelphia, Pa.

Jefferis, C. R., Wilmington, Del.
Jenison, M. G., Minneapolis, Minn.
Jewell, M. D., Richfield Springs, N. Y.
Johnson, H. H., Hawkinsville, Ga.

Keely, H. A., Portland, Maine.
King, James R., Pittsburg, Pa.
Kingsbury, C. A., Philadelphia, Pa.
Kinsman, E. O., Cambridge, Mass.
Kirk, E. C., Philadelphia, Pa.
Klump, G. W., Wilkesbarre, Pa.
Koch, C. R. E., Chicago, Ill.
Kratzer, C. V., Reading, Pa.
Kuhn, G. P. E., Paris, France.

Leach, E. G., Boston, Mass.
Leonard, L. D., Minneapolis, Minn.
Levy, F. A., Orange, N. Y.
Libbey, J. A., Pittsburg, Pa.
Lovejoy, George W., Montreal, Canada.

Macleod, W. B., Edinburgh, Scot.
Magee, ——, St. Johns, New Brunswick.
Magill, W. E., Erie, Pa.
Marshall, J. S., Chicago, Ill.
Martindale, J. H., Minneapolis, Minn.
Maxfield, G. A., Holyoke, Mass.
McElhaney, G. W., Columbus, Ga.
McQuillen, D. N., Philadelphia, Pa.
Miller, W. B., Altoona, Pa.
Miller, W. D., Berlin, Ger.
Morey, J. G., New York, N. Y.
Morgan, W. H., Nashville, Tenn.
Morrison, W. N., St. Louis, Mo.

Newton, R. C., Mount Clair, N. J.
Northrop, A. L., New York, N. Y.

Ottolengui, B. A. R., New York, N. Y.

Palmer, S. B., Syracuse, N. Y.
Parmele, G. L., Hartford, Conn.
Parr, H. A., New York, N. Y.
Parramore, T. H., Hampton, Va.
Patterson, J. D., Kansas City, Mo.
Peirce, C. N., Philadelphia, Pa.
Perry, S. G., New York, N. Y.
Pinney, W., Newark, N. J.
Place, B. F., Philadelphia, Pa.
Pond, V. C., Boston, Mass.
Pruyn, C. P., Chicago, Ill.

Quick, E. P., Santa Barbara, Cal.

Register, H. C., Philadelphia, Pa.
Rhein, M. L., New York, N. Y.
Robb, G. L., Huntingdon, Pa.
Roberts, H. E., Philadelphia, Pa.
Rollins, W. H., Boston, Mass.
Root, G. F., Philadelphia, Pa.
Ross, F. R., Cedar Rapids, Iowa.
Russell, J. W., Brooklyn, N. Y.

Sanger, R. M., Brick Church, N. J.
Shepard, L. D., Boston, Mass.
Smith, B. H., Baltimore, Md.
Smith, C. S., Chicago, Ill.
Smith, E. H., Boston, Mass.
Smith, H. A., Cincinnati, Ohio.
Smith, L. S., Pittsburg, Pa.
Spinks, E. E., Meridan, Miss.

Stockton, C. S., Newark, N. J.
Story, J. C., Dallas, Texas.
Sudduth, W. X., Philadelphia, Pa.

Taft, J., Cincinnati, Ohio.
Taylor, L. C., Hartford, Conn.
Teague, B. H., Aiken, S. C.
Tees, Ambler, Philadelphia, Pa.
Thayer, W. J., Hoboken, N. J.
Thomas, J. D., Philadelphia, Pa.
Thomas, C., Des Moines, Ia.
Thompson, A. H., Topeka, Kan.
Thornton, R. W., Dalton, Ga.
Townsend, J. C., Philadelphia, Pa.
Truex, W. E., Freehold, N. J.
Trueman, W. H., Philadelphia, Pa.
Truman, Jas., Philadelphia, Pa.

Walker, W. W., New York, N. Y.
Ward, J. R. C., Philadelphia, Pa.
Wardlaw, W. C., Augusta, Ga.
Waters, G. F., Boston, Mass.
Waters, T. S., Baltimore, Md.
Watkins, S. C. G., Mount Clair, N. J.
Weatherby, I. J., Boston, Mass.
Weeks, T. E., Minneapolis, Minn.
Werner, J. G. W., Boston, Mass.
Wheeler, H. C., Springfield, Mass.
White, J. C., Sewickley, Pa.
White, S. A., Savannah, Ga.
Willey, J. J., Wahoo, Neb.
Williams, J. L., Boston, Mass.
Wilson, C. P., Boston, Mass.
Winder, R. B., Baltimore, Md.
Woodburn, J. C., Glasgow, Scotland.
Woodward, W. A., New York, N. Y.

Van Woert, F. T., Brooklyn, N. Y.

### ILLUSTRATIONS.

Abnormal incisors, L. C. Taylor, Fig. 1, 647.
restored, Fig. 2, 648.
spaces, S. H. Guilford, Figs. 1–5, 150, 154.
inferior central incisors, Fig. 4, 154.
central and lateral incisors, Fig. 3, 153.
superior central incisors, Fig. 1, 150.
Appliance for drawing back incisors, W. G. A. Bonwill, Fig. 6, 20.
for moving cuspids, E. H. Angle, Fig. 10, 329.
for rotation of incisors, E. H. Angle, Fig. 9, 329.
for moving cuspids, E. H. Angle, Fig. 10, 329.
for rotation of central incisors, E. H. Angle, Figs. 7, 8, 9, 328.
Application of sliding bars, W. G. A. Bonwill, Fig. 15, 79.
Arch-expander, W. G. A. Bonwill, Fig. 13, 78.
Artificial caries, transverse section, Geo. S. Allan, photo-micrographs, frontispiece, March, No. 6, facing 129.
Bacteria, groupings of, Geo. S. Allan, photo-micrographs, frontispiece, March, No. 1, facing 129.
pure cultures, facing 129.
Bands with clasp and spiral spring, W. G. A. Bonwill, Figs. 3, 4, 19.
Bar and clasps, W. G. A. Bonwill, Figs. 9, 10, 21, 207.
application of, Fig. 20, 82.
on squared temporary molar, Fig. 23, 84.
and ligatures, application of, Fig. 16, 80.
Bonwill's devices for reducing nerve-broaches, Figs. 1, 2, 3, 488.
Border-land of calcification, photo-micrograph, R. R. Andrews, frontispiece, April, facing 193.
Brace for support of loose teeth, Geo. S. Allan, 722.
Bridge-work, anterior teeth prepared for split post, T. S. Waters, Fig. 4, 201.
applied, Fig. 9, 203.
cast of partial case, T. S. Waters, Fig. 1, 200.
piece with split pins, Fig. 5, 201.
soldered to tooth cap, Fig. 7, 202.
with split post, ready for application, Fig. 8, 202.

Bridge-work, piece with spring catch, Fig. 2, 200.
applied, Fig. 3, 200.
Bubbles of gas in tubuli, having appearance of micrococci, photo-micrograph, R. R. Andrews, frontispiece, September, Fig. 6, facing 511.

Calco-globulin, photo-micrograph, R. R. Andrews, frontispiece, April, Figs. 1, 4, 5, 6, facing 193.
Calco-spherites, Fig. 2, facing 193.
Cap and ligatures in regulating, E. H. Angle, Fig. 5, 327.
Caries, artificial, distended tubules in, Geo. S. Allan, photo-micrograph, frontispiece, March, Nos. 5, 6, facing 129.
natural, Nos. 3, 4, facing 129.
cross section of, No. 4, facing 129.
long section of, No. 3, facing 129.
pictorial history of, facing 129.
Cavity in porcelain tooth, H. A. Keely, labial aspect, Fig. 1, 509.
lingual aspect, Fig. 2, 509.
undercuts, Fig. 3, 509.
Central incisor, prepared for fillings, J. N. Farrar, Fig. 6, 267.
protrusion of, E. H. Angle, Fig. 1, 323.
W. G. A. Bonwill, Fig. 18, 81.
S. H. Guilford, Fig. 2, 151.
simple rotation of, appliance for, E. H. Angle, Fig. 9, 328.
Cracks between cusps, photo-micrograph, R. R. Andrews, frontispiece, September, Fig. 1, facing 511.
cross section of calf tooth at birth, photo-micrograph, R. R. Andrews, frontispiece, April, Figs. 1, 3, 5, 6, facing 193.
Crown-work, natural crown on prepared root, Theodore F. Chupein, Fig. 4, 583.
plate with staples, Theodore F. Chupein, Fig. 3, 583.
punched plate for root, Theodore F. Chupein, Fig. 2, 583.
root prepared for crown, Theodore F. Chupein, Fig. 1, 582.
Cuspid grooved in regulating, W. G. A. Bonwill, Fig. 22, 83.
hook appliance for moving, E. H. Angle, Fig. 10, 329.
misplaced, W. G. A. Bonwill, Fig. 12, 77; Fig. 17, 80.
S. H. Guilford, Fig. 1, 401.

Decay between enamel and dentine, photo-micrograph, R. R. Andrews, frontispiece, September, Fig. 2, facing 511.
Dental caries, George S. Allan, photo-micrographs, frontispiece, March, facing 129.
Dentine, formed, R. R. Andrews, photo-micrograph, frontispiece, April, Figs. 1, 2, 4, 5, 6, facing 193.
forming band of, Fig. 3, facing 193.
sections of, George S. Allan, Nos. 2, 3, 4, 5, 6, facing 129.
Diagram, Bonwill's method of packing gold, E. C. Kirk, 522.
Distended tubules in artificial caries, George S. Allan, photo-micrographs, frontispiece, March, Nos. 5, 6, facing 129.
in natural caries, Nos. 3, 4, facing 129.
Double rotation of central incisors, appliance for, E. H. Angle, Figs. 7, 8, 328.

Expander for lower arch, W. G. A. Bonwill, Fig. 13, 78.
Extension-bar in retaining appliances, S. H. Guilford, Figs. 5, 6, 404.

Fan-shaped protrusion of central incisors, S. H. Guilford, Fig. 2, 151.
Farrar's spray-syringe, Fig. 3, 459.
Fissure in recently erupted tooth, photo-micrograph, R. R. Andrews, frontispiece, September, Fig. 3, facing 511.

Grooved temporary cuspids in regulating, W. G. A. Bonwill, Fig. 22, 83.
Gutta-percha stay-plate, W. G. A. Bonwill, Figs. 11, 21, 87.
application of, W. G. A. Bonwill, Fig. 17, 80.

Healthy dentine, sections of, Geo. S. Allan, photo-micrograph, frontispiece, March, No. 2, facing 129.

Interglobular spaces, photo-micrographs, R. R. Andrews, frontispiece, September, Fig. 4, 511.
Irregularity from pyorrhœa alveolaris, George S. Allan, Fig. 1, 723.
front view, Fig. 2, 725.
after correction, Figs. 3, 4, 725.

Jacksprings, W. G. A. Bonwill, Fig. 7, 20.
Jets and spray from Farrar's syringe, Fig. 2, 458.

Line of infection towards pulp, photo-micrograph, R. R. Andrews, frontispiece, September, Fig. 5, facing 511.

Misplaced cuspid, W. G. A. Bonwill, Fig. 12, 77; Fig. 17, 80.
S. H. Guilford, Fig, 1, 401.
Metal plate, half-clasps and spiral spring, W. G. A. Bonwill, Fig. 5, 19.

Natural caries, cross sections of, George S. Allan, photo-micrograph, frontispiece, March, No. 4, facing 129.
long sections of, No. 3, facing 129.
crown on prepared root, Theodore F. Chupein, Fig. 4, 583.

Odontoblastic layer and pulp tissue, photo-micrograph, R. R. Andrews, frontispiece, April, Figs. 1, 3, 4, 5, 6, facing 193.
Overbite, W. G. A. Bonwill, Fig. 1, 14.

Permanent tooth as fulcrum in regulating, W. G. A. Bonwill, Fig. 12, 77.
Philosophy of excavating a tooth, J. N. Farrar, Fig. 8, 269.
Photo-micrographs, border-land of calcification, R. R. Andrews, frontispiece, April, facing 193.
Pits and fissures of the enamel, R. R. Andrews, frontispiece, September, facing 511.
Pits on approximal surface of teeth, photo-micrograph, R. R. Andrews, frontispiece, September, Fig. 2, facing 511.
Pits on prominences of the cusps, Fig. 5, facing 511.
Plate to open bite, W. G. A. Bonwill, Fig. 9, 81.
Platinized gold bar with holes for ligatures, W. G. A. Bonwill, Fig. 8, 20.
Porcelain tooth, cavity in labial aspect of, H. A. Keely, Fig. 1, 509.
cavity in lingual aspect with shoulders, H. A. Keely, Fig. 2, 509.
undercut, in cavity, H. A. Keely, Fig. 3, 509.
Prominent superior teeth, E. H. Angle, Fig. 1, 323.
W. G. A. Bonwill, Fig. 18, 81.
S. H. Guilford, Fig. 2, 161.

Regulating appliances, E. H. Angle, Figs. 1–10, 323–329.
W. G. A. Bonwill, Figs. 1–11, 14–21; Figs, 12–23, 77–84.
J. N. Farrar, Figs. 1–8, 265–269.
S. H. Guilford, Figs. 1–6, 401–404.
Removable bridge-work, T. S. Waters, Figs. 1–9, 200–203.
Restoration of abnormal incisors, L. C. Taylor, Fig. 2, 648.
Retaining appliance, S. H. Guilford, Figs. 5, 6, 404.
pegs in regulating, J. N. Farrar, Figs. 1, 2, 265.
Rotation of incisors, E. H. Angle, Figs, 8, 9, 328.

Screw retaining pegs, J. N. Far rar, Figs. 1 2, 265.
Sections of dentine, Geo. S. Allan, photo-micrographs, frontispiece, March, Figs. 2–6, facing 129.
Separator, W. A. Woodward, Fig. 1, 89.
Shell caps, L. C. Taylor, Fig. 2, 648.
Single rotation of central incisor, E. H. Angle, Fig. 9, 328.
Sliding bars, in regulating, W. G. A. Bonwill, Fig. 14, 78.
application of, Fig. 15, 79.
W. A. Woodward, Fig. 2, 89.
Spiral spring, W. G. A. Bonwill, Fig. 2, 19.
Splint for implanted tooth, E. H. Angle, 379.
Spray syringe, J. N. Farrar, Figs. 1–3, 458, 459.
section of bolt-gauge, J. N. Farrar, Fig. 1, 458.
Squared temporary molars in regulating, W. G. A. Bonwill, Fig. 21, 83.

Tooth and wart-plug, longitudinal section of, J. N. Farrar, Fig. 4, 266.
transverse section, Fig. 5, 267.
of human foetus, photo-micrograph, R. R. Andrews, frontispiecc, April, Fig. 2, facing 193.
Traction bar, E. H. Angle, Fig. 4, 325.
Tubuli and intertubular spaces, George S. Allan, photo-micrographs, frontispiece, March, No. 2, facing 129.

Undermining caries of enamel, photo-micrographs, R. R. Andrews, frontispiece, September, Fig. 1, facing, 611.

Wart-filling in regulating, J. N. Farrar, Fig. 3, 266.
-plug, section view of, J. N. Farrar Fig. 7, 268.

### The International Dental Journal

SUCCESSOR TO

### THE INDEPENDENT PRACTITIONER.

The JOURNAL enters upon its tenth volume under most favorable auspices. It has been considerably enlarged by the addition of three lines to each page and one full form of reading matter. A syndicate composed of over **one hundred prominent members of the dental profession** has been formed. Besides the stockholders, who are also contributors, **a full corps of Foreign Correspondents** will write regularly for the Journal.

The name has been changed in order to better indicate the scope of its enlarged sphere. The journal is the official organ of **The American Academy of Dental Science**, Boston, and **The Odontological Society of Penna.**, Philadelphia. Arrangements have been made for a **series of Original Articles** from the pens of some of the best writers on dental subjects, which will in no way fall short of those that have, in the past, won for it such **high rank as a Scientific Journal.**

**Dr. W. D. MILLER, Berlin, will continue his articles on Dental Pathology.**

**Dr. BARRETT, former editor, will, from time to time, contribute to its columns.**

The trend of dental therapeusis is towards antisepsis, and a series of articles on that subject will appear during the year by the editor. Especial attention will be given to the **Practical Phase of Dentistry.** Besides the reports of the societies above referred to, comprehensive abstracts from other prominent dental societies will appear.

It is the intention to make these reports a prominent feature of the Journal. A complete cross topical index will be arranged at the close of the year, which will contain the names and addresses of each person quoted.

**Only original matter will be published** in the journal; it consequently comes in competition with none.

**Entirely Independent** of all schools, cliques, and advertising firms, it will be outspoken in its judgment of professional matters. **Owned by dentists and published for dentists,** we feel safe in saying that it cannot help becoming **the** journal in circulation as it even now is in the character of its contents.

On account of the continued demand, we have decided to renew the magnificent offer of Dr. Stowell's work on the **Microscopic Structure of the Human Tooth,** which is published at the moderate price of six dollars each. We have made such arrangements with the publishers that we can furnish it in connection with a subscription to **The International Dental Journal for One Dollar and Fifty Cents. That is, to every one who will send in Four Dollars we will send the Independent Practitioner one year and a copy of this beautiful and useful atlas.**

This offer is intended only for subscribers in America. Foreign residents within the postal union must enclose fifty cents extra when the atlas will be sent at their risk.

As the book absolutely costs more than the sum for which it will be furnished, the money must accompany the subscription. Nor will the book be sold separately. It will only be sent in connection with a subscription to this journal.

The book will be sent by mail when the necessary postage (25 cents) is included, or if not by express at the expense of the subscriber.

All remittances should be sent to the Editor and Business Manager.

Dr. W. X. SUDDUTH,

1215 FILBERT ST., PHILADELPHIA, PA.

**Postal Orders, Postal Notes, or New York Drafts are the safest and most convenient.**

**Subscriptions begin January and July. Price, $2.50 per Year, in advance.**

